# *Solanum lycopersicum* AUXIN RESPONSE FACTOR 9 regulates cell division activity during early tomato fruit development

**DOI:** 10.1093/jxb/erv152

**Published:** 2015-04-16

**Authors:** Maaike de Jong, Mieke Wolters-Arts, Bernardus C. J. Schimmel, Catharina L. M. Stultiens, Peter F. M. de Groot, Stephen J. Powers, Yury M. Tikunov, Arnoud G. Bovy, Celestina Mariani, Wim H. Vriezen, Ivo Rieu

**Affiliations:** ^1^Department of Molecular Plant Physiology, Institute for Water and Wetland Research, Radboud University Nijmegen, Heyendaalseweg 135, 6525 AJ Nijmegen, The Netherlands; ^2^Computational and Systems Biology, Rothamsted Research, West Common, Harpenden, Hertfordshire, AL5 2JQ, UK; ^3^Plant Research International, Wageningen University & Research Plant Breeding, Droevendaalsesteeg 1, 6708PB Wageningen, The Netherlands

**Keywords:** Auxin, AUXIN RESPONSE FACTOR 9 (*ARF9*), cell division, fruit development, fruit size, tomato (*Solanum lycopersicum* L.).

## Abstract

The transformation of the ovary into a fruit after successful completion of pollination and fertilization has been associated with many changes at transcriptomic level. These changes are part of a dynamic and complex regulatory network that is controlled by phytohormones, with a major role for auxin. One of the auxin-related genes differentially expressed upon fruit set and early fruit development in tomato is *Solanum lycopersicum* *AUXIN RESPONSE FACTOR* 9 (*SlARF9*). Here, the functional analysis of this ARF is described. *SlARF9* expression was found to be auxin-responsive and *SlARF9* mRNA levels were high in the ovules, placenta, and pericarp of pollinated ovaries, but also in other plant tissues with high cell division activity, such as the axillary meristems and root meristems. Transgenic plants with increased *SlARF9* mRNA levels formed fruits that were smaller than wild-type fruits because of reduced cell division activity, whereas transgenic lines in which *SlARF9* mRNA levels were reduced showed the opposite phenotype. The expression analysis, together with the phenotype of the transgenic lines, suggests that, in tomato, ARF9 negatively controls cell division during early fruit development.

## Highlight

Here, a function for *Sl*ARF9, of the tomato ARF gene family, is defined and new insight provided into the mechanism by which auxin controls cell division and early fruit development.

## Introduction

The development of the closed carpel is thought to be one of the features that contributed to the evolutionary success of the angiosperms (Scutt *et al.*, 2006). The carpel is the female reproductive organ that differentiates into stigma, style, and the ovary, the latter of which encloses the ovules. After successful completion of pollination and fertilization, the ovary develops into a fruit, with the ovary wall becoming the pericarp and the ovules developing into seeds. The fruit creates a protected environment for the seeds to mature and may mediate dispersal of the mature seeds (Gillaspy *et al.*, 1993). It is widely assumed that reproductive development occurs at the expense of vegetative growth (Snow and Whigham, 1989). Accordingly, the wild progenitors of many fruit crop species produce smaller fruits compared to the domesticated species (Tanksley, 2004; Doebley *et al.*, 2006). A good example is the tomato, with wild relatives such as *Solanum pimpinellifolium* bearing small fruit, and cultivated tomato species (*Solanum lycopersicum* L.) producing large fruit with a more than 100-fold increase in weight compared to wild species (Grandillo *et al.*, 1999; Tanksley, 2004). The tomato has been extensively used as a plant model species to study fruit development. Although research has mainly focussed on the later stages of fruit growth, processes occurring during fruit set and early stages of fruit development also have implications on the traits of the mature fruit, such as fruit size and shape (Paran and van der Knaap, 2007).

During flower development, cells at the floral meristems proliferate and differentiate to form the floral organs. The rate, duration, and direction of cell divisions in the developing ovary may already substantially impact final fruit size and shape (Bohner and Bangerth, 1988; van der Knaap *et al.*, 2014). When the ovary has reached its mature size, cell division activity stops. After a few days, the flower may abscise or, upon successful completion of pollination and fertilization, set fruit by resuming further cell division (Gillaspy *et al.*, 1993). This period continues for 10–14 days, and largely determines the final number of cells in the fruit (Bohner and Bangerth, 1988). In the next stage of development, fruit growth essentially depends on cell expansion, with cells increasing up to a 100-fold in volume (Tanksley, 2004). After this 6–7-week period the fruit has reached its final size and will start to ripen (Mapelli *et al.*, 1978; Bünger-Kibler and Bangerth, 1982; Gillaspy *et al.*, 1993).

Auxin plays an important role in tomato fruit set and fruit development. The auxin concentration in the ovary rapidly increases within 2 days after pollination (Mariotti *et al.*, 2011). The application of auxin on unpollinated ovaries leads to the formation of fruits without the need for pollination and fertilization (Gustafson, 1936; Bünger-Kibler and Bangerth, 1982). Similarly, affecting auxin synthesis or responsiveness by the ovary-specific expression of the *iaaM* or *rolB* genes from *Agrobacterium* spp. (Ficcadenti *et al.*, 1999; Carmi *et al.*, 2003) or the overexpression of the auxin receptor *TRANSPORT INHIBITOR RESPONSE* 1 (*TIR1*) (Ren *et al.*, 2011) resulted in the formation of seedless tomato fruits. Down-regulation of transcription factors involved in the regulation of auxin-mediated gene expression, like Aux/indole-3-acetic acid (IAA) 9 and AUXIN RESPONSE FACTOR 7 (ARF7) also results in fruit development without the need for pollination and fertilization (Wang *et al.*, 2005*a*; de Jong *et al.*, 2009). Transgenic lines in which *ARF7* transcript levels were reduced produced fruits with a thick pericarp, due to an increase in cell expansion, and formed a pointy tip at the blossom end of the fruit, demonstrating the effects of cell division and expansion that occur early in development on both size and shape of the mature fruit.

The regulatory role of auxin during the early stages of tomato fruit development is also demonstrated by the rapid increase in expression of auxin-related genes after pollination and fertilization. Previously, cDNA-amplified fragment length polymorphism–based transcript profiling (cDNA-AFLP) has been used to identify genes that are differentially expressed during tomato fruit set (Vriezen *et al.*, 2008). One of the genes induced by pollination appeared to be the putative tomato orthologue of the *Arabidopsis ARF9* gene, *Solanum lycopersicum ARF9* (*SlARF9*). Here, a functional analysis of this member of the tomato *ARF* gene family is described. The phenotypes of transgenic plants with either increased or reduced transcript levels of *SlARF9* indicate that *Sl*ARF9 negatively controls cell division during early fruit growth.

## Materials and methods

### Plant materials and growth conditions

Tomato plants (*S. lycopersicum* L. ‘Moneymaker’) were grown as described in de Jong *et al.* (2009). For expression analysis of *SlARF9* in ovaries, flowers were emasculated 3 days before anthesis. Hand pollination or hormone treatments were carried out at the stage of anthesis. *SlARF9* expression under the influence of auxin was analysed in ovaries of flowers treated with 2 µL of 1mM 4-Cl-IAA (Sigma-Aldrich, http://www.sigmaaldrich.com) in 2% ethanol. The treatment was repeated 6h after the first application. Control flowers were collected at the stage of anthesis. For analysis of *SlARF9* and *SlARF9B* expression in the transgenic lines, pericarp tissue was collected from ovaries and fruit that were formed by the second generation (T2) of the *SlARF9* overexpression (*SlARF9*-OE) lines, and the first generation (T1) of *SlARF9*-RNAi lines. The pericarp of fruits 3–4mm in diameter from the same lines was collected for the transcript profiling analysis. All collected tissues were frozen in liquid N_2_ and stored at −80°C until RNA extraction.

### RNA isolation, cDNA synthesis, and real-time quantitative PCR

Total RNA was extracted from the frozen tomato plant tissues with the TRIzol Reagent (Invitrogen, http://www.invitrogen.com), using a standard protocol from Invitrogen (Chomczynski and Mackey, 1995). Equal amounts of RNA were treated with RNase-free DNase I (Fermentas, http://www.fermentas.com), and used as a template for cDNA synthesis (iScript^TM^ cDNA synthesis Kit, Bio-Rad, http://bio-rad.com). For real-time quantitative PCR the same conditions were used as described by de Jong *et al.* (2009). The primers were as follows: *SlARF9* (Solyc08g082630), forward 5ʹ-CGTAGGCGTCAACAAATACTTAGAGG-3ʹ, reverse 5ʹ-TCCACTGTGAAGAAAGATCATCAATTCC-3ʹ; *SlARF9B* (Solyc08g008380), forward 5ʹ-TTGCGTCCTCACAATTCGGAAAGC-3ʹ, reverse 5ʹ-CCAGAGCACCCTTCAGCAGAGC-3ʹ. As reference genes *SlActin2/7* (forward 5ʹ-GGACTCTGGTGATGGTGTTAG-3ʹ, reverse 5ʹ-CCGTTCAGCAGTAGTGGTG-3ʹ, based on SGN-U579547), and *SlUbi7* (forward 5ʹ-CCCTGGCTGATTACAACATTC-3ʹ, reverse 5ʹ-TGGTGTCAGTGGGTTCAATG-3ʹ, based on SGN- U576276) were used. Each real-time quantitative PCR experiment was based on two technical and two biological repeats, except for the verification of the *SlARF9* expression pattern as obtained from the cDNA-AFLP analysis (Vriezen *et al.*, 2008) for which only one biological series was used.

### Isolation of the *SlARF9* promoter sequence

Genomic DNA was isolated from leaf tissue to generate a *SnaI* (Fermentas) GenomeWalker tomato library (GenomeWalker Universal Kit, BD Biosciences, http://www.bdbiosciences.com). The use of gene-specific primer 5ʹ-TTCTTCAGCCAGGAAATGACTATTGATAACTCG-3ʹ and nested primer 5ʹ-GGAGAATTCATATTCGGCTGAGAC-3ʹ resulted in the isolation of a 3kb fragment corresponding to the *SlARF9* promoter. The Erase-a-Base system (Promega, http://www.promega.com) was used to generate subclones containing progressive unidirectional deletions of this fragment. Subsequently, these subclones were sequenced and aligned using ClustalW (http://www.ebi.ac.uk/clustalW).

### Plant transformation

To generate fruit-specific *SlARF9* overexpression lines, the coding sequence of *SlARF9* (forward 5ʹ-CACCATGGCAACTATAAATGGGTGGTG-3ʹ, reverse 5ʹ- TTAACTGTCTGCGCGAGACAGGG-3ʹ) was cloned into the pENTR/D-TOPO entry vector (Invitrogen). This clone was recombined with the pARC983 binary vector, in which the cauliflower mosaic virus (CaMV) *35S* promoter was replaced for the ovary- and young fruit-specific *TPRP-F1* promoter (Czerednik *et al.* 2012). For the generation of the *SlARF9*-RNAi lines, a fragment of the *Sl*ARF9 mid-region (amino acids 367–506, forward 5ʹ-AAAAAGCAGGCTGTCCCACCAACCGCAGAGAAGAAC-3ʹ; reverse 5ʹ-AGAAAAGCTGGGTGCTGTAGTCGTGCCTCAGTAGTGC-3ʹ) was cloned into the pDONR221 entry vector (Invitrogen), which was subsequently recombined with the binary vector pK7GWIWG2(I) (Karimi *et al.*, 2002) in both sense and antisense orientation under the transcriptional regulation of the CaMV *35S* promoter and terminator. To generate the p*SlARF9*::GUS (β-glucuronidase) lines, the promoter fragment of *SlARF9* (2200bp, forward 5ʹ-CACCTTTTCAAAGAGGTGTGACATTTTCAATAAC-3ʹ; reverse 5ʹ-CAACCTTCAATTCCAAAAACTAAAGAACACCC-3ʹ) was cloned into the pENTR/D-TOPO entry vector. This entry clone was recombined with the destination vector pKGWFS7 (Karimi *et al.*, 2002).

The transgenic tomato plants were generated by *Agrobacterium tumefaciens*–mediated transformation, as described in de Jong *et al.* (2009). Although grown on kanamycin-containing medium, possible escapes were detected by PCR with primers specific for the kanamycin-resistance gene (forward 5ʹ-GACTGGGCACAACAGACAATCG-3ʹ, reverse 5ʹ-GCTCAGAAGAACTCGTCAAGAAGG-3ʹ) on genomic DNA. Subsequently, lines were tested for tetraploidy, as only diploid lines were used for further analysis.

### Histochemical analysis of GUS activity

Tissues of first-generation adult plants (T1) and 15-day-old seedlings (T2) of the p*SlARF9*::GUS lines were submerged in GUS-staining buffer containing 0.1% Triton X-100, 0.5mM Fe^2+^CN, 0.5mM Fe^3+^CN, 10mM EDTA, 1mg mL^-1^ X-Gluc, 0.1mg mL^-1^ in 50mM phosphate buffer, pH 7.0. After incubation at 37ºC, the tissues were cleared with 70% ethanol and viewed under a stereomicroscope (Leica MZFL III, Leica Microsystems, http://leica-microsystems.com). For detailed analysis of lateral roots and ovules by light microscopy, the GUS-stained tissues were embedded in Technovit 7100 (Heraeus Kulzer, http://www.heraeus-kulzer.com). The embedded tissues were sliced into sections of 5 μm. The sections of the lateral roots were counterstained with 0.5% safranine, and subsequently partly de-stained with 70% ethanol. The sections were viewed under a Leitz Orthoplan microscope (Leica Microsystems). Images were made with a Leica digital camera (model DFC 420C; Leica Microsystems).

### Quantification of cell area and number of cell layers

Pericarp tissues of fruits 7–8mm in diameter were fixed in a 2% glutaraldehyde in 0.1M phosphate buffer, pH 7.2, overnight at 4ºC. Subsequently, the tissues were dehydrated in an ethanol series and embedded in Spurr resin. Sections of 1 μm were stained with a toluidine blue solution (0.1% in 1% borax). Pericarp tissue of mature fruits at the breaker stage were fixed in formalin–acetic acid–alcohol solution (3.7–4.1% formaldehyde solution, 5% acetic acid, and 50% ethanol), dehydrated in an ethanol series, and subsequently embedded in Technovit. Sections of 5 μm were stained with a toluidine blue solution. The sections were viewed under a Leitz Orthoplan microscope (Leica Microsystems), and micrographs were made with a Leica digital camera (model DFC 420C; Leica Microsystems). These micrographs were used for further analysis.

For analysis of the 7–8mm fruits, square sections of 0.16mm^2^ were delimited and positioned approximately 0.1mm from the inner pericarp, including the epidermal layer. For analysis of the mature fruits, sections of 9mm^2^ were delimited and positioned approximately 1mm from the inner pericarp. Then the total number of cells inside these squares was counted. Cells that were positioned with two-thirds or more of their size in the sections were included. For estimation of the number of cell layers within the pericarp, a line was drawn across the pericarp sections. The number of cells along this line, including cell layers of the epidermis and the three distinct layers of the pericarp (exocarp, mesocarp, and endocarp) were scored (Supplementary Fig. S1). In total, one region per fruit and 4–15 fruits per line were analysed, deriving from 5–12 (7–8mm fruits) or 5–15 (mature fruit) plants per line.

### Statistics

For statistical analysis of quantitative PCR data, log_2_-transformed, reference-gene-corrected Ct values were used (Rieu and Powers, 2009). ANOVA was applied, with a Tukey’s post-hoc test being invoked when multiple comparisons of tomato lines were made for a given (target) gene.

For the statistical analysis of the different phenotypic traits, fruits of 7–8mm and at breaker stage were collected from multiple plants of the different transgenic and wild-type lines arranged in four complete statistical blocks. Because the number of fruit collected for each transgenic line differed per block, the imbalance precluded use of ANOVA and so the method of residual maximum likelihood was used to fit a linear mixed model to the data, taking into account the blocks as a random effect. Where necessary, the method also took into account the nesting of fruit within clusters and clusters within plants as random effects, so that comparison of transgenic lines was based on the correct degrees of freedom for underlying residual (plant-to-plant) variation. A log (to base *e*) transformation was used for fruit weight to account for some heterogeneity of variance across the lines. Means for the lines were compared using standard error of the difference (SED), invoking the least significant difference values at the 5% or 1% level of significance. The GenStat (14^th^ edition, VSN International Ltd, Hemel Hempstead, UK) was used for this analysis.

### Microarray data analysis

The transcript profiling analysis was done using pericarp tissue from fruits 3–4mm in diameter, with each sample containing the pericarp of two fruits. For each separate line, tissues were collected from two to three plants, resulting in a total of six samples for *SlARF9*-OE, seven samples for *SlARF9*-RNAi, and two samples for wild type. Total RNA was extracted as described for real-time quantitative PCR. To synthesize cDNA, 100ng of total RNA was used with the Ambion WT expression kit (Applied Biosystems/Life Technologies, Nieuwerkerk aan den IJssel, The Netherlands). Subsequently, the cDNA was labelled with biotin, using the Affymetrix GeneChip WT Terminal Labeling Kit (Affymetrix, Santa Clara, CA, USA), and hybridized to the Affymetrix EUTOM3 tomato exon arrays. The microarray signals were determined using MadMax microarray analysis software (hhtp://madmax.bioinformatics.nl). Data were deposited in the NCBI GEO repository, accession number GSE63637. Further analysis was performed using GeneMaths XT microarray data analysis software (Applied Maths, http://www.applied-maths.com/genemaths-xt). Prior to analysis, the data was normalized using 2log transformation, followed by mean subtraction scaling. Student’s *t*-test and principal component analysis (PCA) analyses were performed in GeneMaths XT and PAST3 (http://folk.uio.no/ohammer/past/). The Pearson correlation coefficients were calculated using the corresponding function of Microsoft Office Excel 2010. Pairwise comparison between the transcriptomes of wild-type, *SlARF9*-OE, and *SlARF9*-RNAi fruits (*t*-test, *P* < 0.05) resulted in a list of 753 differentially expressed genes (Supplementary Table S1).

All genes represented on the array, with a maximum expression value of >10 across the samples, were classified according to the MapMan functional categories (Thimm *et al.*, 2004), which have been assigned based on the ITAG2.3 genome annotation (http: //mapman.gabipd.org). The distribution of the functional categories was evaluated with gene set enrichment analysis (GSEA) (Subramanian *et al.*, 2005). The natural scale intensity data were used as the input for the GSEA (April 2014), and the genes were ranked based on the signal-to-noise metric. Gene sets with <10 and >500 members were ignored, leaving out 843 of the 1209 MapMan categories. With these settings, four gene sets with a false discovery rate (FDR) <25% were found to be up-regulated in the *SlARF9*-RNAi lines. Leading edge analysis was used to extract the genes that accounted for the gene set’s enrichment signal.

## Results

### Expression of *SlARF9*

The transcriptome of ovaries before and after fruit induction has previously been analysed to study the role of phytohormones in tomato fruit set (Vriezen *et al.*, 2008). One of the characterized transcripts specifically modulated after pollination encoded an ARF protein homologous to *Arabidopsis thaliana* ARF9 (GenBank Accession No. BT013639), and was therefore designated *SlARF9* (ITAG2.3 Solyc08g082630) (Zouine *et al.*, 2014). The derived protein sequence of *Sl*ARF9 contains 658 amino acids, and comprises the N-terminal B3-derived DNA binding domain (amino acids 74–236) and two C-terminal homo- and heterodimerization domains, III and IV (CTD, amino acids 566–602 and 609–651, respectively) that are typically present in ARFs (Supplementary Fig. S2) (Guilfoyle and Hagen, 2007). The middle region (MR) of ARF proteins, located between the DNA binding domain and CTD, functions as a transcriptional activation or repression domain depending on its amino acid composition (Ulmasov *et al.*, 1999*a*; Tiwari *et al.*, 2003). The MR of *Sl*ARF9 is enriched with serine, which represents 11.6% of amino acid residues, suggesting that this ARF may act as a transcriptional repressor.

**Fig. 1.**
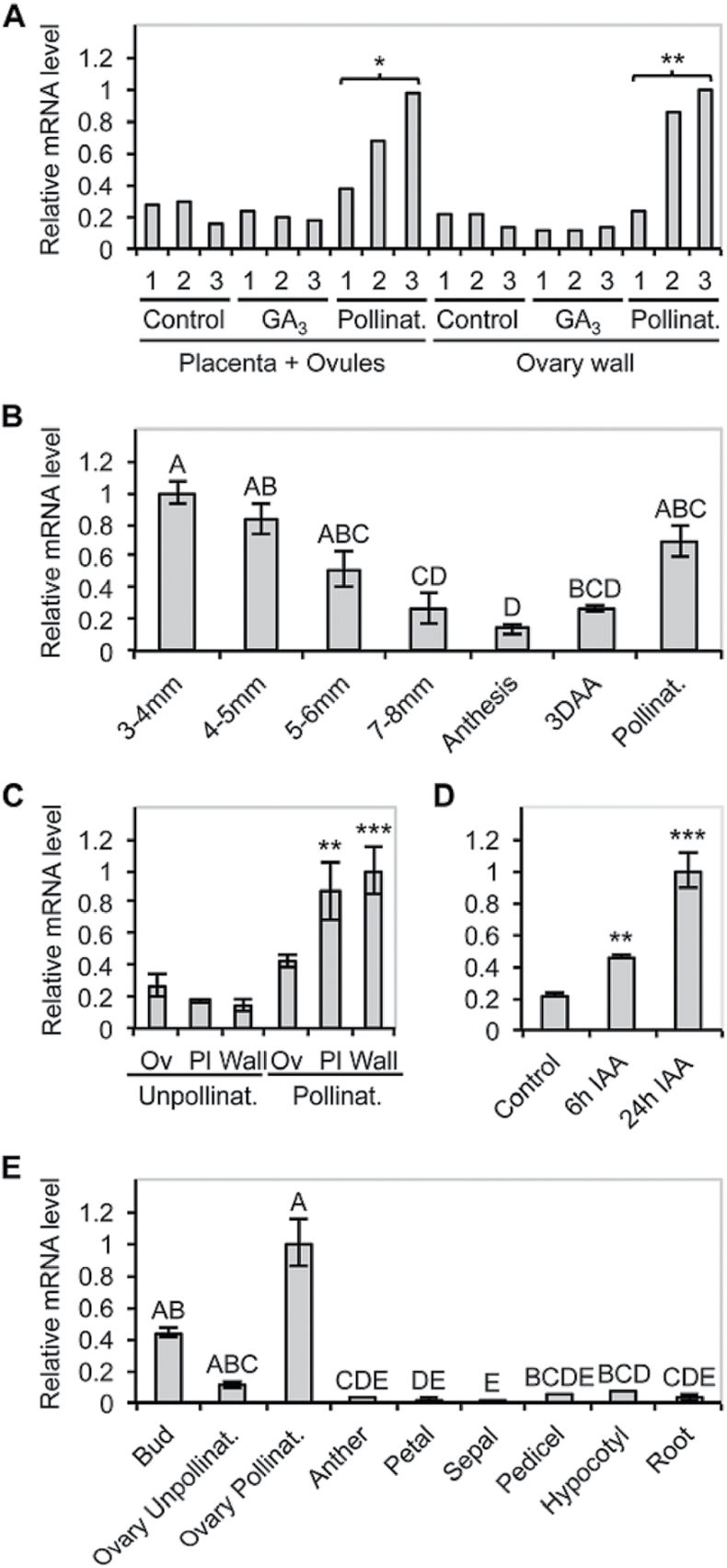
*SlARF9* mRNA levels during tomato fruit set. (A) Verification of the *SlARF9* expression pattern as obtained from the cDNA-AFLP analysis (Vriezen *et al.*, 2008) by real-time quantitative PCR on placenta together with ovular tissue and the ovary wall, at 1, 2 and 3 days after treatment. Total RNA was isolated from emasculated flowers (Control), emasculated flowers treated with gibberellic acid (GA_3_), and emasculated flowers after hand pollination (Pollinat.) (pools of 3–5 ovaries per sample). *significantly different from control treatment, *P* < 0.05; **P < 0.01. (B) Relative mRNA levels of *SlARF9* in tomato ovaries collected throughout different stages of flower development, at anthesis, from unpollinated flowers 3 days after anthesis (3DAA), and flowers 3 days after hand pollination (Pollinat.). Standard errors are indicated (*n* = two pools of 3–5 flowers). Capital characters above bars indicate homogenous categories (Tukey) with differences at *P* < 0.05. (C) Relative mRNA levels of *SlARF9* in unpollinated tomato ovaries at anthesis and ovaries collected 3 days after hand pollination, dissected into ovule (Ov), placenta (Pl), and ovary wall tissue (Wall) samples. Standard errors are indicated (*n* = two pools of 10 ovaries). **significantly different from the unpollinated control, *P* < 0.01; ***P < 0.001. (D) Relative mRNA levels of *SlARF9* in tomato ovaries of emasculated flowers collected 6 or 24h after auxin treatment (IAA). Untreated ovaries were used as a control. Standard errors are indicated (*n* = two pools of 3–5 ovaries). **significantly different from the control, *P* < 0.01; ***P<0.001. (E) Relative mRNA levels of *SlARF9* in young flower buds, unpollinated ovaries, and various other floral organs collected from flowers at the stage of emasculation, pollinated ovaries (3 DAP), and in the hypocotyl and root of 10-day-old seedlings. Standard errors are indicated (*n* = 2). Capital characters above bars indicate homogenous categories (Tukey) with differences at *P* < 0.05.

The transcript profiling described in Vriezen *et al.* (2008) showed that *SlARF9* expression increased within 48h after pollination, but not after treatment with gibberellin (GA). Furthermore, *SlARF9* was shown to be expressed in the placental and ovular tissues as well as the ovary wall. In the current study, these patterns were verified by real-time quantitative PCR (Fig. 1A). Expression analysis in ovaries collected at various stages of flower development showed that the *SlARF9* transcript was also highly abundant in the early stages of flower development. The transcript levels were reduced during the later stages of flower development, reaching the lowest level of expression at anthesis and remaining low unless successful pollination and fertilization occurred (Fig. 1B). These processes increased *SlARF9* expression mainly in the placental tissue and the ovary wall (Fig. 1C).

Although GA-treatment of unpollinated mature ovaries had no effect on *SlARF9* expression, treatment with IAA resulted in an increase of *SlARF9* transcript levels (Fig. 1D). *In silico* analysis of the 1.5kb promoter sequence for the presence of auxin-related *cis*-acting regulatory elements using PlantCARE (Lescot *et al.*, 2002) and PLACE (Higo *et al.*, 1999) software resulted in the identification of two degenerated auxin response elements (AuxREs; Supplementary Table S2). These elements are typically found in the promoter sequences of auxin response genes and are bound by the ARF transcription factors (Ulmasov *et al.*, 1999*b*). Furthermore, the *SlARF9* promoter sequence contains several NTBBF1ARROLB-elements. These elements were first identified in the promoter sequence of *rolB*, one of the oncogenes present in the T-DNA sequence of *Agrobacterium rhizogenes*, and are involved in the auxin-inducible expression of the *rolB*-gene in plants (Baumann *et al.*, 1999). Both AuxREs and NTBBF1ARROLB-elements are also present in the promoter regions of *SlIAA2* and *SlIAA14*. These are transcriptional repressors that regulate the expression of auxin-responsive genes. However, many *Aux/IAA* genes are auxin-inducible themselves (Reed, 2001). Similar to *SlARF9*, the expression of *SlIAA2* and *SlIAA14* was found to be up-regulated in ovaries by pollination (Vriezen *et al.*, 2008) and treatment with auxin (Supplementary Fig. S3).

**Fig. 2.**
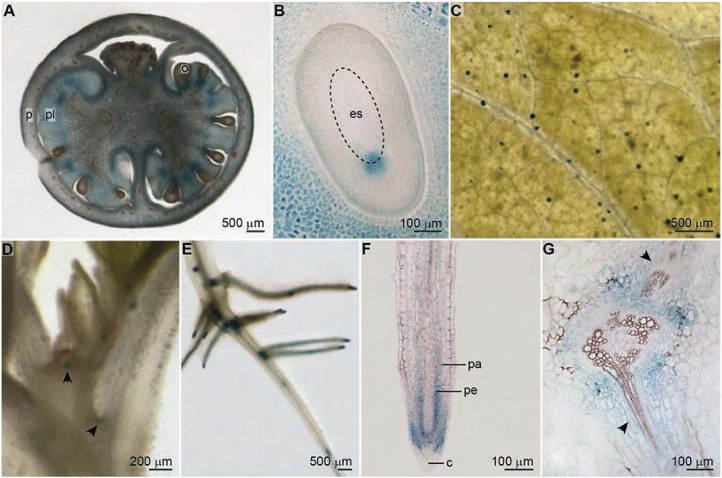
Histochemical GUS staining of *pSlARF9::GUS* tomato lines. (A) Tomato fruit, 5–6mm in diameter, corresponding to approximately 6 DAP. The GUS staining is visible in the ovules (o), placenta (pl), and pericarp (p). (B) Cross section of an ovule from a 5–6mm tomato fruit. The GUS staining is localized at the micropylar end of the embryo sac (es), which is encircled. (C) Glandular hairs and trichomes on the leaf surface. Only the glandular hairs showed GUS activity. (D) The apex of a 15-day-old seedling. GUS is expressed at the axillary meristems, at the base of the leaves (arrows). (E) Primary and lateral roots of a 15-day-old seedling. (F) Longitudinal section of a lateral root tip of a 15-day-old seedling. The GUS staining is located in the meristematic zone, but not in the columella (c). The pericycle (pe) and a few cell layers of parenchyma (pa) were also stained. (G) Longitudinal section through two emerging lateral roots (arrows).

To investigate the expression of *SlARF9* in more detail, an *SlARF9* promoter-GUS fusion was constructed using the 2200bp 5ʹ-end flanking sequence of *SlARF9* and the GUS-coding sequence of the *uidA* gene. Subsequently, this p*SlARF9*::*GUS* construct was introduced into tomato by *Agrobacterium*-mediated gene transfer. In seven out of the fourteen independent lines that were generated, *uidA*-expression was observed in tomato fruits of 5–6mm in diameter, corresponding to approximately 8 days after pollination (DAP). The GUS staining was visible in the pericarp, the outer cell layers of the placenta that develop into a gel-like substance, and in the ovules (Fig. 2A). Microscopic analysis of cross-sections through the ovules showed that the GUS staining was located at the micropylar end of the embryo sac, corresponding to the location of the suspensor or wall ingrowths that develop quickly around the base of the suspensor (Fig. 2B) (Briggs, 1995). The levels of *SlARF9* transcript were low in plant tissues other than the ovary (Fig. 1E). Nevertheless, GUS staining could also be seen in the glandular hairs at the surface of leaf and stem (Fig. 2C) and in the axillary shoot apical meristem (Fig. 2D). Furthermore, GUS staining was observed in the primary root tips, early lateral root primordia, and outgrowing lateral roots (Fig. 2E). Here, the staining was located in the meristematic zone of the root tips, the pericycle, and in a few cell layers of the parenchyma (Fig. 2F,G). These findings indicate that although *SlARF9* is predominantly expressed in the fruit, *Sl*ARF9 may also function in other tissues, mostly those in which many cell divisions occur.

### Overexpression and silencing of *SlARF9* have opposite effects on fruit size

To explore the physiological role of *Sl*ARF9 in tomato fruit set and development, transgenic tomato lines were generated in which the gene was overexpressed or silenced. For the production of the *SlARF9* overexpression lines (*SlARF9*-OE), the coding sequence of *SlARF9* was ligated to the *TPRP-F1* promoter, which is specific for the ovary and young fruit (Carmi *et al.*, 2003). From the 11 independent transgenic lines that were generated, the two *SlARF9*-OE lines with the highest expression, i.e. lines 4 and 5, were selected for further analysis. Transgenic tomato lines in which the *SlARF9* gene was silenced were generated by an RNAi approach, using a 420bp fragment based on the MR of *Sl*ARF9 (amino acids 367–506, Supplementary Fig. S2). This fragment was cloned into an RNAi binary vector, under the transcriptional regulation of the CaMV 35S promoter, and transferred to tomato by *Agrobacterium*-mediated transformation. In four out of the twelve generated transgenic lines the *SlARF9* transcript levels were reduced. These *SlARF9*-RNAi lines (numbers -1, -6, -9 and -12) were used for further analysis.

**Fig. 3.**
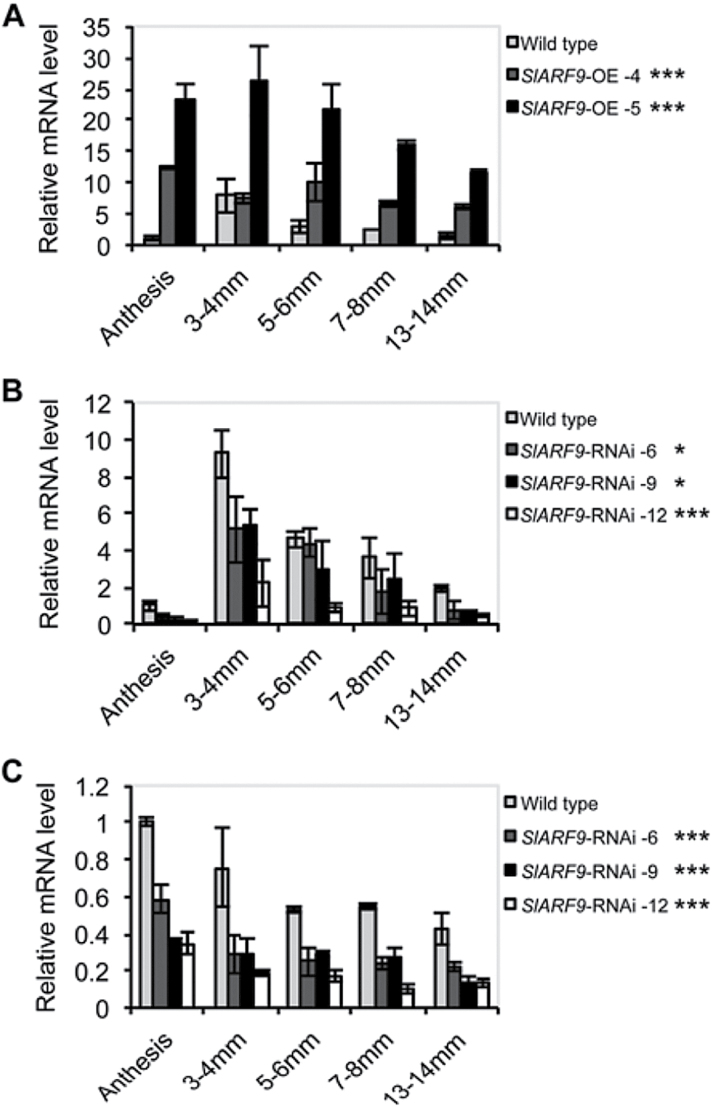
*SlARF9* and *SlARF9B* mRNA levels in developing wild-type and transgenic fruits. Relative mRNA levels of *SlARF9* in ovaries and fruits collected from (A) wild-type and *SlARF9*-OE lines, and (B) S*lARF9*-RNAi lines. (C) Relative mRNA levels of *SlARF9B* in wild-type and *SlARF9*-RNAi ovaries and fruits. Standard errors are indicated (*n* = two pools of 3–5 ovaries). Statistical analysis of *SlARF9* expression in the wild type (a,b) is presented in Table S2. *significantly different from the wild type, *P* < 0.05; *** *P* < 0.001.

Expression analysis of *SlARF9* during several early stages of fruit development showed that in wild type the relative mRNA level of *SlARF9* rapidly increased after pollination and fertilization, and was highest in fruits of 3–4mm in diameter, corresponding to 6 DAP. In subsequent stages the transcript levels decreased again (Fig. 3A,B; Supplementary Table S3). In the *SlARF9*-OE lines, *SlARF9* transcript levels were already high at anthesis independently of pollination, and remained high for a longer period of time than in wild-type fruits (Fig. 3A). In the *SlARF9*-RNAi lines the expression pattern of *SlARF9* was similar to that in wild type, but the overall transcript level was reduced by 40–70% and most prominently in the 3–4mm fruit (Fig. 3B). Recently, Zouine *et al.* (2014) identified 22 putative functional *ARF* genes in the tomato genome. The authors’ phylogenetic analysis showed that *SlARF9* clusters with another *ARF* gene located on chromosome 8, referred to as *SlARF9B* (ITAG2.3 Solyc08g008380). Although closely related to *SlARF9*, the expression of *SlARF9B* in the ovary was not affected by pollination (Fig. 3C). The specificity of the 420bp fragment of *SlARF9* used to generate the *SlARF9*-RNAi lines was tested by genomic DNA Southern blot analysis, which resulted in a single strong hybridization signal (Supplementary Fig. S4). However, analysis of *SlARF9B* expression in ovaries and young fruits collected from the *SlARF9*-RNAi lines showed that the transcript level of *SlARF9B* was decreased compared to that in wild type (Fig. 3C). The *SlARF9* fragment used for generation of the RNAi lines contained three stretches of 21–23 nucleotides highly similar to the sequence of *SlARF9B*, only containing one or two mismatches. It is possible that these fragments brought about the degradation of the *SlARF9B* mRNA.

Although the RNAi construct was under regulation of the constitutive 35S promoter, no obvious vegetative phenotypes were observed. By contrast, both *SlARF9*-OE and *SlARF9*-RNAi lines showed a clear and opposite phenotype in the fruit. Overexpression of *SlARF9* resulted in a reduction in the final size and weight of fruits, as determined at breaker stage (Table 1). *SlARF9* silencing, on the other hand, resulted in bigger and heavier fruit as compared to the wild type (Table 1, Supplementary Fig. S1D).

**Table 1.** Analysis of fruit size and weight of mature wild-type and transgenic fruits, as determined at breaker stage

Line	Width (cm)	*P* (line)	*P* (type)	Height (cm)	*P* (line)	*P* (type)	Weight (g)	*P* (line)	*P* (type)
Wild type	5.685			5.220			84.775 (4.440)		
*SlARF9*-OE-4	5.332	***	**	3.718	***	***	64.457 (4.166)^a^	***	***
*SlARF9*-OE-5	5.482	ns		3.944	***		73.553 (4.298)	*	
*SlARF9*-RNAi-1	6.095	***	***	6.005	***	***	101.799 (4.623)	***	***
*SlARF9*-RNAi-6	6.357	***		6.118	***		107.878 (4.681)	***	
*SlARF9*-RNAi-9	6.184	***		6.044	***		108.636 (4.688)	***	
*SlARF9*-RNAi-12	5.962	ns					96.641 (4.571)	ns	
Maximum SED Degrees of freedom	0.1787 121			0.1835 62			0.0893 131		

To determine the width and weight of wild-type and transgenic fruit, fruits were collected from plants grown in one to four complete statistical blocks with 5–143 replicates per line per block. The height of the fruit was determined in two of the blocks with 18–143 replicates per genotype per block. The level of significance compared to wild-type fruits is indicated for each individual line and per type of transgenic line. *P < 0.05; **P < 0.01; ***P < 0.001; ns, not significant. ^a^ The means on the log (to base *e*) scale, are used for statistical comparisons.

### Modulation of *SlARF9* expression level affects cell expansion and division at early stage

To understand the cause of the *SlARF9*-dependent changes in fruit size, cell size was determined by examining the number of cells per surface unit, and number of cell layers in the pericarp of breaker stage fruits. The pericarp of the fruits of the *SlARF9*- silenced lines, which bore bigger fruits, had smaller average cell size (greater cells/mm^2^), but more cell layers (Table 2, Supplementary Fig. S1). In the *SlARF9*-OE lines, which bore smaller fruits, the opposite was seen because the pericarp in the fruits contained fewer cell layers. Cell size was not significantly (*P* < 0.05, least significant difference) increased in these lines compared to wild type.

**Table 2.** Quantification of cell number per surface unit and number of cell layers in the pericarp of mature wild-type and transgenic fruits, collected at breaker stage

Line	Cells/mm^2^	*P* (line)	*P* (type)	Cell layers	*P* (line)	*P* (type)
Wild type	6.192			27.53		
*SlARF9*-OE-4	5.676	ns	ns	24.39	*	*
*SlARF9*-OE-5	5.395	ns		23.96	*	
*SlARF9*-RNAi-1	7.391	ns	**	36.03	***	***
*SlARF9*-RNAi-6	7.307	ns		32.85	***	
*SlARF9*-RNAi-9	7.241	ns		34.40	***	
*SlARF9*-RNAi-12	9.755	***		34.13	***	
Maximum SED	1.1514			2.034		
Degrees of freedom	49			50		

To determine the cell number and number of cell layers in the pericarp, fruits were collected from plants grown in three complete statistical blocks with 2–7 replicates per genotype per block. The level of significance compared to wild-type fruits is indicated for each individual line and per type of transgenic line. *P < 0.05; **P < 0.01; ***P < 0.001; ns, not significant.

Several TILLING mutants with mutations in the *SlARF9* gene were also identified and analysed, but none of them had larger fruits than the corresponding wild type. One of the lines, *slarf9-1*, carried a C-to-T mutation translating into a histidine 179-to-tyrosine substitution in the middle of the B3 DNA binding domain. BLAST analysis showed that this amino acid is highly conserved among tomato ARFs and completely conserved among ARF9-related proteins up to the monocots. Although fruit weight of this mutant was normal (Table 3), cytological analysis showed it had significantly (*P* < 0.05, least significant difference) more cell layers in the pericarp at breaker stage than the wild type (Table 3).

**Table 3.** Quantification of fruit weight and number of cell layers in the pericarp of mature wild-type and slarf9-1 fruits, collected at breaker stage

Line	Weight (g)	*P*	Cell layers	*P*
Wild type	51.05		26.50	
*slarf9-1*	55.14	ns	30.17	*

*significantly different from wild type, *P* < 0.05; ns, not significant.

To test whether the *SlARF9*-modulated transgenic lines already differentiated from wild type in the earlier stages of fruit development, histological cross-sections of 7–8mm fruits were also analysed. In the pericarp of *SlARF9*-OE fruits, cells were on average bigger than in wild-type fruits, but they still contained a normal number of cell layers. In the *SlARF9*-RNAi lines, cells were smaller and the number of cell layers was already increased (Table 4, Supplementary Fig. S5).

**Table 4.** Quantification of cell number per surface unit and number of cell layers in the pericarp of wild-type and transgenic fruits, 7–8mm in diameter

Line	Cells/mm^2^	*P* (line)	*P* (type)	Cell layers	*P* (line)	*P* (type)
Wild type	779.2			25.77		
*SlARF9*-OE-4	585.1	*	**	26.41	ns	ns
*SlARF9*-OE-5	582.0	*		25.13	ns	
*SlARF9*-RNAi-1	1164.7	***	***	30.93	*	***
*SlARF9*-RNAi-6	1296.3	***		31.87	***	
*SlARF9*-RNAi-9	1077.0	**		30.66	**	
*SlARF9*-RNAi-12	1441.1	***		29.97	*	
Maximum SED	136.30			2.517		
Degrees of freedom	47			46		

To determine the cell number and number of cell layers in the pericarp, fruits were collected from plants grown in two complete blocks with 2–6 replicates per genotype per block. The level of significance compared to wild–type fruits is indicated for each individual line and per type of transgenic line. *P < 0.05; **P < 0.01; ***P < 0.001; ns, not significant.

### Transcriptional analysis of early fruit development in *SlARF9*-OE and *SlARF9*-RNAi lines

To identify possible transcriptomic changes associated with the fruit developmental phenotypes observed in the transgenic lines, the gene expression profiles of 3–4mm fruits from wild-type and transgenic plants were analysed. At this stage, the *SlARF9* transcript reached its maximum level in wild-type fruits (Fig. 3), but no phenotypic differences were observed between wild-type and transgenic fruits. The transcript profiling analysis was carried out using Affymetrix EUTOM3 tomato exon arrays.

The microarray analysis showed a 2.6-fold difference in *SlARF9* expression between wild-type and *SlARF9*-OE fruits (*P* < 0.001, Student’s *t*-test), and a 1.6-fold difference between wild-type and *SlARF9*-RNAi lines (*P* < 0.05, Student’s *t*-test), closely corresponding to the results obtained by real-time quantitative PCR analysis (Fig. 3). PCA of the normalized microarray data indicated that, overall, the transcriptomes of the different types were somewhat similar, but especially those of wild-type and *SlARF9*-OE fruit (Supplementary Fig. S6A). Comparable results were obtained when applying the PCA to only those genes with significantly different expression levels between any two types based on a per gene ANOVA test, as the first principal component (PC1) did not separate wild-type from OE plants (Supplementary Fig. S6B). Possibly, the *SlARF9*-dependent transcriptional responses were already nearly saturated in 3–4mm wild-type fruits.

Because the *SlARF9*-OE and *SlARF9*-RNAi fruits had opposite phenotypes, pairwise comparison between the transcriptomes of these lines was done. Unexpectedly, the analysis showed that only *SlARF9* expression levels remained significantly different between the two types after correcting for multiple comparisons (FDR = 0.03); adding the next most significant group of 14 genes raised the FDR to 0.50. Therefore, instead of looking at individual genes, GSEA was applied to the whole dataset (Subramanian *et al.*, 2005). Using MapMan functional gene categories as gene sets (Thimm *et al.*, 2004), three biological processes were found to be significantly overrepresented in the *SlARF9*-RNAi fruits (FDR < 0.25). These were brassinosteroid (BR) biosynthesis and degradation (bins 17.3.1 and 17.3.1.2), polyamine metabolism (22.1), and cell death (31.5; Supplementary Table S4).

## Discussion

After successful completion of pollination and fertilization, molecular, biochemical, and structural changes transform the ovary into a fruit. These changes are likely to be preceded by changes at the transcriptomic level, giving rise to a dynamic and complex regulatory network that includes signalling by phytohormones such as auxin, GA, ethylene, and abscisic acid (Lemaire-Chamley *et al.*, 2005; Vriezen *et al.*, 2008; Molesini *et al.*, 2009; Mounet *et al.*, 2012; Pascual *et al.*, 2009; Wang *et al.*, 2009).

### *Sl*ARF9 as a regulator of cell division during early tomato fruit development

In tomato, 22 putative functional *ARF* genes have been identified (Zouine *et al.*, 2014). Many of these genes show dramatic changes in expression during fruit set and throughout the different stages of fruit development, suggesting that the family of ARF-transcription factors plays an important role in the control of tomato fruit growth (Wang *et al.*, 2009; Kumar *et al.*, 2011; Wu *et al.*, 2011; Zouine *et al.*, 2014). *SlARF9* transcript levels increase within 48h after pollination and decrease again in the following days (Fig. 1A and Fig. 3) (Vriezen *et al.*, 2008; Wu *et al.*, 2011). This initial increase in *SlARF9* expression was not observed in parthenocarpic fruit formed after GA application (Vriezen *et al.*, 2008), but could be induced in unpollinated ovaries by treating with auxin (Fig. 1D). Mariotti *et al.* (2011) analysed the IAA content in ovaries before and after pollination, and found a 5-fold increase in free IAA levels in pollinated ovaries collected 2 days post anthesis compared to ovaries collected at anthesis. These levels declined 5 days post anthesis. This pattern is very similar to the expression of *SlARF9* in pollinated ovaries. Furthermore, use of the auxin-inducible DR5 promoter coupled to a fluorescent reporter gene showed that before fertilization the auxin response was mainly localized in the embryo sac and the integuments, whilst 6 days post anthesis the auxin activity was mainly observed in the funiculus and outer layer of the placental cells surrounding the seeds (Pattison and Catala, 2012). These placental cells were also stained in the 5–6mm fruit from the *pSlARF9::GUS* lines (Fig. 2A,B). It is well established that ARFs regulate gene expression in response to auxin, but the considerable overlap between auxin distribution and *SlARF9* expression indicates that the transcriptional regulation of *SlARF9* itself also depends on the auxin dynamics during tomato fruit set and development.

Normally, during the first 10–14 days of development, tomato fruit growth mainly depends on cell division (Mapelli *et al.*, 1978; Bünger-Kibler and Bangerth, 1982; Gillaspy *et al.*, 1993), but in fruits induced by the auxin IAA the period of cell division was shorter, only lasting 10 days, although cell division took place at a higher rate as compared to that in seeded control fruits. Nevertheless, these IAA-induced fruits remained smaller than control fruits because cell expansion was strongly impaired (Bünger-Kibler and Bangerth, 1982). Treatments with synthetic auxins stimulated cell division for an extended period, resulting in the formation of fruits with a higher number of pericarp cells (Bünger-Kibler and Bangerth, 1982; Serrani *et al.*, 2007). These findings suggest that during the early stages of tomato fruit development, cell division activity is tightly regulated by auxin. *Sl*ARF9 might be part of this regulatory mechanism, because decreased *SlARF9* transcript levels resulted in the formation of bigger fruits due to extra cell divisions in the pericarp, whereas increased *SlARF9* transcript levels led to the formation of smaller fruits as compared to wild type. These opposing phenotypes indicate that *Sl*ARF9 acts as a repressor of cell division during fruit growth. Although there was silencing of *SlARF9B* too, the fact that this gene is not affected by pollination, together with the phenotype of the *slarf9-1* TILLING line, indicates that *Sl*ARF9 has a major role in the process. In the fruit, *SlARF9* showed highest expression in the cell division phase (Fig. 3). The induction of *SlARF9* during this phase could create a negative feedback loop in the signal transduction pathway of auxin that promotes cell proliferation, allowing the fine-tuning of cell division activity during early fruit development.

To date, one member of the Arabidopsis *ARF* gene family, *At*ARF2 has been identified as a repressor of cell division, since the ovules of the *megaintegumenta* (*mnt*)/*arf2* mutant were increased in volume due to extra anticlinal cell divisions in the integuments, which continued for a longer period than in wild-type ovules. The expression of genes that promote cell division was not increased in young dividing tissues, but prolonged during maturation (Schruff *et al.*, 2006). In a similar way, the period of cell division might have been prolonged in the pericarp of the *SlARF9*-RNAi fruits, whilst in the *SlARF9*-OE fruits this period may have been reduced with pericarp cells starting to expand at an earlier time point than in developing wild-type fruits. To verify this hypothesis, more detailed analysis on the timing of cell division and expansion will be necessary.

### *Sl*ARF9 as a transcriptional regulator

ARF family members that contain a serine-rich MR, like *Sl*ARF9, are putative transcriptional repressors (Tiwari *et al.*, 2003), which was recently confirmed by Zouine *et al.* (2014). However, the mechanism by which ARF repressors regulate the expression of auxin-dependent genes is still unclear. Several studies suggest that their interaction with Aux/IAAs or with activating ARFs is very weak (Tiwari *et al.*, 2003; Hardtke *et al.*, 2004; Shen *et al.*, 2010). However, Rademacher *et al.* (2012) have been able to show that *At*ARF9 could interact with Aux/IAA10 in protoplasts. Alternatively, the ARF repressors may compete with the ARF activators for the AuxRE binding sites in the promoters of auxin response genes, thus inhibiting the expression of these genes independently of Aux/IAAs and providing an alternative mechanism of gene regulation (Guilfoyle and Hagen, 2007).

Despite *Sl*ARF9 being a transcriptional repressor and the obvious phenotype of the transgenic lines, no statistically significant differences in expression of single genes were found when comparing the transcriptomes of 3–4mm fruit collected from the *SlARF9*-OE and *SlARF9*-RNAi plants. This fruit size was selected as it is the developmental stage at which *SlARF9* is most highly expressed in wild-type fruits. It is, however, conceivable that it is not the stage with the highest ARF9 protein level, because many ARFs are subject to post-transcriptional regulation (Mallory *et al.*, 2005; Wang *et al.*, 2005*b*; Williams *et al.*, 2005; Wu *et al.*, 2006; Nogueira *et al.*, 2007; Finet *et al.*, 2013; Zouine *et al.*, 2014). Alternatively, it is possible that very subtle changes at the transcriptomic level already have considerable effects on early fruit growth.

GSEA is a powerful tool to help identify such subtle changes by focussing on groups of genes that are involved in similar biological pathways or processes instead of looking at the differential expression of individual genes (Thimm *et al.*, 2004). GSEA of the microarray data from *SlARF9*-OE versus *SlARF9*-RNAi fruits resulted in the identification of four functional gene categories that were overrepresented in the *SlARF9*-RNAi fruits. This included sets of genes involved in synthesis of BRs and polyamine (PA), compounds that have previously been associated with fruit development.

Pollinated ovaries from the tomato cultivar ‘Micro-Tom’, which has several mutations including one in the BR biosynthesis gene *DWARF* (*D*/*CYP85A1*), develop normally (Martí *et al.*, 2006). Also plants with a null mutation in the tomato *D* gene, extreme dwarf (*d* ^*x*^), produce fruits similar to wild type (Nomura *et al.*, 2005). Interestingly, however, high levels of brassinolide could still be detected in the fruits of this mutant, suggesting that brassinolide synthesis in the fruits might be independent of D/CYP85A1. Therefore, with a lack of a fruit phenotype in these mutants, BR cannot be dismissed as a regulator of fruit set and early fruit development. Several studies have demonstrated that the signalling pathways of BR and auxin interact at the level of transcriptional regulation (Nakamura *et al.*, 2003; Goda *et al.*, 2004; Nemhauser *et al.*, 2004). Even direct interactions have been observed between ARFs and BR signalling components (Vert *et al.*, 2008; Je and Goh, 2010; Je *et al.*, 2010; Oh *et al.*, 2014), but ARF9 has not been implicated so far.

The second biological process overrepresented in the *SlARF9*-RNAi fruits was PA synthesis. High levels of free PAs have been detected at anthesis and during the cell division stage of fruit growth in pollinated fruit, as well as in auxin- and GA-induced fruit (Egea-Cortines *et al.*, 1993; Alabadi *et al.*, 1996). PA levels are altered in the parthenocarpic transgenic *IAA9*-antisense line (Wang *et al.*, 2009) and have been associated with parthenocarpic fruit development in the *pat-2* mutant (Fos *et al.*, 2003). Furthermore, application of PAs to unpollinated tomato ovaries could induce partial parthenocarpy (Fos *et al.*, 2003), whilst application of α-difluoromethylornithine, an inhibitor of the PA biosynthesis enzyme ornithine decarboxylase, to pollinated flowers resulted in a reduction in fruit fresh weight, possibly due to a reduction in cell division (Cohen *et al.*, 1982; Teitel *et al.*, 1985). However, more work will be required to understand the mechanisms by which PAs control fruit growth.

Further studies, including one on direct targets of *Sl*ARF9, should reveal if and how these pathways mediate *Sl*ARF9 function in early fruit development.

### Modifying fruit size

The final number of cells in the pericarp is mostly determined during the cell division phase of tomato fruit development, and is an important factor in determining the size and weight of the mature fruit (Bohner and Bangerth, 1988). So far, quantitative trait loci studies have identified a number of loci for tomato fruit size and weight that have been selected during domestication (reviewed in Grandillo *et al.*, 1999; Paran and van der Knaap, 2007), but in only a few of these studies have the causative genes been cloned. One of them is *FW3.2/KLUH*, which encodes a P450 enzyme of the CYP78A subfamily. A single nucleotide polymorphism in the promoter of the gene has been associated with increased cell division and fruit weight (Chakrabarti *et al.*, 2013). Another is *FW2.2*/*CNR* (*CELL NUMBER REGULATOR*), the first fruit weight gene identified by quantitative trait loci analysis (Frary *et al.*, 2000). FW2.2 controls cell division in the early stages of tomato fruit development, possibly by interacting with a CKII kinase, which plays an important role in the signalling cascade that modulates the cell cycle (Cong and Tanksley, 2006; Liu *et al.*, 2003). Polymorphisms in members of the *FW2.2* gene family have been associated with increased fruit size during domestication across species, for example in eggplant (Doganlar *et al.*, 2002), pepper (Chaim *et al.*, 2001; van der Knaap and Tanksley, 2003), and even in un-related species such as avocado (Dahan *et al.*, 2010), maize (Guo *et al.*, 2010), and sweet and sour cherry (De Franceschi *et al.*, 2013). Thus, further elucidation of the *Sl*ARF9 signalling pathway may not just provide more insight into the regulatory mechanism by which auxin controls cell division during the early stages of fruit development in tomato, but may also be of interest to improve agronomic yield of this and other major crop species.

## Supplementary Data

Fig. S1. Wild type and transgenic fruits at breaker stage.

Fig. S2. Alignment of the predicted amino acid sequences of *Sl*ARF9 and *At*ARF9.

Fig. S3. Auxin-induced expression of *SlIAA2* and *SlIAA14*.

Fig. S4. Southern blot analysis, verifying the specificity of the *SlARF9* DNA fragment used to generate the *SlARF9*-RNAi lines.

Fig. S5. Microscopic analysis of the pericarp during early fruit development of wild-type and transgenic fruits.

Fig. S6. Principal component analysis of the normalized microarray data.

Table S1. Transcriptomic changes due to modulations in *SlARF9* expression identified by microarray analysis.

Table S2. Auxin-related *cis*-acting regulatory elements.

Table S3. *SlARF9* expression during tomato fruit set.

Table S4. Leading edge subsets from the GSEA comparing the transcriptomes of the *SlARF9*-OE and *SlARF9*-RNAi lines.

Supplementary Data

## Abbreviations:

AuxRE: auxin response elements
BR: brassinosteroid
CaMV: cauliflower mosaic virus
c-DNA-AFLP: cDNA-amplified fragment length polymorphism–based transcript profiling
CTD: C-terminal homo- and heterodimerization domains
DAP: days after pollination
FDR: false discovery rate
GA: gibberellin
GSEA: genome set enrichment analysis
GUS: β-glucuronidase
IAA: indole-3-acetic acid
kb: kilobase
MR: middle region
OE: overexpression line
PA: polyamine
PCA: principal component analysis
SED: standard error of the difference.
