# Supplementary material for: Solanum lycopersicum AUXIN RESPONSE FACTOR 9 regulates cell division activity during early tomato fruit development
Source: J Exp Bot. 2015 Apr 16;66(11):3405–16. doi: 10.1093/jxb/erv152 (PMC4449553; doi:10.1093/jxb/erv152)
Supplement: Supplementary Data [file supp_66_11_3405__index.html]

 Solanum lycopersicum AUXIN RESPONSE FACTOR 9 regulates cell division activity during early tomato fruit development — Solanum lycopersicum AUXIN RESPONSE FACTOR 9 regulates cell division activity during early tomato fruit development — Supplementary Data 

# *Solanum lycopersicum* AUXIN RESPONSE FACTOR 9 regulates cell division activity during early tomato fruit development

## Supplementary Data

Data files

**Files in this Data Supplement:**

- Supplementary Data - Supplementary Data
