# Supplementary material for: Solanum lycopersicum AUXIN RESPONSE FACTOR 9 regulates cell division activity during early tomato fruit development
Source: J Exp Bot. 2015 Apr 16;66(11):3405–16. doi: 10.1093/jxb/erv152 (PMC4449553; doi:10.1093/jxb/erv152)
Supplement: Supplementary Data [file supp_erv152_jexbot145789_file001.pdf]

***Solanum lycopersicum AUXIN RESPONSE FACTOR9* regulates cell division activity during early tomato fruit development**

Maaike de Jong, Mieke Wolters-Arts, Bernardus C.J. Schimmel, Catharina L.M. Stultiens, Peter F. M. de Groot, Stephen J. Powers, Yury M. Tikunov, Arnoud G. Bovy, Celestina Mariani, Wim H. Vriezen, Ivo Rieu

Supplementary Fig. S1. *Wild type and transgenic fruits at breaker stage*
Supplementary Fig. S2. *Alignment of the predicted amino acid sequences of* Sl*ARF9 and* At*ARF9.*
Supplementary Fig. S3. *Auxin-induced expression of* SlIAA2 *and* SlIAA14*.*
Supplementary Fig. S4. *Southern blot analysis, verifying the specificity of the* SlARF9 *DNA fragment used to generate the* SlARF9-*RNAi lines.*
Supplementary Fig. S5. *Microscopic analysis of the pericarp during early fruit development of wild type and transgenic fruits.*
Supplementary Fig. S6. *Principal component analysis of the normalized microarray data.*
Supplementary Table S1. *Transcriptomic changes due to modulations in* SlARF9 *expression identified by microarray analysis.*
Supplementary Table S2. *Auxin-related cis-acting regulatory elements.*
Supplementary Table S3. SlARF9 *expression during tomato fruit set.*
Supplementary Table S4. *Leading edge subsets from the GSEA comparing the transcriptomes of the* SlARF9-*OE and* SlARF9-*RNAi lines.*

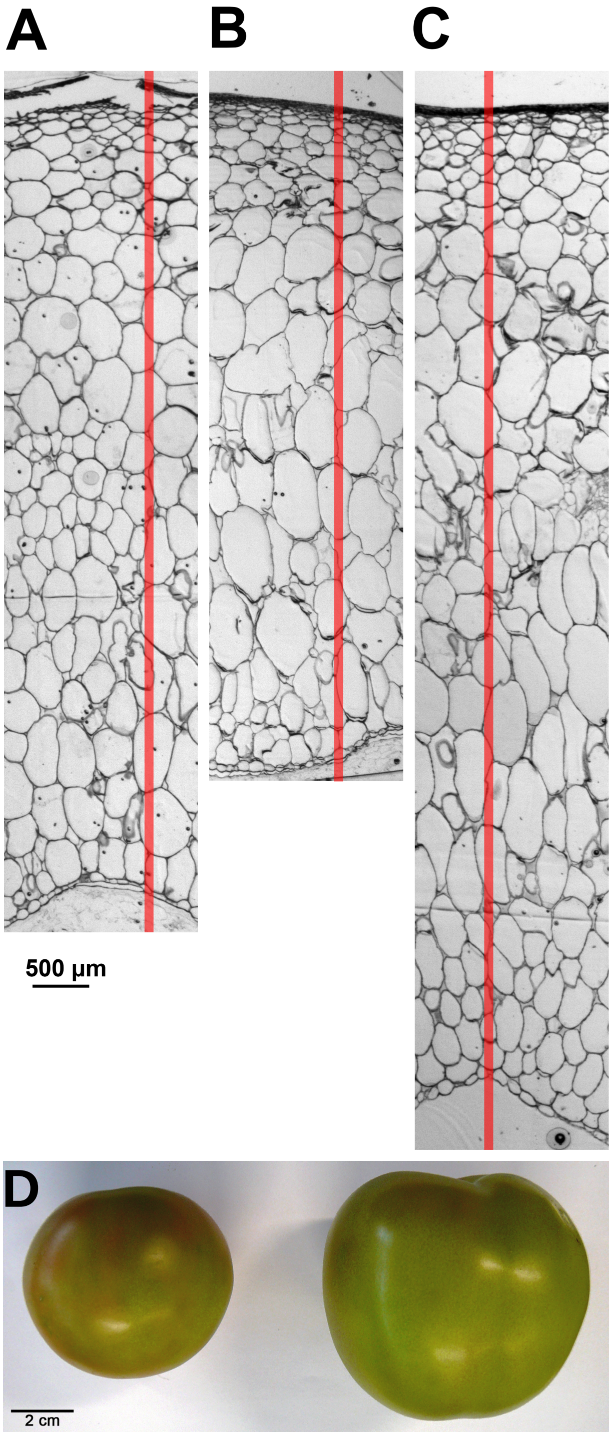

Supplementary Fig. S1. Wild type and transgenic fruits at breaker stage. Micrographs of the pericarp from wild-type (A), *SlARF9*-OE (B), and *SlARF9*-RNAi (C) fruit. For estimation of the number of cell layers within the pericarp, the number of cells along the red line were scored. (D) Picture of wild type (left) and *SlARF9*-RNAi (right) fruits.

```
SlARF9   1    MATINGWCYE SQPNMNSPGK KDALYHELWQ LCAGPVVDVP REGERVYYFP
AtARF9   1    ---------- ----MANRGG -EYLYDELWK LCAGPLVDVP QAQERVYYFP

SlARF9   51   QGHMEQLVAS INQ-EMDQRV PSFNLKSKVL CRVINSHFLA EEDNDEVYVQ
AtARF9   36   QGHMEQLEAS TQQVDLNTMK PLFVLPPKIL CNVMNVSLQA EKDTDEVYAQ

SlARF9   100  ITLMPEAPHV PEPTTPDPLI PQDVKPRFHS FCKVLTASDT STHGGFSVLR
AtARF9   86   ITLIPVGTEV DEPMSPDPSP PELQRPKVHS FSKVLTASDT STHGGFSVLR

SlARF9   150  KHANECLPPL DLNQQTPTQE LIAKDLHDVE WRFKHIFRGQ PRRHLLTTGW
AtARF9   136  KHATECLPPL DMTQQTPTQE LVAEDVHGYQ WKFKHIFRGQ PRRHLLTTGW

SlARF9   200  STFVSSKKLV AGDSFVFLRG NNGQLRVGVK RLVRQQSSMP SSVMSSQSMH
AtARF9   186  STFVTSKRLV AGDTFVFLRG ENGELRVGVR RANLQQSSMP SSVISSHSMH

SlARF9   250  LGVLATASHA VTTQTMFVVY YKPRTTQFIV GVNKYLEALK HEYAVGMRFK
AtARF9   236  LGVLATARHA TQTKTMFIVY YKPRTSQFII SLNKYLEAMS NKFSVGMRFK

SlARF9   300  MQFEAEGNPD RRFMGTIVGI DDLSSQWKNS AWRSLKVRWD EPAAIARPDR
AtARF9   286  MRFEGEDSPE RRYSGTVIGV KDCSPHWKDS KWRCLEVHWD EPASISRPNK

SlARF9   350  VSPWEIKPYV CSIPNVLVPP TAEKNKRHRL HSEIKISEQP SSSNASAVWN
AtARF9   336  VSPWEIEPFV NSEN--VPKS VMLKNKRPRQ VSEVSALDVG --ITASNLWS

SlARF9   400  PSLRSPQFNT FGINSSTNCA LASLTESGWQ LPHLNTSGML VDEPEDGRSA
AtARF9   382  SVLTQP--HE FAQSCIT--- ------SQWS SPQQCHR--- -DANEDAKKS

SlARF9   450  PTWCGFPCVL APQFGQGTNQ PIVIPTDGRK CDTKKTCRLF GIDLKSSSIS
AtARF9   417  DWLNNSYSVS NVAKDSTLND QMVSPVEQKK PETTANYRLF GIDLMSSSLA

SlARF9   500  TTEAR-LQLQ PAGISCVFAE RAPPNTVPAG DSDQKSELSV DFKDQMQGHL
AtARF9   467  VPEEKTAPMR PINIS----- ----KPTMDS HSDPKSEISK VSEEKKQEPA

SlARF9   549  RLPLKEVQSK QSCSTRSRTK VQMQGVAVGR AVDLTILKGY DELTKELEEM
AtARF9   508  EGSPKEVQSK QSSSTRSRTK VQMQGVPVGR AVDLNALKGY NELIDDIEKL

SlARF9   599  FEIQGELQSR QKWGILFTDD EGDTMLMGDY PWQDFCNVVR KIFICSSQDM
AtARF9   558  FDIKGELRSR NQWEIVFTDD EGDMMLVGDD PWPEFCNMVK RIFIWSKEEV

SlARF9   649  KKLT------ ---------- --LSRADS-- -
AtARF9   608  KKMTPGNQLR MLLREVETTL TTTSKTDNHS N
```

Supplementary Fig. S2. Alignment of the predicted amino acid sequences of *Sl*ARF9 and *At*ARF9. The numbers on the left indicate the position of the amino acid residues relative to the putative translational start site. The DNA binding domain is underlined with a dashed line and the conserved domains III and IV are indicated by a thick line and thick dashed line. Identical and similar amino acids are shaded in black and grey, respectively.

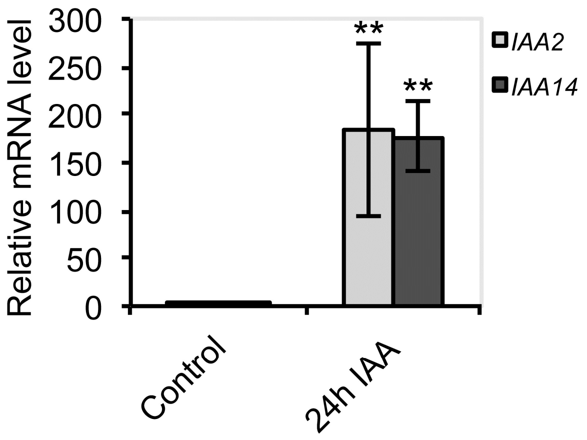

Supplementary Fig. S3. Auxin-induced expression of *SlIAA2* and *SlIAA14*. Relative mRNA levels of *SlIAA2* and *SlIAA14* in tomato ovaries of emasculated flowers collected 24h after auxin treatment (IAA). Untreated ovaries were used as a control. 4-5 ovaries were pooled for each sample. Standard errors are indicated (n=2). **, significantly different from the control, *P*<0.01.

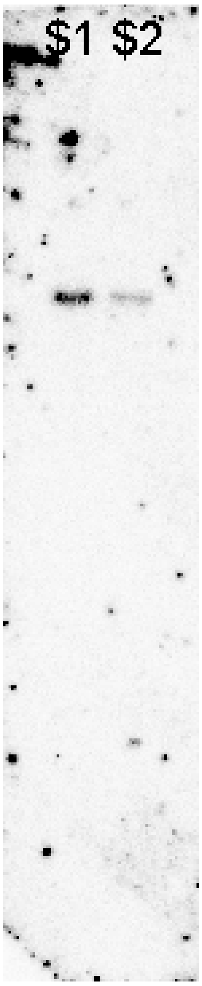

Supplementary Fig. S4. Southern blot analysis, verifying the specificity of the *SlARF9* DNA fragment used to generate the *SlARF9*-RNAi lines. To generate the *SlARF9*-RNAi lines, a DNA fragment encoding the mid region of the ARF (aa 367-506) was used. The specificity of this fragment was tested by southern blot analysis using the genomic DNA of two wild-type plants. Both samples showed one band after hybridization with the *SlARF9*-RNAi mid region probe.

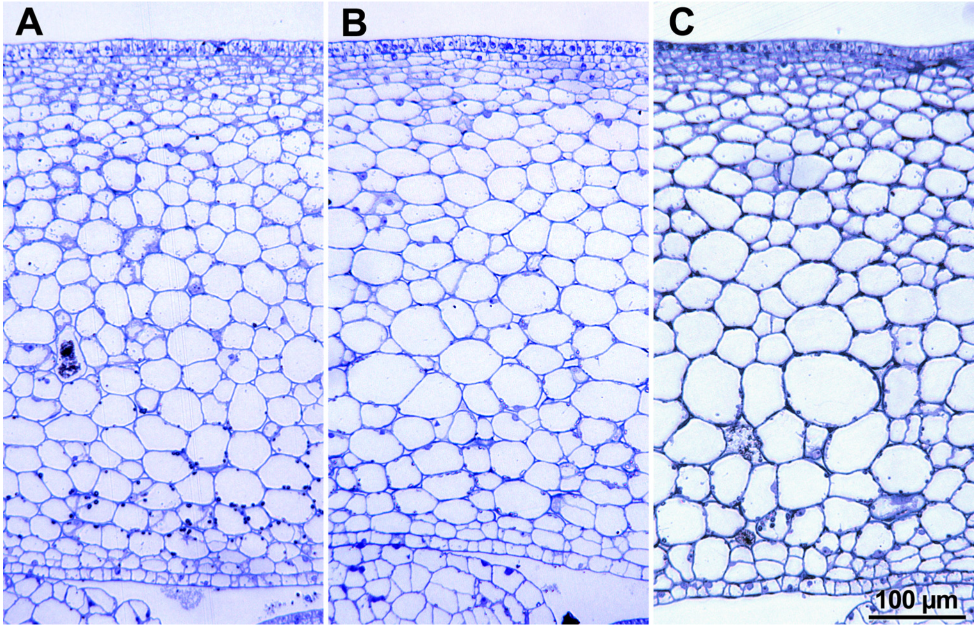

Supplementary Fig. S5. Microscopic analysis of the pericarp during early fruit development of wild-type and transgenic fruits. Micrographs of *SlARF9*-RNAi (A), wild-type (B), and *SlARF9*-OE (C) fruit, 7-8 mm in diameter. At this stage of fruit development, the cells in the mesocarp and endocarp are already bigger in *SlARF9*-OE fruit compared to wild-type fruit, whilst the cells in the *SlARF9*-RNAi fruit are smaller and have a higher number of cell layers.

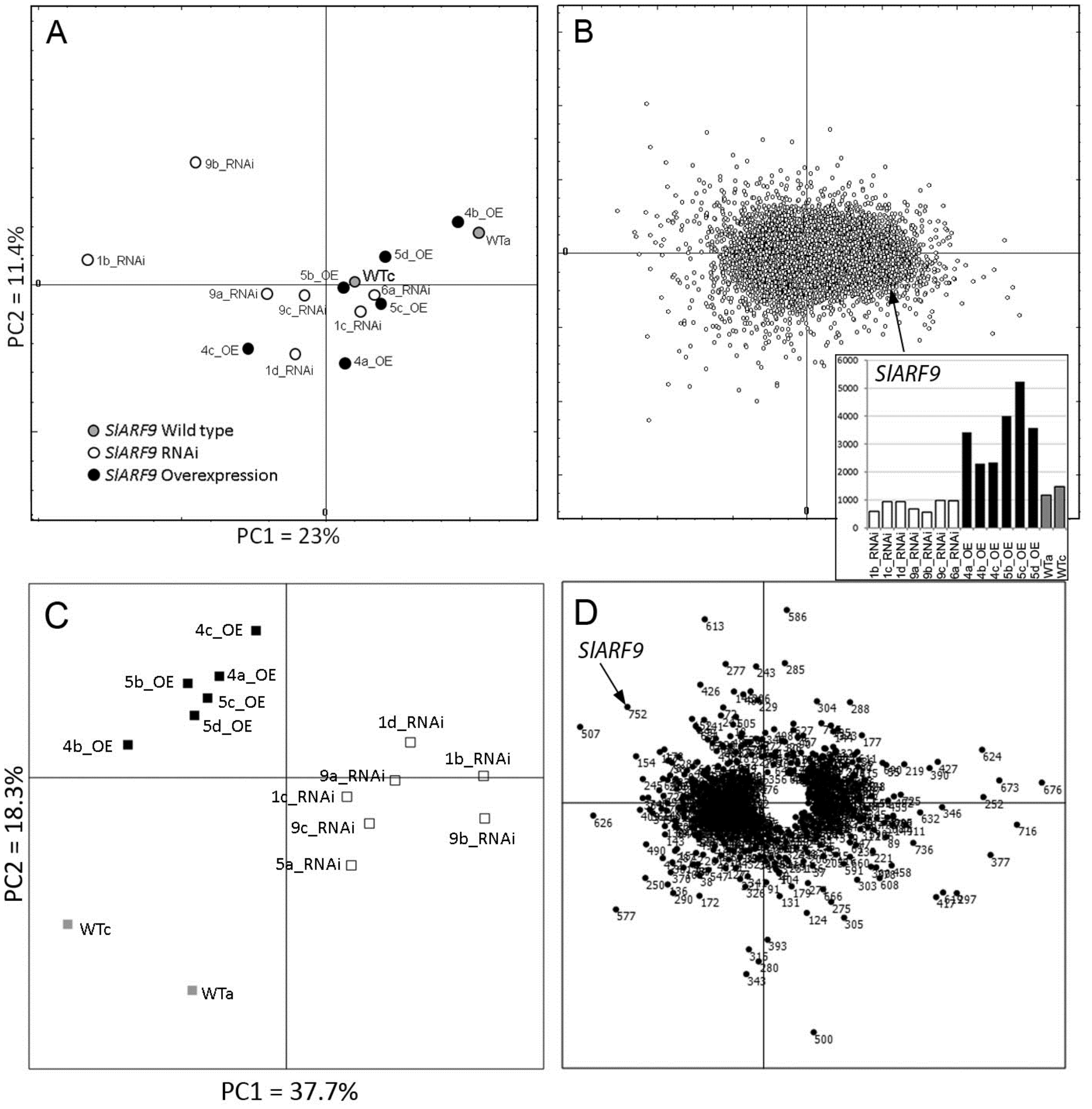

Supplementary Fig. S6. Principal Components Analysis (PCA) of the normalized log-transformed microarray data. (A) PCA score plot based on the expression of 19,168 genes (filtered for a 2-fold change between any two samples, intensity cut off value = 20) in the pericarp of 3-4mm fruits collected from *SlARF9*-OE (OE, black), *SlARF9*-RNAi (RNAi, white) and wild-type (WT, grey) plants. The loading plot is represented in (B). The arrow indicates the position of *SlARF9*. The insert shows the expression of *SlARF9* in each individual sample. (C) PCA score plot based on those genes with significantly different expression levels (t-test, P<0.05) between any two types of plants: wild type, *SlARF9*-OE, and *SlARF9*-RNAi, respectively. The loading plot is represented in (D). The genes are numbered; corresponding annotations and loading values in the first two principal components can be found in Supplementary Table S1.

Supplementary Table S1. *Transcriptomic changes due to modulations in* SlARF9 *expression identified by microarray analysis*

Genes whose transcript levels significantly changed (t-test, *P*<0.05) when comparing the transcriptomes of any two types of plants; wild type (WT), *SlARF9*-OE and *SlARF9*-RNAi. Genes are sorted according to their loading values of PC1 in the Principal Components Analysis, which describes the difference between wild type and *SlARF9*-OE plants on one hand and *SlARF9*-RNAi plants on the other (Supplementary Fig. S5D). For each gene, the fold change in expression and correlation to *SlARF9* transcript levels are shown.

ID	ITAG2.3 Hit description	MapMan Bin Code	PCA loadings			OE vs. WT		OE vs. RNAi		RNAi vs. WT		Pearson correlation to *SlARF9*	
			PCA label	PC1	PC2	Fold change	*P*-value	Fold change	*P*-value	Fold change	*P*-value	r	*P*-value
Solyc02g064830	Indole-3-acetic acid-amido synthetase GH3.8	17.2.3	676	-0.15	-0.01	2.26	0.497	-4.57	0.019	10.30	0.035	-0.45	0.092
Solyc03g119590	NIMIN2c protein	35.2	716	-0.13	0.033	1.29	0.754	-3.24	0.006	4.18	0.032	-0.38	0.162
Solyc09g015770	WRKY transcription factor 6	27.3.32	673	-0.12	-0.01	2.78	0.483	-2.52	0.024	6.98	0.019	-0.28	0.312
Solyc08g067540	Non-specific lipid-transfer protein	35.2	377	-0.12	0.057	-1.44	0.276	-5.17	0.000	3.58	0.035	-0.64	0.01
Solyc03g116890	WRKY transcription factor 2	27.3.32	624	-0.12	-0.04	2.19	0.047	-3.84	0.061	8.41	0.072	-0.44	0.101
Solyc09g083360	Basic helix-loop-helix protein	27.3.6	252	-0.12	0.005	1.08	0.759	-3.98	0.007	4.29	0.077	-0.52	0.047
Solyc05g007980	High affinity sulfate transporter 2	34.6	507	0.107	-0.07	1.31	0.228	3.76	0.000	-2.87	0.078	0.72	0.002
Solyc04g071990	Tomato GIGANTEA 1	33.99	626	0.101	0.011	-1.35	0.567	2.24	0.034	-3.03	0.083	0.18	0.521
Solyc01g016840	Unknown Protein	35.2	297	-0.1	0.09	-4.64	0.163	-5.09	0.000	1.10	0.538	-0.47	0.077
Solyc01g057980	HR7 protein	35.2	346	-0.09	0.012	1.12	0.828	-3.01	0.017	3.37	0.096	-0.41	0.129
Solyc11g021060	Unknown Protein	35.2	619	-0.09	0.089	-1.61	0.442	-3.36	0.010	2.09	0.224	-0.45	0.092
Solyc01g056310	Laccase-2	26.7	577	0.09	0.096	-2.38	0.143	2.63	0.297	-6.27	0.007	-0.03	0.915
Solyc08g062330	Ankyrin repeat protein	31.1	427	-0.09	-0.03	1.59	0.397	-2.75	0.036	4.37	0.066	-0.44	0.101
Solyc08g077760	Unknown Protein	35.2	417	-0.09	0.096	-2.89	0.076	-4.79	0.003	1.65	0.567	-0.62	0.014
Solyc07g006480	LRR receptor-like serine/threonine-protein kinase FEI 1	30.2.11	390	-0.09	-0.02	1.37	0.547	-3.63	0.033	4.97	0.108	-0.33	0.23
Solyc08g082630	Auxin response factor 9	27.3.4	752	0.08	-0.09	2.63	0.009	4.27	0.000	-1.63	0.032	1.00	0
Solyc06g060110	Amino acid permease	34.3	632	-0.08	0.017	-1.02	0.819	-2.70	0.011	2.66	0.120	-0.54	0.038
Solyc03g096740	Unknown Protein	35.2	154	0.076	-0.04	1.17	0.422	2.52	0.001	-2.16	0.262	0.56	0.03
Solyc12g009220	Jasmonate ZIM-domain protein 1	17.7.2	736	-0.08	0.044	-1.05	0.664	-2.48	0.010	2.36	0.100	-0.46	0.084
Solyc12g098610	Xyloglucan endotransglucosylase/hydrolase 8	10.7	409	0.075	0.007	-1.45	0.339	2.36	0.003	-3.43	0.001	0.43	0.11
Solyc03g093860	ATP-dependent DNA helicase	28.1	364	0.074	-0	-1.05	0.894	1.94	0.012	-2.04	0.111	0.48	0.07
Solyc02g064960	AP2-like ethylene-responsive transcription factor	27.3.3	245	0.073	-0.02	1.30	0.728	2.40	0.012	-1.85	0.083	0.56	0.03
Solyc01g066360	Pectinesterase	10.8.1	250	0.073	0.068	-2.44	0.098	2.33	0.118	-5.68	0.000	-0.02	0.944
Solyc03g115770	Timing of CAB expression-like	27.3.66	490	0.072	0.038	-1.46	0.329	2.33	0.069	-3.39	0.001	0.19	0.498

Supplementary Table S1. continued

Solyc12g042930	Os01g0611000 protein	35.2	411	-0.07	0.028	-1.20	0.434	-2.70	0.015	2.26	0.232	-0.40	0.14
Solyc07g053550	Glutaredoxin	21.4	219	-0.07	-0.03	1.51	0.337	-1.75	0.025	2.64	0.022	-0.45	0.092
Solyc07g017530	Conserved oligomeric Golgi complex subunit 3	29.3.4.2	366	0.07	0.008	-1.26	0.233	1.68	0.041	-2.12	0.098	0.51	0.052
Solyc06g007610	Early tobacco anther 1	35.2	740	0.07	-0	-1.20	0.528	2.27	0.021	-2.71	0.027	0.55	0.034
Solyc02g090350	Cytochrome P450	26.10	725	-0.07	0.001	1.22	0.751	-2.04	0.014	2.50	0.033	-0.46	0.084
Solyc12g055810	AAA-ATPase	29.5.9	349	0.068	0.01	-1.44	0.464	1.99	0.004	-2.86	0.016	0.27	0.33
Solyc04g009440	NAC domain protein	33.99	452	-0.07	0.001	1.37	0.174	-2.07	0.003	2.85	0.012	-0.51	0.052
Solyc02g070720	Unknown Protein	35.2	149	-0.07	0.025	1.07	0.772	-2.71	0.022	2.91	0.133	-0.49	0.064
Solyc04g082500	ATP binding / serine-threonine kinase	29.4.1.57	385	-0.06	0.019	-1.26	0.355	-2.37	0.013	1.88	0.260	-0.59	0.021
Solyc01g108780	Alpha-hydroxynitrile lyase	26.8	702	-0.06	0.021	-1.12	0.822	-1.84	0.006	1.64	0.084	-0.30	0.277
Solyc09g074230	Glucose transporter 8	34.2	672	0.064	-0.01	1.03	0.932	1.96	0.010	-1.91	0.106	0.44	0.101
Solyc05g053860	Solute carrier family 22 member 5	34.14	157	0.064	-0.04	-1.13	0.743	2.48	0.005	-2.80	0.025	0.62	0.014
Solyc03g058490	Unknown Protein	35.2	148	0.063	0.007	-1.09	0.718	1.90	0.029	-2.07	0.098	0.47	0.077
Solyc09g092520	Xyloglucan endotransglucosylase/hydrolase 8	10.7	492	0.063	0.051	-2.41	0.072	1.55	0.153	-3.73	0.005	0.03	0.915
Solyc03g026160	F-box family protein	31.1	458	-0.06	0.064	-1.74	0.383	-2.43	0.002	1.40	0.281	-0.47	0.077
Solyc03g083960	Trehalose 6-phosphate phosphatase	3.2.2	648	0.063	-0.02	-1.15	0.672	1.91	0.022	-2.20	0.084	0.52	0.047
Solyc09g010060	Kinesin	31.1	691	0.062	-0.01	-1.25	0.668	1.77	0.005	-2.21	0.058	0.39	0.151
Solyc03g083150	Unknown Protein	35.2	143	0.062	0.03	-1.50	0.178	1.64	0.050	-2.46	0.015	0.43	0.11
Solyc01g107490	BHLH transcription factor	17.5.3	455	-0.06	0.005	-1.00	0.921	-1.96	0.008	1.95	0.057	-0.64	0.01
Solyc02g084080	Unknown Protein	35.2	55	-0.06	-0.03	2.34	0.018	-1.95	0.082	4.57	0.022	-0.27	0.33
Solyc07g065880	Kinesin	31.1	130	0.062	0.022	-1.68	0.057	1.74	0.014	-2.93	0.006	0.25	0.369
Solyc10g009550	WRKY transcription factor	27.3.32	679	-0.06	0.021	-1.13	0.764	-2.17	0.007	1.92	0.136	-0.45	0.092
Solyc10g055470	Beta-galactosidase	26.3.2	136	0.061	0.075	-2.43	0.071	1.63	0.403	-3.96	0.000	-0.12	0.67
Solyc05g051440	Plant-specific domain TIGR01570 family protein	35.2	89	-0.06	0.037	-1.05	0.755	-2.85	0.002	2.71	0.071	-0.50	0.058
Solyc02g092630	Unknown Protein	35.2	189	0.061	0.009	-1.10	0.680	1.98	0.022	-2.18	0.027	0.67	0.006
Solyc01g087790	Subtilisin-like protease	29.5.1	178	0.061	-0.05	1.13	0.510	1.96	0.011	-1.74	0.242	0.60	0.018
Solyc07g048110	Uncharacterized membrane protein C24H6.13	20.2.3	700	0.06	0.002	-1.26	0.386	1.59	0.026	-2.00	0.094	0.34	0.215
Solyc09g008970	Unknown Protein	35.2	308	-0.06	0.025	-1.24	0.388	-2.12	0.006	1.71	0.170	-0.64	0.01
Solyc03g026210	Dihydrodipicolinate reductase family protein	13.1.3.5.2	217	0.06	-0.02	1.15	0.776	1.95	0.020	-1.69	0.159	0.73	0.002
Solyc08g013750	F-box family protein	29.5.11.4.3.2	370	0.06	0.065	-2.31	0.043	1.30	0.350	-3.02	0.009	0.00	1
Solyc04g005040	Matrix metalloproteinase	29.5.7	690	-0.06	-0.03	1.65	0.240	-1.59	0.091	2.61	0.037	-0.32	0.245
Solyc07g055870	Kinase family protein	29.4	301	0.059	0.056	-2.30	0.001	1.25	0.185	-2.87	0.023	0.00	1
Solyc12g010800	BZIP transcription factor family protein	27.3.35	78	0.059	-0.04	1.58	0.301	2.18	0.011	-1.38	0.358	0.58	0.023
Solyc01g005510	Laccase-2	26.7	290	0.058	0.082	-2.25	0.143	1.97	0.428	-4.42	0.002	-0.06	0.832
Solyc11g010120	Peroxidase 17	26.12	532	0.058	-0.02	1.31	0.395	2.01	0.004	-1.53	0.149	0.56	0.03
Solyc09g018780	Oligoribonuclease	28.99	58	0.057	-0.03	1.34	0.347	2.10	0.006	-1.57	0.216	0.71	0.003
Solyc09g010320	Predicted nucleoside-diphosphate-sugar epimerase	35.2	287	0.056	0.044	-2.11	0.099	1.58	0.149	-3.33	0.029	0.00	1
Solyc03g123590	Remorin family protein	27.3.99	704	0.056	0.017	-1.38	0.319	1.83	0.013	-2.52	0.002	0.48	0.07
Solyc03g065250	CER1	16.7	697	0.056	0.022	-1.58	0.109	1.91	0.007	-3.03	0.011	0.41	0.129
Solyc06g083100	HAUS augmin-like complex subunit 3	35.2	536	0.056	0.02	-1.42	0.187	1.58	0.025	-2.25	0.015	0.39	0.151
Solyc11g042480	Unknown Protein	35.2	151	0.056	0.044	-1.95	0.060	1.36	0.219	-2.65	0.013	0.00	1

Supplementary Table S1. continued

Solyc04g054740	Inositol-3-phosphate synthase	3.4.3	608	-0.06	0.075	-2.66	0.083	-2.27	0.004	-1.18	0.787	-0.65	0.009
Solyc11g017270	Receptor like kinase, RLK	30.2.11	475	-0.06	0.021	-1.21	0.464	-2.26	0.006	1.87	0.146	-0.62	0.014
Solyc11g042880	Harpin-induced protein	35.2	667	0.055	-0.02	-1.11	0.590	1.76	0.003	-1.95	0.059	0.56	0.03
Solyc06g062430	Inositol oxygenase	3.4.4	644	0.055	0.023	-1.55	0.088	1.58	0.029	-2.45	0.027	0.21	0.453
Solyc10g005110	Coproporphyrinogen III oxidase aerobic	19.8	284	0.055	-0.01	1.01	0.901	1.87	0.017	-1.86	0.108	0.51	0.052
Solyc07g053810	Squamosa promoter binding-like protein	27.3.28	378	-0.05	0.065	-1.51	0.157	-3.04	0.009	2.01	0.344	-0.40	0.14
Solyc06g060450	Transmembrane emp24 domain-containing protein 10	34.99	186	-0.05	0.031	-1.17	0.634	-2.32	0.003	1.98	0.047	-0.61	0.016
Solyc01g098230	Mitochondrial import inner membrane translocase	29.3.2	470	0.054	0.006	-1.16	0.651	1.72	0.017	-2.00	0.049	0.40	0.14
Solyc08g074960	Nodulin-like family protein expressed	33.99	133	0.054	0.008	-1.60	0.135	1.69	0.015	-2.71	0.003	0.21	0.453
Solyc04g080170	Protein serine/threonine kinase	29.4	587	0.053	-0.02	-1.02	0.949	1.72	0.008	-1.76	0.105	0.46	0.084
Solyc10g086280	Heavy metal-associated domain containing protein expressed	34.99	656	-0.05	0.001	1.10	0.707	-1.70	0.014	1.86	0.055	-0.51	0.052
Solyc00g009110	Inositol-1 4 5-trisphosphate 5-Phosphatase-like protein	28.1	100	0.053	0.061	-2.36	0.031	1.36	0.204	-3.19	0.007	-0.02	0.944
Solyc01g111610	Protein binding protein	29.5.11.4.2	549	-0.05	0.001	1.07	0.842	-1.69	0.007	1.80	0.037	-0.60	0.018
Solyc01g105660	1-aminocyclopropane-1-carboxylate oxidase	17.5.1	286	0.052	-0.04	1.53	0.402	2.55	0.005	-1.67	0.130	0.77	0.001
Solyc10g085870	UDP-glucosyltransferase family 1 protein	26.2	221	-0.05	0.052	-1.91	0.238	-2.21	0.000	1.16	0.353	-0.64	0.01
Solyc09g089870	Transcription factor	27.3.6	239	0.052	-0	-1.21	0.567	1.60	0.021	-1.95	0.055	0.16	0.569
Solyc10g009370	Mutator-like transposase	27.3.99	300	-0.05	0.064	-1.83	0.229	-2.09	0.010	1.15	0.526	-0.49	0.064
Solyc07g065600	Genomic DNA chromosome 5 P1 clone MDF20	35.2	443	0.051	0.013	-1.60	0.107	1.49	0.021	-2.39	0.031	0.13	0.644
Solyc02g071830	AT5g06970/MOJ9_14	35.2	201	0.051	-0	-1.00	0.918	1.74	0.008	-1.74	0.106	0.42	0.119
Solyc08g082680	Ring H2 finger protein	27.3.99	230	0.051	-0	-1.41	0.444	1.76	0.002	-2.48	0.027	0.20	0.475
Solyc09g008000	ABC transporter G family member 28	34.16	12	0.051	0.016	-1.85	0.061	1.57	0.043	-2.89	0.005	0.17	0.545
Solyc03g111430	Unknown Protein	35.2	270	0.051	0.008	-1.44	0.120	1.59	0.023	-2.28	0.015	0.48	0.07
Solyc07g053210	DNA cross-link repair 1B-like protein	28.2	187	0.051	-0	-1.06	0.872	1.66	0.009	-1.76	0.091	0.50	0.058
Solyc09g082830	ARGONAUTE 1	27.3.36	489	0.051	0.003	-1.21	0.680	1.76	0.006	-2.13	0.024	0.48	0.07
Solyc04g058090	Phosphoribosylformylglycinamidine cyclo-ligase	23.1.2.5	405	0.05	-0	-1.11	0.725	1.65	0.010	-1.84	0.077	0.47	0.077
Solyc09g075400	Unknown Protein	35.2	590	-0.05	0.029	1.06	0.877	-1.72	0.015	1.82	0.042	-0.25	0.369
Solyc06g072740	Inositol-tetrakisphosphate 1-kinase 1	30.4.5	508	-0.05	-0.01	1.51	0.243	-1.44	0.091	2.18	0.029	-0.18	0.521
Solyc04g009960	L-allo-threonine aldolase	13.2.5.2	345	-0.05	0.011	-1.02	0.842	-1.79	0.004	1.76	0.034	-0.70	0.004
Solyc01g087970	Serine carboxypeptidase 1	29.5.5	400	0.05	-0.02	-1.08	0.939	1.74	0.009	-1.88	0.170	0.42	0.119
Solyc08g076860	Zinc-binding family protein	27.3.99	371	-0.05	-0.01	1.19	0.321	-1.55	0.033	1.85	0.090	-0.45	0.092
Solyc06g074320	BZIP transcription factor	27.3.35	177	-0.05	-0.05	2.48	0.005	-1.28	0.192	3.16	0.004	-0.06	0.832
Solyc11g071810	CRABS CLAW	27.3.10	394	0.048	-0	1.03	0.967	1.64	0.012	-1.60	0.103	0.54	0.038
Solyc02g071700	GDSL esterase/lipase At1g29670	26.28	687	-0.05	0.021	-1.14	0.911	-1.71	0.004	1.50	0.116	-0.39	0.151
Solyc07g064340	Serine/threonine-protein kinase	29.4.1.57	92	0.048	-0.03	1.12	0.814	2.02	0.015	-1.81	0.141	0.48	0.07
Solyc07g052320	Beta-1 3-galactosyltransferase 6	29.7	307	-0.05	-0.01	1.36	0.207	-1.52	0.004	2.07	0.014	-0.39	0.151
Solyc07g042520	Sucrose synthase 4	2.2.1.5	237	0.047	0.058	-2.05	0.042	1.21	0.376	-2.48	0.010	0.07	0.804
Solyc09g072860	Genomic DNA chromosome 5 P1 clone MRH10	35.2	480	0.047	0.024	-1.49	0.032	1.32	0.112	-1.96	0.066	0.22	0.431
Solyc02g076950	UPF0052 domain protein	35.2	200	0.047	0.026	-1.49	0.155	1.42	0.097	-2.11	0.033	0.27	0.33

Supplementary Table S1. continued

Solyc08g082140	Esterase/lipase/thioesterase family protein	35.2	11	0.047	0.019	-1.72	0.214	1.56	0.109	-2.69	0.019	0.06	0.832
Solyc02g091670	Octicosapeptide/Phox/Bem1p domain-containing protein	29.4	628	0.047	-0	-1.14	0.431	1.60	0.014	-1.83	0.031	0.44	0.101
Solyc01g109630	mTERF domain-containing protein 3, mitochondrial	27.3.99	363	0.047	0.008	-1.43	0.005	1.52	0.016	-2.17	0.025	0.29	0.294
Solyc04g072920	Trehalose-6-phosphate phosphatase	3.2.2	82	-0.05	-0.03	1.78	0.066	-1.28	0.109	2.28	0.020	-0.29	0.294
Solyc08g005680	Undecaprenyl pyrophosphate synthase	16.1.2	1	-0.05	0	1.22	0.389	-1.65	0.044	2.01	0.085	-0.36	0.187
Solyc09g056060	Unknown Protein	35.2	34	-0.05	0.022	-1.14	0.529	-2.01	0.013	1.76	0.213	-0.46	0.084
Solyc01g050010	HAT family dimerisation domain containing protein	28.1.1.4	94	0.046	0.058	-3.01	0.023	1.13	0.462	-3.39	0.018	-0.06	0.832
Solyc10g079930	UDP-glucosyltransferase HvUGT5876	26.2	184	-0.05	0.004	1.07	0.759	-1.73	0.002	1.85	0.022	-0.54	0.038
Solyc01g099560	Carbonyl reductase 3	26.22	611	-0.05	-0.04	1.98	0.018	-1.36	0.085	2.69	0.004	-0.29	0.294
Solyc06g060710	Glycogenin 2	2.1.2	102	0.046	-0.02	-1.07	0.956	1.81	0.002	-1.93	0.048	0.31	0.261
Solyc12g013960	Unknown Protein	35.2	317	-0.05	0.03	-1.21	0.629	-1.80	0.002	1.49	0.117	-0.54	0.038
Solyc07g066340	UPF0431 protein C1orf66 homolog	35.2	506	0.046	0.007	-1.23	0.384	1.50	0.010	-1.84	0.032	0.47	0.077
Solyc03g118810	Calmodulin	30.3	615	-0.05	-0.03	1.56	0.172	-1.41	0.121	2.19	0.036	-0.34	0.215
Solyc07g044740	3-hydroxyisobutyryl-CoA hydrolase-like protein 5	11.9.4.3	45	0.046	-0.02	1.18	0.532	1.62	0.020	-1.38	0.252	0.45	0.092
Solyc11g064770	Cc-nbs-lrr, resistance protein	20.1	436	0.046	0.017	-1.36	0.126	1.38	0.074	-1.88	0.041	0.33	0.23
Solyc08g067370	Unknown Protein	35.2	732	0.046	-0.01	-1.10	0.762	1.53	0.018	-1.69	0.107	0.57	0.027
Solyc01g088720	Transcription initiation factor IIB	27.2	523	0.046	0.032	-1.69	0.030	1.26	0.180	-2.13	0.022	0.12	0.67
Solyc01g109250	TMV response-related protein	35.2	238	-0.05	0.003	1.09	0.672	-1.60	0.010	1.74	0.056	-0.54	0.038
Solyc07g063450	Amine oxidase family protein	26.7	474	0.045	0.032	-1.50	0.141	1.34	0.166	-2.01	0.029	0.13	0.644
Solyc07g041340	ELF4-like protein	35.2	190	0.045	-0.01	1.06	0.725	1.70	0.003	-1.60	0.073	0.57	0.027
Solyc02g087950	cDNA clone J023065D24 full insert sequence	29.2.1.2.2.34	425	-0.05	-0.01	1.46	0.113	-1.75	0.012	2.54	0.014	-0.40	0.14
Solyc06g060670	Protein arginine N-methyltransferase	26.6	381	0.045	0.02	-1.37	0.159	1.44	0.042	-1.97	0.020	0.46	0.084
Solyc11g007010	Proline-, glutamic acid- and leucine-rich protein 1	35.2	664	0.045	0.024	-1.46	0.268	1.43	0.101	-2.09	0.019	0.16	0.569
Solyc03g033730	BZIP transcription factor	27.3.35	2	0.045	0.048	-2.53	0.000	1.28	0.006	-3.23	0.000	-0.03	0.915
Solyc10g085820	DnaJ-like subfamily C member 28 conserved domain protein	35.2	236	-0.05	-0.04	2.23	0.019	-1.24	0.329	2.78	0.011	-0.05	0.86
Solyc09g075060	Beta-glucosidase	26.3	44	0.045	-0.03	1.63	0.122	2.04	0.007	-1.25	0.399	0.58	0.023
Solyc02g078150	Plant-specific domain TIGR01615 family protein	35.2	152	0.045	-0.07	1.57	0.087	2.04	0.004	-1.30	0.513	0.71	0.003
Solyc11g066950	Stress responsive A/B barrel domain family protein	35.2	368	-0.05	-0.01	1.37	0.069	-1.75	0.012	2.39	0.031	-0.47	0.077
Solyc02g011980	50S ribosomal protein L23	35.2	172	0.044	0.087	-3.43	0.025	-1.11	0.702	-3.09	0.037	-0.27	0.33
Solyc12g006790	ARGONAUTE 1	27.3.36	176	-0.04	-0.02	1.89	0.073	-1.51	0.048	2.85	0.004	-0.27	0.33
Solyc02g088920	Ras GTPase-activating protein-binding protein 2	29.3.1	160	0.044	-0.02	1.15	0.635	1.87	0.001	-1.62	0.010	0.62	0.014
Solyc09g074650	Peptide chain release factor 1	29.2.5	264	0.044	-0.01	1.07	0.556	1.61	0.009	-1.50	0.165	0.66	0.007
Solyc10g008470	Engulfment and cell motility protein 1	35.2	171	0.044	-0.01	-1.24	0.504	1.71	0.032	-2.12	0.103	0.24	0.389
Solyc06g084480	Serine protease	29.5.5	361	0.044	0.016	-1.30	0.318	1.54	0.037	-2.00	0.002	0.34	0.215
Solyc10g017620	5-dehydro-2-deoxygluconokinase 1	2.2.1.1	3	0.044	0.057	-2.28	0.047	1.39	0.246	-3.16	0.001	0.02	0.944
Solyc03g005320	Fatty acid elongase 3-ketoacyl-CoA synthase	11.1.10	296	0.044	0.011	-1.33	0.167	1.56	0.023	-2.08	0.019	0.37	0.175
Solyc02g063360	C2 domain-containing protein	27.3.11	54	-0.04	-0	1.04	0.902	-2.01	0.027	2.08	0.162	-0.49	0.064

Supplementary Table S1. continued

Solyc09g098250	Trithorax-like protein histone-lysine N-methyltransferase	27.3.69	493	0.044	0.035	-1.69	0.056	1.21	0.311	-2.04	0.029	0.16	0.569
Solyc08g048410	Ubiquitin carboxyl-terminal hydrolase	29.5.11.5	38	0.044	0.068	-3.25	0.018	1.11	0.714	-3.60	0.007	-0.05	0.86
Solyc08g062100	Unknown Protein	35.2	369	-0.04	-0.03	1.79	0.090	-1.34	0.117	2.41	0.006	-0.24	0.389
Solyc08g007890	Mitochondrial aldehyde dehydrogenase	35.2	120	0.043	0.018	-1.51	0.128	1.35	0.061	-2.04	0.027	0.20	0.475
Solyc09g072720	AT1G10385-like protein	35.2	303	-0.04	0.076	-1.80	0.106	-2.60	0.015	1.45	0.612	-0.44	0.101
Solyc12g098230	Unknown Protein	35.2	693	-0.04	-0.02	1.60	0.028	-1.51	0.002	2.42	0.001	-0.46	0.084
Solyc03g121880	Protein phosphatase 2C	29.4	233	-0.04	0.045	-1.31	0.411	-1.90	0.005	1.45	0.173	-0.62	0.014
Solyc03g112130	AT5G28150-like protein	35.2	499	0.043	-0.07	1.60	0.421	2.33	0.018	-1.45	0.266	0.70	0.004
Solyc12g035670	MCM3-associated protein	35.2	566	0.043	0.031	-1.55	0.092	1.35	0.109	-2.08	0.011	0.30	0.277
Solyc03g063090	Unknown Protein	35.2	488	0.043	-0	-1.18	0.646	1.56	0.023	-1.84	0.075	0.34	0.215
Solyc06g071930	Unknown Protein	35.2	47	-0.04	0.014	-1.02	0.804	-1.87	0.006	1.83	0.047	-0.61	0.016
Solyc03g058910	Pectate lyase	10.6.3	288	-0.04	-0.08	3.36	0.003	-1.08	0.913	3.64	0.019	0.08	0.777
Solyc12g042180	Arf-GAP	30.5	310	0.043	0.024	-1.55	0.142	1.47	0.096	-2.29	0.016	0.34	0.215
Solyc10g049390	F-box domain-containing protein	35.2	70	-0.04	-0.02	1.99	0.070	-1.49	0.191	2.97	0.029	-0.22	0.431
Solyc05g008560	Pentatricopeptide repeat-containing protein	17.8.1	429	0.043	0.018	-1.29	0.221	1.36	0.080	-1.75	0.040	0.39	0.151
Solyc12g011370	Amino acid transporter	34.3	441	-0.04	0.012	1.07	0.527	-1.65	0.009	1.76	0.077	-0.53	0.042
Solyc02g062460	2-oxoglutarate-dependent dioxygenase	17.5.1	84	-0.04	0.013	1.03	0.760	-1.62	0.005	1.67	0.050	-0.51	0.052
Solyc03g007060	Unknown Protein	35.2	663	0.042	-0.03	1.14	0.367	1.65	0.002	-1.45	0.468	0.46	0.084
Solyc01g105890	(E)-beta-ocimene synthase	16.1.5	746	-0.04	0.016	-1.11	0.775	-1.61	0.010	1.45	0.151	-0.46	0.084
Solyc07g042170	Jasmonate ZIM-domain protein 3	17.7.2	735	-0.04	0.018	1.07	0.805	-1.58	0.016	1.69	0.058	-0.37	0.175
Solyc06g007780	Ras GTPase-activating protein-binding protein 2	29.3.1	655	0.042	0.002	-1.08	0.741	1.52	0.011	-1.63	0.044	0.35	0.201
Solyc12g042860	Unknown Protein	35.2	602	-0.04	-0.01	1.38	0.276	-1.38	0.084	1.90	0.028	-0.21	0.453
Solyc01g098110	Hydrolase alpha/beta fold family protein	11.7	375	0.042	0.016	-1.50	0.004	1.37	0.016	-2.06	0.012	0.27	0.33
Solyc01g097770	Serine/threonine protein kinase	29.4	594	0.042	0.012	-1.27	0.364	1.53	0.068	-1.94	0.022	0.57	0.027
Solyc03g111110	Unknown Protein	35.2	126	0.042	0.004	-1.16	0.471	1.52	0.015	-1.76	0.015	0.46	0.084
Solyc01g108910	COSII_At2g15890	35.2	203	0.042	-0.01	-1.12	0.752	1.73	0.026	-1.94	0.042	0.13	0.644
Solyc04g082330	Pre-rRNA-processing protein TSR1	35.2	658	0.041	0	-1.10	0.633	1.57	0.002	-1.73	0.010	0.58	0.023
Solyc01g079570	Beta xylosidase	10.6.2	631	0.041	-0.06	1.49	0.293	2.02	0.009	-1.36	0.369	0.42	0.119
Solyc12g009420	Polygalacturonase	10.6.3	73	0.041	-0.01	1.19	0.575	1.66	0.017	-1.39	0.196	0.49	0.064
Solyc12g094500	Alcohol dehydrogenase 1	5.3	295	-0.04	0.011	1.06	0.942	-1.63	0.017	1.73	0.059	-0.63	0.012
Solyc09g089710	1-aminocyclopropane-1-carboxylate oxidase-like protein	17.5.1	93	0.041	0.023	-1.35	0.352	1.91	0.095	-2.57	0.007	0.34	0.215
Solyc12g098910	CBL-interacting protein kinase 1	29.4	347	-0.04	0.036	-1.33	0.360	-1.75	0.005	1.31	0.281	-0.43	0.11
Solyc03g093310	F-box family protein	29.5.11.4.3.2	546	0.041	-0.01	-1.18	0.882	1.58	0.006	-1.86	0.132	0.19	0.498
Solyc12g010300	Unknown Protein	35.2	261	0.041	0.049	-2.78	0.022	1.20	0.510	-3.34	0.012	-0.12	0.67
Solyc02g062490	2-oxoglutarate-dependent dioxygenase	17.6.1	576	-0.04	-0.01	1.37	0.221	-1.39	0.046	1.91	0.021	-0.18	0.521
Solyc12g043040	Sulfate transporter	34.6	106	0.041	0.019	-1.44	0.175	1.47	0.100	-2.12	0.048	0.22	0.431
Solyc11g022440	Unknown Protein	35.2	61	-0.04	0.042	-1.18	0.458	-2.05	0.003	1.74	0.104	-0.58	0.023
Solyc04g072160	Prostaglandin E synthase 3	35.2	511	-0.04	0.009	-1.07	0.743	-1.58	0.014	1.47	0.128	-0.52	0.047
Solyc12g008600	Unknown Protein	35.2	712	0.04	0.001	-1.26	0.272	1.45	0.018	-1.83	0.038	0.31	0.261
Solyc08g083230	Growth-regulating factor 3	27.3.50	578	0.04	0.013	-1.24	0.083	1.45	0.006	-1.80	0.015	0.45	0.092

Supplementary Table S1. continued

Solyc07g053250	Unknown Protein	35.2	412	0.04	0.009	-1.24	0.061	1.37	0.045	-1.70	0.071	0.43	0.11
Solyc04g071260	Actin	31.1	42	0.04	-0	-1.04	0.804	1.64	0.021	-1.70	0.040	0.51	0.052
Solyc10g054080	Kinesin like protein	31.1	713	0.04	0.006	-1.24	0.415	1.39	0.035	-1.73	0.050	0.21	0.453
Solyc02g078340	Heat stress transcription factor	20.2.1	140	-0.04	0.013	1.28	0.647	-1.60	0.034	2.04	0.023	-0.17	0.545
Solyc03g080010	MutS2 protein	28.2	188	0.04	-0	1.15	0.737	1.64	0.016	-1.43	0.091	0.45	0.092
Solyc06g051560	Flavoprotein wrbA	35.2	426	0.04	-0.1	3.31	0.070	2.76	0.014	1.20	0.801	0.35	0.201
Solyc11g007200	Copper chaperone	15.2	646	-0.04	-0.01	1.29	0.562	-1.36	0.048	1.75	0.003	-0.42	0.119
Solyc04g080380	Unknown Protein	35.2	9	0.04	0.008	-1.35	0.114	1.44	0.021	-1.94	0.010	0.39	0.151
Solyc03g059260	Carboxyl-terminal-processing protease	29.5	660	-0.04	0.055	-1.59	0.277	-1.85	0.022	1.16	0.556	-0.36	0.187
Solyc07g006010	Nucleoporin Nup43	33.99	81	0.04	-0.02	1.06	0.544	1.59	0.010	-1.49	0.183	0.62	0.014
Solyc06g072310	PROTODERMAL FACTOR 2	27.3.22	374	0.039	-0.02	1.17	0.468	1.65	0.004	-1.41	0.309	0.57	0.027
Solyc11g013530	PHD finger family protein	27.3.67	529	0.039	0.017	-1.26	0.330	1.39	0.040	-1.75	0.020	0.28	0.312
Solyc03g123370	(P)ppGpp synthetase I (GTP pyrophosphokinase) SpoT/RelA	30.3	391	-0.04	-0.03	1.59	0.112	-1.32	0.043	2.10	0.002	-0.39	0.151
Solyc10g076300	UPF0497 membrane protein At2g38480	35.2	109	0.039	-0	-1.16	0.313	1.56	0.000	-1.81	0.008	0.49	0.064
Solyc07g065730	Pentatricopeptide repeat-containing protein	27.3.25	117	0.039	0.009	-1.30	0.210	1.36	0.034	-1.77	0.058	0.29	0.294
Solyc02g089600	Ribosomal RNA-processing protein 7 homolog A	35.2	438	0.039	0.014	-1.28	0.219	1.34	0.044	-1.72	0.047	0.22	0.431
Solyc08g079120	NAC domain protein IPR003441	33.99	36	-0.04	-0.01	1.28	0.192	-1.47	0.037	1.89	0.045	-0.41	0.129
Solyc11g010560	Kinesin-like protein	31.1	724	0.039	-0.01	-1.03	0.744	1.47	0.010	-1.51	0.098	0.59	0.021
Solyc04g008430	Receptor like kinase, RLK	30.2.11	108	0.039	0.015	-1.30	0.260	1.34	0.068	-1.73	0.032	0.41	0.129
Solyc02g062180	Oxidoreductase 2OG-Fe oxygenase family protein	26.7	43	0.039	-0.01	-1.09	0.614	1.77	0.008	-1.93	0.002	0.29	0.294
Solyc07g065630	Ubiquitin-protein ligase 1	29.5.11.4.1	665	0.039	0.008	-1.18	0.278	1.42	0.017	-1.68	0.025	0.38	0.162
Solyc11g065890	1-acyl-sn-glycerol-3-phosphate acyltransferase	11.3	212	0.039	0.017	-1.43	0.001	1.31	0.066	-1.86	0.046	0.27	0.33
Solyc08g079840	Subtilisin-like protease	29.5.1	313	0.039	-0.01	-1.00	0.982	1.49	0.017	-1.49	0.145	0.57	0.027
Solyc02g093300	DNA polymerase	28.1	547	0.039	0.063	-2.09	0.052	1.07	0.814	-2.24	0.015	0.00	1
Solyc01g108630	Nitrite reductase	12.1.2	207	-0.04	-0.03	1.61	0.190	-1.45	0.069	2.33	0.012	-0.29	0.294
Solyc08g007080	Inositol 1 4 5-trisphosphate 5-phosphatase	28.1	193	0.039	0.021	-1.88	0.090	1.39	0.131	-2.61	0.016	-0.06	0.832
Solyc01g058170	Agenet domain-containing protein	35.2	440	0.038	-0.01	1.01	0.927	1.59	0.006	-1.57	0.097	0.61	0.016
Solyc03g082990	Tetratricopeptide repeat containing protein	31.1	199	0.038	0.002	-1.19	0.563	1.50	0.001	-1.79	0.033	0.48	0.07
Solyc02g011680	Pentatricopeptide repeat-containing protein	27.3.25	13	0.038	-0.02	1.14	0.646	1.62	0.012	-1.42	0.123	0.42	0.119
Solyc11g006500	Charged multivesicular body protein 5	27.3.71	350	-0.04	0.004	1.05	0.858	-1.60	0.020	1.68	0.105	-0.52	0.047
Solyc07g053540	Fasciclin-like arabinogalactan protein 4	10.5.1.1	141	0.038	-0.07	1.88	0.118	2.22	0.001	-1.18	0.617	0.55	0.034
Solyc04g082990	ORF42f	35.2	128	0.038	-0.03	1.11	0.696	1.84	0.018	-1.65	0.184	0.35	0.201
Solyc08g042040	Unknown Protein	35.2	224	0.038	-0.01	-1.00	0.975	1.57	0.019	-1.57	0.055	0.44	0.101
Solyc06g008810	Auxin F-box protein 5	17.2.2	518	0.038	-0.01	-1.11	0.458	1.70	0.000	-1.89	0.000	0.49	0.064
Solyc06g073060	Iaa-amino acid hydrolase 6	17.2.1	616	-0.04	0.003	1.09	0.646	-1.45	0.013	1.57	0.050	-0.34	0.215
Solyc11g031970	Unknown Protein	35.2	339	0.038	-0.02	1.10	0.452	1.52	0.015	-1.38	0.214	0.65	0.009
Solyc07g066520	Os03g0659800 protein	35.2	419	0.038	-0.01	1.09	0.812	1.61	0.009	-1.48	0.066	0.46	0.084
Solyc11g008770	LETM1 and EF-hand domain-containing protein 1	30.3	314	0.038	0.005	-1.07	0.518	1.41	0.031	-1.51	0.129	0.61	0.016
Solyc02g061960	La related protein-like	35.2	495	0.037	-0.01	-1.05	0.694	1.47	0.002	-1.54	0.044	0.63	0.012
Solyc06g072960	Lipid A export ATP-binding/permease protein msbA	34.16	52	0.037	-0.02	1.26	0.488	1.67	0.015	-1.32	0.192	0.79	0

Supplementary Table S1. continued

Solyc08g076820	BHLH transcription factor	27.3.6	560	0.037	0.014	-1.25	0.273	1.34	0.073	-1.67	0.029	0.25	0.369
Solyc03g119600	NIMIN2c protein	35.2	705	-0.04	-0	1.20	0.425	-1.40	0.026	1.69	0.051	-0.37	0.175
Solyc01g099750	Heparan-alpha-glucosaminide N-acetyltransferase	35.2	123	0.037	-0.03	1.04	0.944	1.92	0.019	-1.85	0.033	0.42	0.119
Solyc05g054870	Unknown Protein	35.2	293	0.037	0.011	-1.26	0.245	1.48	0.046	-1.86	0.046	0.42	0.119
Solyc02g093410	Protein kinase	29.4	531	-0.04	0.001	1.06	0.657	-1.41	0.003	1.50	0.037	-0.48	0.07
Solyc02g068320	2-oxoglutarate-dependent dioxygenase	17.5.1	710	-0.04	0.009	-1.03	0.865	-1.49	0.006	1.44	0.046	-0.58	0.023
Solyc06g036060	Zinc finger family protein	27.3.11	453	0.037	0.024	-1.44	0.041	1.25	0.139	-1.80	0.050	0.17	0.545
Solyc06g074630	Cellulose synthase-like C6 glycosyltransferase family 2	10.2	708	-0.04	-0.01	1.50	0.120	-1.40	0.034	2.10	0.010	-0.27	0.33
Solyc05g054530	Genomic DNA chromosome 5 BAC clone F2O15	35.2	727	0.037	-0	-1.07	0.435	1.45	0.002	-1.56	0.032	0.52	0.047
Solyc04g082610	Glutamate-gated kainate-type ion channel receptor	30.1.1	550	0.037	0.007	-1.13	0.531	1.42	0.021	-1.60	0.026	0.62	0.014
Solyc03g082900	Glucan endo-1 3-beta-glucosidase	26.4.1	414	0.037	-0.02	1.16	0.339	1.50	0.014	-1.30	0.274	0.74	0.002
Solyc07g061920	Glucan synthase like 3	3.6	468	0.037	0.02	-1.52	0.023	1.23	0.157	-1.87	0.034	0.14	0.619
Solyc06g009440	Pentatricopeptide repeat-containing protein	35.2	273	0.036	0.018	-1.74	0.006	1.29	0.116	-2.24	0.031	0.19	0.498
Solyc05g053070	Unknown Protein	35.2	753	-0.04	-0.01	1.33	0.179	-1.36	0.054	1.81	0.024	-0.39	0.151
Solyc02g073580	BZIP transcription factor	27.3.67	613	0.036	-0.16	8.82	0.031	4.34	0.013	2.03	0.339	0.32	0.245
Solyc12g038770	Unknown Protein	35.2	591	-0.04	0.061	-2.16	0.011	-2.03	0.003	-1.07	0.831	-0.64	0.01
Solyc03g026120	Dehydration-responsive family protein	20.2.3	260	-0.04	0.014	-1.16	0.509	-1.66	0.015	1.43	0.124	-0.50	0.058
Solyc04g005330	Periodic tryptophan protein 1 homolog	30.11	449	0.036	0.013	-1.28	0.203	1.34	0.066	-1.72	0.034	0.27	0.33
Solyc10g086350	Ras-related protein Rab-8A	30.5	625	-0.04	-0.01	1.37	0.105	-1.36	0.017	1.86	0.005	-0.44	0.101
Solyc08g023490	Pentatricopeptide repeat-containing protein	33.99	211	0.036	-0.01	1.09	0.386	1.51	0.004	-1.38	0.128	0.75	0.001
Solyc12g039080	Receptor-like kinase	30.2.99	67	0.036	-0.06	2.49	0.146	2.60	0.006	-1.04	0.728	0.25	0.369
Solyc01g044480	Elongation factor P family protein expressed	29.2.4	401	0.036	0.015	-1.35	0.103	1.30	0.092	-1.75	0.036	0.19	0.498
Solyc02g081940	RNA binding protein	27.4	559	0.036	0.005	-1.19	0.420	1.32	0.027	-1.58	0.070	0.25	0.369
Solyc11g008810	Beta-hexosaminidase b	29.7.5	408	0.035	0	-1.11	0.682	1.49	0.019	-1.65	0.028	0.20	0.475
Solyc07g054620	F-box/LRR-repeat protein 13	35.2	71	0.035	0.045	-2.27	0.022	1.24	0.304	-2.80	0.005	-0.13	0.644
Solyc06g071280	Enhanced disease susceptibility 1	20.1.3	641	-0.04	-0.01	1.19	0.422	-1.57	0.036	1.87	0.076	-0.36	0.187
Solyc02g085130	Tubby-like F-box protein 5	29.5.11.4.3.2	640	0.035	0.009	-1.28	0.027	1.34	0.015	-1.72	0.019	0.35	0.201
Solyc01g067220	Unknown Protein	35.2	197	0.035	0.026	-1.44	0.141	1.27	0.163	-1.83	0.021	0.15	0.594
Solyc04g039980	Lrr,resistance protein fragment	20.1	373	-0.04	-0.06	2.37	0.011	-1.11	0.540	2.64	0.004	-0.04	0.887
Solyc07g052590	Pentatricopeptide repeat-containing protein	27.3.25	248	0.035	-0.01	1.01	0.956	1.41	0.024	-1.40	0.170	0.43	0.11
Solyc02g062990	Glucosyltransferase	16.8.3	305	-0.04	0.11	-3.10	0.080	-2.63	0.007	-1.18	0.925	-0.48	0.07
Solyc08g062610	Cyclopropane-fatty-acyl-phospholipid synthase	11.3.10	262	0.035	-0.02	1.01	0.953	1.52	0.006	-1.51	0.085	0.50	0.058
Solyc03g096870	WD repeat-containing protein 5 homolog	33.99	741	0.035	-0.01	1.03	0.771	1.47	0.006	-1.43	0.190	0.40	0.14
Solyc06g083520	Nucleolar GTP-binding protein 2	30.5	564	0.035	0.005	-1.15	0.585	1.50	0.025	-1.72	0.014	0.22	0.431
Solyc07g006740	Multidrug resistance protein mdtK	34.99	16	-0.04	-0.02	1.37	0.025	-1.32	0.082	1.81	0.045	-0.38	0.162
Solyc03g059160	CASTOR protein	35.2	166	0.035	0.042	-1.83	0.012	1.07	0.595	-1.95	0.027	0.00	1
Solyc03g044370	OTU domain containing protein	29.5.3	406	0.035	-0.01	1.07	0.648	1.43	0.012	-1.34	0.157	0.62	0.014
Solyc06g007270	Fertility restorer	35.2	40	0.035	0.056	-2.22	0.014	1.04	0.826	-2.31	0.006	-0.06	0.832
Solyc01g100280	ATP-DEPENDENT DNA HELICASE	28.99	416	0.035	0.014	-1.31	0.072	1.34	0.017	-1.75	0.006	0.34	0.215

Supplementary Table S1. continued

Solyc05g051460	Homeobox-leucine zipper protein	27.3.22	351	-0.04	0.002	1.14	0.514	-1.40	0.006	1.60	0.008	-0.44	0.101
Solyc01g099220	DNA polymerase	28.1	422	0.034	-0.01	1.04	0.922	1.44	0.024	-1.39	0.136	0.67	0.006
Solyc12g044220	AT1G15240	35.2	442	0.034	-0.01	1.05	0.491	1.47	0.008	-1.40	0.151	0.64	0.01
Solyc06g084220	Unknown Protein	20.2.1	311	-0.03	0.002	1.03	0.668	-1.44	0.004	1.48	0.086	-0.46	0.084
Solyc03g062930	5&apos-3&apos exonuclease	35.2	463	0.034	0.018	-1.62	0.094	1.25	0.200	-2.03	0.009	0.23	0.41
Solyc05g050340	WRKY transcription factor 6	27.3.32	335	-0.03	-0.01	1.09	0.702	-1.54	0.025	1.68	0.086	-0.43	0.11
Solyc09g011380	BEL1-like homeodomain protein 9	27.3.22	158	0.034	-0.01	-1.02	0.852	1.49	0.005	-1.53	0.058	0.45	0.092
Solyc08g062340	Class II small heat shock protein Le-HSP17.6	20.2.1	329	-0.03	0.032	-1.36	0.153	-1.72	0.018	1.27	0.543	-0.65	0.009
Solyc10g086750	Unknown Protein	35.2	258	0.034	0.023	-1.44	0.086	1.26	0.142	-1.82	0.033	0.32	0.245
Solyc02g063390	DUF1295 domain protein	35.2	683	-0.03	-0.01	1.32	0.309	-1.33	0.032	1.75	0.008	-0.12	0.67
Solyc10g049890	Phosphoglycerate dehydrogenase	13.1.5.1.1	471	0.034	0	-1.05	0.685	1.40	0.016	-1.47	0.114	0.58	0.023
Solyc04g007070	Cc-nbs-lrr, resistance protein	20.1	685	-0.03	-0.04	2.03	0.092	-1.21	0.350	2.45	0.017	-0.24	0.389
Solyc07g041520	RNA polymerase-associated protein Ctr9 homolog	35.2	563	0.034	0.025	-1.48	0.072	1.22	0.213	-1.81	0.033	0.26	0.349
Solyc07g063360	Tir, resistance protein fragment	20.1.7	87	-0.03	-0.04	1.71	0.040	-1.22	0.382	2.09	0.052	-0.14	0.619
Solyc01g080020	Xylanase inhibitor	29.5.4	701	0.034	-0.05	1.37	0.138	1.64	0.003	-1.20	0.719	0.63	0.012
Solyc04g071140	Decarboxylase family protein	13.2.7	396	0.034	0.005	-1.24	0.276	1.41	0.021	-1.74	0.003	0.20	0.475
Solyc01g079650	CTP synthase-like protein	23.1.1.10	113	0.034	-0.01	1.16	0.179	1.51	0.012	-1.30	0.268	0.70	0.004
Solyc12g008420	RNA-dependent RNA polymerase family protein	27.2	561	0.034	-0.02	-1.05	0.939	1.41	0.008	-1.48	0.203	0.40	0.14
Solyc05g014700	Acid phosphatase/vanadium-dependent haloperoxidase related	35.2	204	-0.03	0.022	-1.15	0.517	-1.58	0.002	1.38	0.051	-0.55	0.034
Solyc06g064940	Phosphatidylinositol transfer protein SFH5	34.99	661	0.034	-0.02	-1.06	0.841	1.52	0.000	-1.61	0.176	0.26	0.349
Solyc03g034010	U-box domain-containing protein 5	29.5.11.4.2	365	0.034	0.027	-1.55	0.184	1.28	0.192	-1.97	0.001	0.20	0.475
Solyc05g052170	Acetyltransferase GNAT family protein	26.24	214	0.033	0.014	-1.34	0.145	1.38	0.017	-1.85	0.006	0.26	0.349
Solyc03g044550	Unknown Protein	35.2	232	-0.03	-0.05	1.93	0.068	-1.19	0.300	2.29	0.018	-0.11	0.696
Solyc06g054240	5&apos-nucleotidase surE	26.13	182	0.033	-0.01	-1.01	0.871	1.46	0.029	-1.49	0.124	0.41	0.129
Solyc01g106100	DUF593-containing protein 2	35.2	407	0.033	-0	-1.14	0.280	1.35	0.015	-1.54	0.054	0.34	0.215
Solyc08g077430	Glycine-rich protein	35.2	630	0.033	0.023	-1.49	0.044	1.28	0.063	-1.91	0.009	0.20	0.475
Solyc12g044440	Unknown Protein	30.5	513	0.033	0.032	-1.59	0.032	1.19	0.192	-1.89	0.006	0.05	0.86
Solyc01g099230	Zinc finger protein VAR3, chloroplastic	30.5	554	0.033	0.007	-1.18	0.535	1.35	0.024	-1.59	0.045	0.41	0.129
Solyc01g105080	Receptor like kinase, RLK	30.2.3	144	-0.03	-0.06	3.02	0.021	-1.03	0.825	3.10	0.004	0.07	0.804
Solyc03g113760	E2F transcription factor-like protein	27.3.18	384	0.033	0.011	-1.25	0.092	1.29	0.054	-1.61	0.041	0.21	0.453
Solyc05g009710	Mediator of RNA polymerase II transcription subunit 25	30.11	565	0.033	0.02	-1.41	0.089	1.23	0.142	-1.74	0.022	0.17	0.545
Solyc12g014500	S-adenosyl-L-methionine salicylic acid carboxyl methyltransferase	17.8.1	142	-0.03	0.011	-1.04	0.828	-1.47	0.010	1.41	0.076	-0.60	0.018
Solyc05g048810	tRNA-specific adenosine deaminase	27.1	31	-0.03	0.023	-1.32	0.358	-1.70	0.002	1.29	0.125	-0.53	0.042
Solyc04g049360	4-amino-4-deoxychorismate synthase component I	13.1.6.5.1	330	0.033	0.033	-1.88	0.022	1.12	0.465	-2.11	0.016	0.02	0.944
Solyc10g018030	Homeobox-leucine zipper protein ROC7	27.3.22	26	-0.03	-0.02	1.58	0.023	-1.42	0.053	2.24	0.015	-0.31	0.261
Solyc01g010370	Dehydration-responsive protein-like	20.2.3	649	0.033	-0	-1.15	0.444	1.33	0.011	-1.53	0.073	0.37	0.175
Solyc06g082130	Pyruvate decarboxylase 2	5.2	118	0.033	0.006	-1.35	0.213	1.36	0.033	-1.84	0.023	0.20	0.475

Supplementary Table S1. continued

Solyc11g017130	Forkhead-associated domain-containing protein	27.3.48	413	0.033	0.008	-1.37	0.246	1.26	0.062	-1.73	0.049	0.04	0.887
Solyc07g056480	Glutathione S-transferase-like protein	26.9	562	-0.03	0.001	1.26	0.367	-1.36	0.033	1.72	0.014	-0.22	0.431
Solyc02g091710	von Willebrand factor type A domain-containing protein	29.5.11.4.2	595	0.032	-0.02	1.11	0.430	1.45	0.005	-1.31	0.191	0.74	0.002
Solyc03g116900	Manganese transporter MntH	34.12	473	0.032	0.012	-1.44	0.011	1.32	0.024	-1.90	0.008	0.25	0.369
Solyc11g068370	BZIP transcription factor	27.3.35	116	-0.03	0.053	-1.61	0.273	-2.16	0.007	1.34	0.379	-0.68	0.005
Solyc03g078770	UDP-glucuronosyltransferase	26.2	320	0.032	-0.06	1.62	0.076	1.74	0.020	-1.07	0.676	0.56	0.03
Solyc11g066150	Bifunctional polymyxin resistance protein ArnA	10.1.5	195	-0.03	-0.06	2.49	0.003	-1.14	0.709	2.84	0.022	-0.10	0.723
Solyc10g076410	Abscisic acid receptor PYL4	20.2.99	161	0.032	0.018	-1.62	0.112	1.22	0.192	-1.98	0.028	-0.04	0.887
Solyc06g065800	Mitochondrial glycoprotein family protein	35.2	155	0.032	-0.01	1.01	0.930	1.49	0.010	-1.47	0.012	0.49	0.064
Solyc02g061910	Unknown Protein	27.3.27	115	0.032	0.017	-1.47	0.133	1.31	0.167	-1.93	0.018	0.07	0.804
Solyc07g043610	Auxin response factor 6	27.3.4	526	0.032	0.005	-1.15	0.386	1.37	0.024	-1.57	0.094	0.49	0.064
Solyc12g056630	Holocarboxylase synthetase	35.2	643	0.032	0.007	-1.12	0.443	1.38	0.011	-1.55	0.014	0.52	0.047
Solyc05g054860	Pto-like, Serine/threonine kinase protein, resistance protein	29.4.1.57	430	0.032	-0.04	1.26	0.200	1.69	0.001	-1.34	0.273	0.55	0.034
Solyc12g007070	Heat stress transcription factor A3	20.2.1	428	0.032	0.035	-2.03	0.000	1.15	0.175	-2.33	0.003	-0.02	0.944
Solyc05g005990	Solute carrier family 15 member 2	34.13	525	-0.03	0	1.16	0.436	-1.33	0.033	1.55	0.055	-0.26	0.349
Solyc09g057870	Protein FAR1-RELATED SEQUENCE 5	30.11	309	0.032	0.002	-1.12	0.380	1.36	0.024	-1.52	0.050	0.46	0.084
Solyc02g062760	GATA transcription factor 19	27.3.9	163	0.032	0.006	-1.42	0.059	1.40	0.019	-1.99	0.009	0.27	0.33
Solyc05g053990	Cc-nbs-lrr, resistance protein	20.1	654	0.032	-0.03	1.18	0.680	1.68	0.016	-1.42	0.110	0.37	0.175
Solyc04g077850	U-box domain-containing protein 4	29.5.11.4.2	439	-0.03	0.023	-1.26	0.118	-1.53	0.008	1.21	0.452	-0.52	0.047
Solyc08g083190	Hydrolase alpha/beta fold family protein expressed	28.1	210	0.031	0.051	-2.03	0.069	1.18	0.634	-2.40	0.000	0.16	0.569
Solyc01g107250	LRR receptor-like serine/threonine-protein kinase, RLP	30.2.11	372	-0.03	-0.01	1.13	0.498	-1.32	0.035	1.49	0.058	-0.45	0.092
Solyc11g069600	Inter-alpha-trypsin inhibitor heavy chain H4	29.5.11.4.2	699	0.031	-0.01	-1.01	0.935	1.43	0.003	-1.44	0.040	0.74	0.002
Solyc02g079010	Cotton fiber expressed protein 1	35.2	234	-0.03	-0.01	1.28	0.231	-1.31	0.055	1.68	0.038	-0.39	0.151
Solyc11g066330	Nodulin family protein	33.99	447	0.031	-0.03	1.03	0.833	1.52	0.013	-1.48	0.169	0.37	0.175
Solyc09g098310	Unknown Protein	35.2	497	-0.03	-0.03	1.65	0.061	-1.20	0.308	1.97	0.025	-0.18	0.521
Solyc01g066990	Unknown Protein	35.2	579	0.031	0.012	-1.20	0.236	1.29	0.053	-1.54	0.038	0.39	0.151
Solyc09g057920	N-alpha-acetyltransferase 25, NatB auxiliary subunit	35.2	242	0.031	0.007	-1.16	0.254	1.28	0.034	-1.49	0.076	0.33	0.23
Solyc03g025600	Pectinacetylesterase like protein	10.8.2	512	0.031	-0.02	1.14	0.465	1.51	0.004	-1.33	0.140	0.66	0.007
Solyc09g018610	Unknown Protein	35.2	165	-0.03	-0.04	2.07	0.007	-1.13	0.766	2.35	0.053	-0.15	0.594
Solyc06g082920	Receptor like kinase, RLK	30.2.11	569	-0.03	-0.01	1.31	0.309	-1.32	0.078	1.73	0.025	-0.47	0.077
Solyc01g014120	Unknown Protein	35.2	63	0.031	0	-1.20	0.314	1.32	0.028	-1.58	0.027	0.41	0.129
Solyc07g032170	Abhydrolase domain-containing protein 5	35.2	634	0.031	0.035	-1.61	0.076	1.17	0.401	-1.88	0.005	0.04	0.887
Solyc02g089530	Unknown Protein	35.2	150	0.031	0.021	-1.56	0.070	1.17	0.215	-1.83	0.023	0.17	0.545
Solyc03g113300	Short-chain dehydrogenase/reductase SDR	26.22	678	-0.03	0.013	-1.01	0.997	-1.48	0.010	1.47	0.120	-0.60	0.018
Solyc03g119330	26S proteasome regulatory subunit	35.2	323	0.031	-0.01	1.01	0.928	1.40	0.006	-1.39	0.092	0.62	0.014
Solyc01g100000	F-box protein PP2-B1	31.1	249	0.031	0.015	-1.36	0.083	1.32	0.025	-1.79	0.004	0.20	0.475
Solyc12g056820	Laccase	16.10	8	-0.03	-0.03	1.70	0.072	-1.23	0.195	2.08	0.018	-0.05	0.86

Supplementary Table S1. continued

Solyc09g065690	Transcription factor jumonji	35.2	567	0.03	0.01	-1.25	0.122	1.34	0.010	-1.67	0.002	0.46	0.084
Solyc04g011630	GRAS family transcription factor	27.3.21	527	0.03	0.007	-1.15	0.422	1.32	0.022	-1.51	0.026	0.31	0.261
Solyc11g017470	NAC domain protein IPR003441	33.99	618	-0.03	0.02	-1.19	0.228	-1.58	0.003	1.32	0.215	-0.56	0.03
Solyc08g079760	Unknown Protein	35.2	601	0.03	0.008	-1.34	0.230	1.26	0.043	-1.69	0.020	0.04	0.887
Solyc02g087040	RING finger protein	29.5.11.4.2	192	0.03	-0	-1.05	0.668	1.31	0.027	-1.38	0.127	0.50	0.058
Solyc11g005720	VQ motif family protein	35.2	399	-0.03	0.003	1.07	0.672	-1.39	0.008	1.48	0.043	-0.43	0.11
Solyc03g025390	Retinol dehydrogenase 12	26.22	568	-0.03	0.011	-1.03	0.942	-1.37	0.003	1.33	0.119	-0.48	0.07
Solyc08g076320	Cellulose synthase-like protein	10.2.1	745	0.03	-0.01	-1.08	0.697	1.31	0.013	-1.41	0.121	0.44	0.101
Solyc04g016180	Glycosyltransferase	35.2	173	0.03	0.011	-1.16	0.224	1.31	0.048	-1.52	0.084	0.34	0.215
Solyc02g090340	Cytochrome P450	26.10	744	-0.03	0.02	1.03	0.996	-1.47	0.019	1.52	0.056	-0.29	0.294
Solyc06g074530	Prephenate dehydratase	13.1.6.3.1	728	-0.03	0.005	1.06	0.824	-1.38	0.012	1.46	0.031	-0.37	0.175
Solyc09g075530	Soluble NSF Attachment Protein	31.4	96	0.03	0.041	-2.03	0.014	1.10	0.568	-2.23	0.007	0.04	0.887
Solyc09g075230	POT family domain containing protein expressed	35.2	604	-0.03	-0	1.17	0.178	-1.33	0.009	1.56	0.014	-0.40	0.14
Solyc08g075010	Glucose-repressible alcohol dehydrogenase transcriptional effector	28.1	502	0.029	0.039	-1.76	0.029	1.09	0.526	-1.92	0.017	-0.11	0.696
Solyc10g079350	UDP-glucosyltransferase HvUGT5876	26.2	254	-0.03	0.02	1.04	0.902	-1.97	0.016	2.04	0.125	-0.45	0.092
Solyc10g084040	Thylakoid lumen 15.0-kDa protein	35.2	332	-0.03	-0.02	1.36	0.200	-1.29	0.101	1.76	0.043	-0.42	0.119
Solyc04g058170	Calmodulin-like protein	30.3	64	-0.03	0.027	-1.31	0.258	-1.55	0.000	1.18	0.271	-0.69	0.004
Solyc03g007330	Cell division protease ftsH homolog	29.5.7	722	0.029	0.006	-1.15	0.417	1.28	0.058	-1.47	0.043	0.33	0.23
Solyc01g067710	Sodium/hydrogen exchanger	34.14	677	0.029	0.039	-1.60	0.079	1.17	0.341	-1.88	0.004	0.07	0.804
Solyc10g006870	Pto-like, Serine/threonine kinase protein	29.4.1.57	574	0.029	0.002	-1.18	0.373	1.28	0.021	-1.50	0.039	0.23	0.41
Solyc04g054260	Cytochrome P450	26.10	95	0.029	0.021	-1.53	0.020	1.28	0.061	-1.97	0.023	0.17	0.545
Solyc03g033850	Syntaxin 81	31.4	501	-0.03	0.021	-1.29	0.269	-1.53	0.014	1.19	0.410	-0.51	0.052
Solyc05g012210	Ninja-family protein 1	29.3.1	694	-0.03	0.026	-1.31	0.295	-1.62	0.001	1.24	0.324	-0.56	0.03
Solyc01g101250	D111/G-patch domain-containing protein	35.2	127	0.029	0.062	-2.29	0.021	-1.04	0.918	-2.21	0.026	-0.07	0.804
Solyc03g059110	Unknown Protein	35.2	686	0.029	0.012	-1.38	0.032	1.19	0.178	-1.64	0.052	0.28	0.312
Solyc02g093020	Os06g0524700 protein	17.5.2	337	-0.03	-0.02	1.32	0.125	-1.28	0.098	1.69	0.039	-0.35	0.201
Solyc09g059990	Zinc finger family protein	29.5.11.4.2	503	0.029	-0.02	1.11	0.405	1.43	0.001	-1.29	0.183	0.64	0.01
Solyc04g014610	Armadillo repeat-containing protein 7	35.2	46	0.029	0.004	-1.18	0.346	1.29	0.034	-1.52	0.044	0.27	0.33
Solyc12g099360	Acyl-CoA synthetase/AMP-acid ligase II	11.1.8	59	-0.03	-0.03	1.62	0.006	-1.25	0.156	2.02	0.021	-0.14	0.619
Solyc01g079520	DNA mismatch repair protein muts	28.2	605	0.029	0.019	-1.35	0.077	1.20	0.124	-1.62	0.014	0.19	0.498
Solyc04g057940	U-box domain-containing protein	29.5.11.4.2	241	0.029	-0.08	2.65	0.064	2.51	0.004	1.05	0.707	0.62	0.014
Solyc04g074410	Os06g0220000 protein	30.1.1	751	0.029	0.01	-1.37	0.149	1.25	0.102	-1.72	0.027	0.05	0.86
Solyc06g059900	Unknown Protein	35.2	98	-0.03	0	1.06	0.735	-1.45	0.013	1.53	0.065	-0.53	0.042
Solyc03g083000	AT2G46550 protein	35.2	222	-0.03	0.008	-1.12	0.327	-1.46	0.002	1.30	0.140	-0.58	0.023
Solyc05g015980	Unknown Protein	35.2	472	0.029	0.001	-1.22	0.317	1.31	0.013	-1.60	0.042	0.20	0.475
Solyc09g008850	Uridylate kinase	23.4.3	247	0.029	-0.02	1.23	0.483	1.56	0.014	-1.27	0.188	0.46	0.084
Solyc05g054170	Scarecrow-like 1 transcription factor	27.3.21	669	-0.03	-0	1.11	0.574	-1.36	0.011	1.51	0.015	-0.56	0.03
Solyc06g054630	Ethylene-responsive transcription factor 1	27.3.3	83	-0.03	-0.02	1.31	0.139	-1.30	0.038	1.70	0.022	-0.36	0.187
Solyc09g074210	Endonuclease/exonuclease/phosphatase family protein	28.1	289	0.028	-0.05	1.36	0.380	1.82	0.006	-1.33	0.241	0.45	0.092
Solyc11g065210	Ubiquitin carboxyl-terminal hydrolase	29.5.11.5	437	0.028	0.017	-1.26	0.076	1.21	0.080	-1.52	0.032	0.25	0.369

Supplementary Table S1. continued

Solyc10g084250	Receptor-like kinase	30.2.17	431	0.028	-0.03	1.11	0.550	1.46	0.010	-1.31	0.254	0.41	0.129
Solyc07g053520	Pentatricopeptide repeat-containing protein	29.4	137	0.028	0.005	-1.14	0.380	1.31	0.010	-1.49	0.022	0.34	0.215
Solyc12g014040	Chloroplast protein HCF243	35.2	535	0.028	-0	-1.02	0.790	1.37	0.010	-1.40	0.046	0.61	0.016
Solyc12g094660	Cc-nbs-lrr, resistance protein	20.1	334	-0.03	-0.04	1.94	0.027	-1.20	0.426	2.32	0.031	-0.27	0.33
Solyc02g089560	Pentatricopeptide repeat-containing protein	35.2	612	0.028	0.013	-1.25	0.215	1.23	0.093	-1.55	0.033	0.34	0.215
Solyc04g078670	NAC domain transcription factor	33.99	215	-0.03	0.047	-1.67	0.043	-1.78	0.001	1.07	0.748	-0.65	0.009
Solyc06g007120	1-phosphatidylinositol-4 5-bisphosphate phosphodiesterase	30.4.4	674	-0.03	0.018	-1.14	0.278	-1.51	0.007	1.32	0.221	-0.59	0.021
Solyc08g023460	Cell cycle control protein	34.1	266	0.028	0.018	-1.31	0.100	1.21	0.116	-1.58	0.049	0.28	0.312
Solyc08g066700	Polyribonucleotide nucleotidyltransferase	27.1.19	723	0.028	0.007	-1.15	0.545	1.30	0.022	-1.50	0.033	0.28	0.312
Solyc06g007620	POT family domain containing protein expressed	35.2	510	0.028	-0.01	-1.04	0.629	1.37	0.002	-1.43	0.039	0.53	0.042
Solyc09g065160	Dynamin family protein	30.5	555	0.028	0.002	-1.11	0.333	1.30	0.030	-1.45	0.078	0.56	0.03
Solyc09g065590	Os12g0604200 protein	35.2	720	0.028	0.008	-1.13	0.319	1.26	0.039	-1.43	0.037	0.41	0.129
Solyc02g092670	Subtilisin-like protease	29.5.1	491	0.028	-0.05	1.61	0.096	1.65	0.014	-1.03	0.788	0.56	0.03
Solyc10g055700	Pentatricopeptide repeat-containing protein	35.2	153	0.028	0	-1.04	0.702	1.39	0.002	-1.44	0.042	0.69	0.004
Solyc02g085010	Unknown Protein	35.2	169	-0.03	0.012	-1.03	0.833	-1.44	0.015	1.40	0.093	-0.35	0.201
Solyc09g057680	Receptor like kinase, RLK	29.4.1.57	97	-0.03	-0.04	2.31	0.032	-1.11	0.608	2.56	0.013	-0.03	0.915
Solyc02g032650	Nbs-lrr, resistance protein	20.1.7	275	-0.03	0.096	-3.07	0.029	-2.60	0.019	-1.18	0.571	-0.51	0.052
Solyc09g083250	AT5g02940/F9G14_250	35.2	256	0.028	0.009	-1.25	0.178	1.32	0.028	-1.66	0.006	0.18	0.521
Solyc02g078230	Glucan synthase like 1	3.6	614	0.028	-0	-1.11	0.541	1.27	0.016	-1.41	0.104	0.44	0.101
Solyc01g095700	Abscisic acid receptor PYL8	20.2.99	125	-0.03	-0.04	1.75	0.007	-1.09	0.497	1.92	0.008	-0.10	0.723
Solyc02g079180	Heat shock transcription factor 1	20.2.1	21	0.027	0.025	-1.49	0.059	1.20	0.143	-1.79	0.002	0.28	0.312
Solyc01g096940	Receptor like kinase, RLK	29.4.1.57	593	0.027	-0.01	1.07	0.490	1.45	0.001	-1.36	0.041	0.62	0.014
Solyc08g016430	Unknown Protein	35.2	583	0.027	0.016	-1.29	0.061	1.23	0.078	-1.58	0.047	0.38	0.162
Solyc10g086500	3-oxo-5-alpha-steroid 4-dehydrogenase family protein expressed	17.3.1.1.1	750	-0.03	0.006	1.14	0.501	-1.31	0.014	1.49	0.022	-0.19	0.498
Solyc08g080570	UDP-glucose 4-epimerase	10.1.2	719	-0.03	-0.03	1.63	0.030	-1.11	0.289	1.82	0.003	-0.09	0.75
Solyc00g031360	D-serine dehydratase	35.2	202	-0.03	-0.01	1.29	0.040	-1.31	0.010	1.68	0.006	-0.35	0.201
Solyc09g008060	UDP-glucosyltransferase family 1 protein	26.2	552	-0.03	0.006	1.02	0.987	-1.36	0.023	1.39	0.054	-0.45	0.092
Solyc03g093450	Receptor-like protein kinase	29.4.1.57	225	-0.03	0.029	-1.25	0.382	-1.59	0.007	1.28	0.212	-0.62	0.014
Solyc11g069470	Class III homeodomain-leucine zipper	27.3.22	749	0.027	-0	-1.06	0.579	1.27	0.030	-1.35	0.134	0.40	0.14
Solyc11g011880	RLK, Receptor like protein	30.2.17	72	0.027	-0.08	2.39	0.035	1.93	0.004	1.24	0.399	0.73	0.002
Solyc04g007470	CONSTANS-like zinc finger protein	27.3.7	14	0.027	0.054	-2.29	0.020	1.09	0.742	-2.49	0.002	-0.13	0.644
Solyc08g080270	3&apos-phosphoadenosine 5&apos-phosphosulfate transporter 1	34.11	30	-0.03	-0.04	1.59	0.123	-1.27	0.143	2.01	0.038	-0.12	0.67
Solyc01g091320	Sterol 4-alpha-methyl-oxidase 2	11.8.2	718	-0.03	0.011	-1.03	0.895	-1.33	0.003	1.29	0.136	-0.41	0.129
Solyc01g091780	Fructosamine kinase family protein	35.2	53	0.027	-0.02	1.17	0.123	1.54	0.001	-1.31	0.146	0.71	0.003
Solyc11g044960	Histidyl-tRNA synthetase	29.4	581	0.027	0.007	-1.22	0.123	1.23	0.040	-1.51	0.019	0.23	0.41
Solyc03g044380	Histone-lysine N-methyltransferase MEDEA	27.3.99	542	0.027	-0	-1.06	0.928	1.33	0.004	-1.40	0.117	0.38	0.162
Solyc01g095930	O-acyltransferase WSD1	35.2	69	0.026	0.046	-2.10	0.089	1.30	0.424	-2.72	0.008	0.17	0.545
Solyc03g082610	Arabidopsis thaliana genomic DNA chromosome 5 P1 clone MOK16	35.2	652	-0.03	-0.01	1.19	0.341	-1.25	0.036	1.48	0.030	-0.22	0.431

Supplementary Table S1. continued

Solyc09g010880	Rhomboid family protein	29.5.5	383	-0.03	-0.03	1.44	0.113	-1.17	0.175	1.69	0.008	-0.12	0.67
Solyc02g083350	DNA-directed RNA polymerase	27.2	466	0.026	0.005	-1.04	0.760	1.36	0.035	-1.41	0.076	0.35	0.201
Solyc10g074860	Unknown Protein	35.2	689	-0.03	-0.03	1.46	0.083	-1.22	0.108	1.78	0.007	-0.24	0.389
Solyc09g055230	ATP-dependent Clp protease ATP-binding subunit	20.2.1	445	0.026	0.009	-1.23	0.008	1.24	0.018	-1.52	0.017	0.32	0.245
Solyc09g075810	Blue copper protein	26.19	715	-0.03	-0.07	2.48	0.077	-1.03	0.705	2.55	0.009	-0.01	0.972
Solyc04g071660	Unknown Protein	35.2	657	0.026	0.008	-1.25	0.130	1.23	0.016	-1.54	0.006	0.23	0.41
Solyc04g016130	F-box/LRR-repeat protein At5g63520	29.5.11.4.3.2	515	0.026	0.003	-1.17	0.065	1.30	0.002	-1.52	0.004	0.54	0.038
Solyc07g005960	Serine carboxypeptidase K10B2.2	29.5.5	609	-0.03	0.017	-1.02	0.829	-1.48	0.019	1.45	0.146	-0.53	0.042
Solyc12g006970	Unknown Protein	35.2	138	0.026	0	1.00	0.928	1.35	0.028	-1.35	0.052	0.50	0.058
Solyc01g005790	Pentatricopeptide repeat-containing protein	27.3.25	322	0.026	0.009	-1.26	0.004	1.25	0.015	-1.57	0.014	0.35	0.201
Solyc11g019910	Pectinesterase	26.18	543	0.026	-0.01	1.14	0.396	1.44	0.007	-1.27	0.178	0.57	0.027
Solyc05g055730	Methylenetetrahydrofolate reductase	25	645	0.026	-0.03	1.64	0.079	1.61	0.015	1.02	0.973	0.71	0.003
Solyc06g073350	Calcium-dependent protein kinase 2	29.4	60	0.026	0.015	-1.45	0.057	1.21	0.193	-1.76	0.036	0.15	0.594
Solyc06g054670	Stearoyl-acyl carrier protein desaturase	11.1.15	15	-0.03	-0.01	1.19	0.391	-1.31	0.056	1.55	0.042	-0.42	0.119
Solyc11g064760	Disease resistance protein R3a-like protein	35.2	312	0.026	0.017	-1.21	0.213	1.22	0.083	-1.47	0.026	0.33	0.23
Solyc08g008360	Unknown Protein	35.2	606	0.026	0.014	-1.31	0.093	1.21	0.065	-1.59	0.042	0.12	0.67
Solyc08g006080	Exostosin	26.2	348	0.026	-0.01	1.07	0.604	1.41	0.005	-1.32	0.097	0.75	0.001
Solyc01g105150	Potassium transporter family protein	34.15	598	0.026	-0	-1.15	0.423	1.26	0.020	-1.45	0.075	0.25	0.369
Solyc01g059870	Phytochrome B1	30.11	548	-0.03	-0.02	1.43	0.106	-1.21	0.192	1.73	0.035	-0.22	0.431
Solyc02g092830	Unknown Protein	35.2	621	0.026	-0	-1.09	0.617	1.27	0.010	-1.38	0.060	0.41	0.129
Solyc08g007100	HAD-superfamily hydrolase subfamily IA variant 3	35.2	209	0.025	0.013	-1.39	0.023	1.19	0.130	-1.65	0.030	0.22	0.431
Solyc02g072000	Heat stress transcription factor A3	27.3.23	218	-0.03	0.005	1.00	0.982	-1.39	0.003	1.40	0.024	-0.54	0.038
Solyc06g072770	RNA recognition motif-containing protein	27.4	397	0.025	0.036	-1.84	0.155	1.26	0.112	-2.32	0.034	0.21	0.453
Solyc11g017240	Chorismate mutase 2	13.1.6.2.1	585	0.025	-0.02	1.13	0.368	1.41	0.004	-1.25	0.135	0.54	0.038
Solyc05g012770	WRKY transcription factor-a	27.3.32	389	-0.03	-0.02	1.62	0.001	-1.19	0.301	1.94	0.030	-0.13	0.644
Solyc02g085670	Major facilitator superfamily MFS_1	34.99	650	0.025	0	-1.06	0.541	1.30	0.021	-1.38	0.069	0.65	0.009
Solyc10g050440	Unknown Protein	35.2	530	-0.03	-0.02	1.35	0.167	-1.21	0.152	1.63	0.022	-0.14	0.619
Solyc06g050980	Ferritin	15.2	706	-0.03	-0.03	1.81	0.029	-1.03	0.819	1.87	0.016	0.15	0.594
Solyc03g112550	Kinetochore protein Spc25	35.2	596	0.025	0.01	-1.32	0.161	1.22	0.011	-1.61	0.011	0.07	0.804
Solyc07g066090	SEC14 cytosolic factor family protein	34.99	205	-0.03	0.053	-1.97	0.066	-1.75	0.024	-1.12	0.541	-0.52	0.047
Solyc01g099380	U4/U6.U5 tri-snRNP-associated protein 1	33.99	316	0.025	0.025	-1.44	0.016	1.15	0.198	-1.65	0.016	0.16	0.569
Solyc03g114010	Pentatricopeptide repeat protein	29.4	227	0.025	0.001	-1.06	0.781	1.27	0.015	-1.35	0.086	0.40	0.14
Solyc12g096340	UDP-sugar transporter-like protein	34	180	-0.03	0.024	-1.29	0.356	-1.59	0.020	1.24	0.372	-0.61	0.016
Solyc01g101100	Receptor-like protein kinase	29.4.1.57	403	0.025	-0.02	1.14	0.539	1.46	0.005	-1.28	0.144	0.51	0.052
Solyc02g068900	Glutathione transferase	26.9	423	-0.03	0.008	-1.05	0.733	-1.33	0.013	1.27	0.099	-0.56	0.03
Solyc02g090380	Nbs-lrr, resistance protein	20.1.7	358	-0.03	0.001	-1.00	0.996	-1.40	0.016	1.39	0.150	-0.46	0.084
Solyc02g087150	Heavy-metal-associated domain-containing protein	35.2	504	-0.03	-0.01	1.49	0.055	-1.20	0.242	1.78	0.028	-0.32	0.245
Solyc11g017440	Snakin-2	17.6.3	304	-0.03	-0.09	2.52	0.046	1.02	0.886	2.46	0.042	0.11	0.696
Solyc08g076060	Serine/threonine kinase receptor	30.2.17	277	0.025	-0.12	3.56	0.012	2.52	0.001	1.41	0.297	0.86	0
Solyc05g012650	T7.1 protein	35.2	421	0.025	-0.01	-1.02	0.960	1.29	0.005	-1.31	0.099	0.58	0.023
Solyc01g006890	Genomic DNA chromosome 5 P1 clone MUK11	35.2	671	-0.03	0.007	-1.05	0.541	-1.39	0.018	1.32	0.213	-0.52	0.047

Supplementary Table S1. continued

Solyc05g050520	NC domain-containing protein	35.2	191	0.025	0.047	-2.36	0.008	1.04	0.797	-2.46	0.005	-0.19	0.498
Solyc10g044480	Transmembrane 9 superfamily protein member 3	35.2	496	0.025	-0.01	1.04	0.672	1.35	0.008	-1.29	0.201	0.48	0.07
Solyc03g114290	UPF0497 membrane protein At2g36330	35.2	435	0.025	-0.01	1.04	0.840	1.35	0.004	-1.30	0.027	0.79	0
Solyc07g049740	Lipid-A-disaccharide synthase-like protein	35.2	278	0.025	0.016	-1.51	0.159	1.24	0.254	-1.86	0.010	0.14	0.619
Solyc03g098460	Retinol dehydrogenase 12	33.99	729	-0.03	0.007	1.11	0.522	-1.30	0.023	1.45	0.046	-0.23	0.41
Solyc01g109650	Receptor like kinase, RLK	29.4.1.57	516	0.024	0.013	-1.45	0.009	1.14	0.169	-1.66	0.021	0.12	0.67
Solyc02g069930	Lipase-like protein	11.9.2	194	0.024	-0.03	1.51	0.038	1.63	0.000	-1.08	0.486	0.90	0
Solyc04g045580	Os04g0386500 protein	35.2	420	0.024	0.009	-1.15	0.395	1.28	0.070	-1.48	0.024	0.38	0.162
Solyc10g047010	Ulp1 protease family C-terminal catalytic domain containing protein	35.2	5	-0.02	0.015	1.01	0.932	-1.44	0.004	1.46	0.005	-0.68	0.005
Solyc02g062500	2-oxoglutarate-dependent dioxygenase	17.6.1	448	-0.02	0.002	-1.01	0.917	-1.32	0.017	1.31	0.079	-0.46	0.084
Solyc06g082570	Unknown Protein	35.2	477	-0.02	0.024	-1.18	0.588	-1.45	0.001	1.23	0.243	-0.54	0.038
Solyc12g057050	Aquaporin	34.19.2	49	-0.02	0.008	-1.12	0.417	-1.45	0.018	1.29	0.279	-0.56	0.03
Solyc10g074470	Bzip transcription factor-like	27.3.35	588	0.024	-0.02	1.13	0.246	1.36	0.005	-1.21	0.262	0.66	0.007
Solyc09g031650	Zinc finger protein	35.2	623	0.024	0.015	-1.25	0.261	1.24	0.111	-1.55	0.002	0.42	0.119
Solyc08g075940	DNA-directed RNA polymerase	27.2	571	-0.02	0.014	1.01	0.940	-1.38	0.021	1.40	0.122	-0.37	0.175
Solyc05g009350	Zinc finger CCCH domain-containing protein 65	27.3.99	538	0.024	0.014	-1.26	0.097	1.17	0.125	-1.48	0.025	0.19	0.498
Solyc01g087850	Subtilisin-like protease	29.5.1	487	0.024	-0.05	1.51	0.100	1.56	0.013	-1.04	0.798	0.78	0.001
Solyc06g083140	Auxin-induced in root cultures protein 12	17.2.3	485	0.024	-0.06	1.96	0.088	1.89	0.007	1.04	0.954	0.48	0.07
Solyc09g005320	E3 ubiquitin-protein ligase RNF34	29.5.11.4.2	398	0.024	0.005	-1.17	0.148	1.25	0.038	-1.46	0.049	0.48	0.07
Solyc01g094940	Receptor-like protein kinase	29.4.1.57	607	0.024	0	-1.21	0.476	1.27	0.008	-1.54	0.065	0.15	0.594
Solyc01g095510	Mitochondrial carrier family	34.9	666	-0.02	0.084	-2.38	0.088	-1.74	0.013	-1.37	0.315	-0.43	0.11
Solyc05g018510	ABC transporter G family member 32	34.16	622	0.024	-0.01	-1.02	0.983	1.28	0.004	-1.30	0.105	0.59	0.021
Solyc04g009160	Unknown Protein	35.2	558	0.024	0.025	-1.40	0.088	1.17	0.245	-1.64	0.029	0.30	0.277
Solyc02g094210	Os02g0508100 protein	35.2	294	-0.02	0.001	1.01	1.000	-1.32	0.022	1.33	0.142	-0.49	0.064
Solyc02g090000	UPF0497 membrane protein At3g50810	35.2	267	-0.02	-0.02	1.31	0.086	-1.22	0.084	1.60	0.016	-0.24	0.389
Solyc06g065490	PsbP domain-containing protein 6, chloroplastic	1.1.1.2	610	0.024	-0.02	1.19	0.336	1.44	0.010	-1.20	0.246	0.71	0.003
Solyc11g007130	Major facilitator superfamily MFS_1	35.2	635	0.024	-0.01	1.05	0.686	1.31	0.012	-1.25	0.181	0.57	0.027
Solyc04g079560	Lysine/histidine transporter	34.3	424	0.024	0.017	-1.36	0.038	1.18	0.060	-1.60	0.001	0.16	0.569
Solyc08g079050	Unknown Protein	35.2	147	-0.02	0.008	1.01	0.867	-1.33	0.031	1.35	0.188	-0.47	0.077
Solyc10g085140	Undecaprenyl pyrophosphate synthase	16.1.2	24	-0.02	-0.02	1.27	0.010	-1.26	0.103	1.61	0.064	-0.35	0.201
Solyc09g065000	Xylanase inhibitor	29.5.4	269	0.024	0.016	-1.37	0.035	1.22	0.035	-1.67	0.003	0.25	0.369
Solyc03g020020	AT3G05680-like protein	33.99	638	0.024	0.016	-1.24	0.097	1.17	0.127	-1.45	0.028	0.16	0.569
Solyc12g010640	Unknown Protein	35.2	600	-0.02	-0	1.13	0.490	-1.23	0.027	1.38	0.022	-0.30	0.277
Solyc06g008730	26S protease regulatory subunit 7 homolog A	29.5.9	132	-0.02	0.044	-1.61	0.101	-1.63	0.007	1.01	0.990	-0.70	0.004
Solyc09g005550	Calcium dependent protein kinase 13	29.4	731	-0.02	0.007	-1.01	0.893	-1.33	0.004	1.32	0.019	-0.58	0.023
Solyc01g081490	RNA polymerase sigma factor	27.2	338	0.023	-0.02	1.28	0.217	1.39	0.015	-1.09	0.571	0.81	0
Solyc12g019700	Unknown Protein	35.2	703	0.023	0.011	-1.32	0.031	1.16	0.149	-1.53	0.055	0.20	0.475
Solyc01g088440	Ring H2 finger protein	35.2	134	-0.02	-0.02	1.48	0.011	-1.12	0.379	1.66	0.036	-0.11	0.696
Solyc09g010680	Dof zinc finger protein	27.3.8	454	0.023	0.014	-1.32	0.003	1.18	0.069	-1.56	0.020	0.24	0.389
Solyc10g081630	JmjC domain containing protein	27.3.57	86	0.023	-0.01	1.09	0.541	1.38	0.002	-1.26	0.207	0.76	0.001
Solyc04g079400	Protein serine/threonine kinase	29.4	584	-0.02	0.029	-1.20	0.339	-1.48	0.006	1.23	0.152	-0.58	0.023

Supplementary Table S1. continued

Solyc02g072140	UNE1-like protein	33.99	682	0.023	0.014	-1.36	0.068	1.15	0.104	-1.57	0.006	0.01	0.972
Solyc06g065440	Zinc finger family protein	27.3.11	360	0.023	-0.01	-1.09	0.345	1.34	0.008	-1.46	0.030	0.54	0.038
Solyc01g009630	Cleavage and polyadenylation specificity factor subunit 5	35.2	274	0.023	0.023	-1.57	0.023	1.17	0.275	-1.83	0.042	0.19	0.498
Solyc03g032100	Unknown Protein	35.2	318	0.023	0.005	-1.22	0.172	1.21	0.042	-1.47	0.024	0.26	0.349
Solyc04g005160	6-phosphogluconate dehydrogenase decarboxylating	7.1.3	553	-0.02	-0.02	1.45	0.024	-1.19	0.147	1.72	0.012	-0.16	0.569
Solyc10g006650	Flavoprotein wrbA	11.8	537	-0.02	-0.02	1.30	0.129	-1.16	0.187	1.50	0.026	-0.29	0.294
Solyc12g096030	Mitochondrial carrier-like protein	34.9	514	0.023	0.005	-1.20	0.075	1.22	0.017	-1.46	0.014	0.27	0.33
Solyc11g008970	Pentatricopeptide repeat-containing protein	27.3.25	99	0.023	0.016	-1.25	0.089	1.17	0.116	-1.46	0.038	0.14	0.619
Solyc07g008780	Unknown Protein	35.2	162	-0.02	-0.01	1.31	0.128	-1.22	0.074	1.59	0.011	-0.49	0.064
Solyc05g041780	Unknown Protein	35.2	77	0.023	0.062	-2.71	0.053	1.08	0.763	-2.94	0.024	-0.01	0.972
Solyc08g066790	Phospholipase D	11.9.3	484	-0.02	-0	1.22	0.036	-1.31	0.002	1.59	0.003	-0.48	0.07
Solyc02g072260	H2B histone-fold-like protein	35.2	342	-0.02	-0.01	1.22	0.294	-1.23	0.068	1.50	0.016	-0.17	0.545
Solyc04g015600	Receptor like kinase, RLK	30.2.11	88	0.023	0.027	-1.55	0.047	1.12	0.359	-1.73	0.005	-0.02	0.944
Solyc05g005190	ADP-ribosylation factor	29.3.4.99	733	-0.02	0.003	1.07	0.466	-1.26	0.029	1.36	0.099	-0.51	0.052
Solyc06g065340	Unknown Protein	35.2	551	-0.02	0.015	-1.14	0.442	-1.34	0.004	1.17	0.294	-0.58	0.023
Solyc07g015980	Lysine decarboxylase	26.13	291	0.023	0.003	-1.05	0.691	1.32	0.014	-1.39	0.046	0.41	0.129
Solyc03g095960	Pentatricopeptide repeat-containing protein	17.8.1	522	0.023	0.016	-1.30	0.055	1.15	0.064	-1.50	0.004	0.16	0.569
Solyc12g055840	Glucan endo-1 3-beta-glucosidase 7	26.4.1	734	0.023	-0.01	1.02	0.810	1.30	0.006	-1.28	0.104	0.60	0.018
Solyc03g006410	Plant-specific domain TIGR01615 family protein	35.2	111	0.023	-0.02	1.31	0.289	1.49	0.011	-1.14	0.295	0.53	0.042
Solyc01g100780	Agenet domain-containing protein	35.2	534	0.023	0.026	-1.44	0.073	1.09	0.480	-1.57	0.009	0.16	0.569
Solyc03g080090	NAC domain protein IPR003441	33.99	582	-0.02	-0.04	1.80	0.031	-1.05	0.602	1.90	0.003	-0.05	0.86
Solyc07g043510	Cysteine-type peptidase	29.5.3	379	-0.02	-0.01	1.13	0.301	-1.29	0.031	1.46	0.053	-0.49	0.064
Solyc09g056260	tRNA pseudouridine synthase family protein	29.1	336	0.023	0.02	-1.37	0.074	1.14	0.227	-1.56	0.006	-0.04	0.887
Solyc06g071900	Pentatricopeptide repeat-containing protein	27.3.67	302	0.023	0.013	-1.40	0.079	1.18	0.102	-1.65	0.000	0.14	0.619
Solyc04g071680	Receptor like kinase, RLK	30.2.11	32	-0.02	0.036	-1.45	0.111	-1.63	0.011	1.12	0.717	-0.42	0.119
Solyc03g096180	Unknown Protein	35.2	707	0.022	-0	-1.02	0.798	1.30	0.012	-1.33	0.059	0.73	0.002
Solyc02g062970	Xaa-Pro aminopeptidase 2	29.5	633	-0.02	-0.01	1.32	0.124	-1.14	0.069	1.51	0.014	-0.15	0.594
Solyc06g062460	BHLH transcription factor-like	27.3.6	253	0.022	0.035	-1.70	0.028	1.10	0.458	-1.86	0.043	0.03	0.915
Solyc01g098700	Branched-chain amino acid aminotransferase-like protein	26.26.1	695	0.022	0	-1.12	0.373	1.24	0.042	-1.39	0.070	0.49	0.064
Solyc02g085050	Inorganic pyrophosphatase-like protein	23.4.99	637	-0.02	-0.01	1.25	0.139	-1.21	0.062	1.51	0.011	-0.27	0.33
Solyc01g110310	GATA transcription factor 9	27.3.9	459	0.022	-0.01	1.06	0.632	1.33	0.005	-1.25	0.124	0.58	0.023
Solyc02g084550	Chromosome 10 contig 1 DNA sequence	35.2	112	-0.02	0.014	-1.01	0.917	-1.39	0.010	1.38	0.094	-0.63	0.012
Solyc02g070260	Protein phosphatase 1 regulatory subunit 7	35.2	592	0.022	0.007	-1.13	0.386	1.25	0.039	-1.41	0.031	0.57	0.027
Solyc04g074870	Purine permease family protein	34.10	521	0.022	-0.01	-1.18	0.410	1.30	0.015	-1.53	0.052	0.29	0.294
Solyc02g093310	MAP-like protein kinase	30.6	738	-0.02	0.007	1.10	0.502	-1.35	0.004	1.48	0.008	-0.43	0.11
Solyc02g088820	Serine carboxypeptidase K10B2.2	29.5.5	18	0.022	-0.07	2.08	0.030	1.86	0.006	1.12	0.754	0.53	0.042
Solyc10g055350	Unknown Protein	35.2	747	0.022	0.013	-1.21	0.234	1.16	0.102	-1.41	0.016	0.28	0.312
Solyc02g063020	Major facilitator superfamily MFS_1	34.99	282	-0.02	0.011	1.02	0.740	-1.36	0.010	1.39	0.114	-0.52	0.047
Solyc09g082460	Homocysteine s-methyltransferase	13.1.3.4	62	0.022	-0	-1.23	0.275	1.40	0.013	-1.72	0.028	0.34	0.215

Supplementary Table S1. continued

Solyc12g008520	Calmodulin-binding family protein	30.3	380	0.022	0.003	-1.17	0.349	1.29	0.021	-1.51	0.015	0.18	0.521
Solyc08g079750	1-aminocyclopropane-1-carboxylate synthase	17.5.1.1	367	0.022	0.016	-1.32	0.043	1.20	0.024	-1.59	0.001	0.30	0.277
Solyc09g007750	Receptor-like kinase	29.4.1.57	533	-0.02	-0.02	1.60	0.089	-1.16	0.287	1.85	0.011	0.06	0.832
Solyc09g089580	1-aminocyclopropane-1-carboxylate oxidase-like protein	17.5.1	524	0.022	0.008	-1.26	0.145	1.20	0.045	-1.52	0.065	0.17	0.545
Solyc10g078700	Squamosa promoter-binding protein	27.3.28	743	0.022	-0.01	-1.01	0.914	1.34	0.011	-1.36	0.019	0.23	0.41
Solyc03g083120	Unknown Protein	35.2	321	0.022	0.073	-2.65	0.012	-1.16	0.496	-2.29	0.014	-0.18	0.521
Solyc12g014020	SOSS complex subunit B1	35.2	325	0.021	0.022	-1.46	0.041	1.06	0.615	-1.54	0.015	-0.07	0.804
Solyc09g063030	CLIP-associating protein 1-like	35.2	681	0.021	0.015	-1.27	0.136	1.14	0.168	-1.45	0.009	0.23	0.41
Solyc09g083330	Phosphatidate cytidylyltransferase	11.3	255	-0.02	0.038	-1.44	0.151	-1.56	0.021	1.08	0.827	-0.60	0.018
Solyc07g049300	Endoglucanase 1	10.6.1	556	-0.02	0.001	1.10	0.485	-1.24	0.017	1.37	0.037	-0.35	0.201
Solyc01g106800	Amino acid permease 6	34.3	580	-0.02	-0.04	1.64	0.049	-1.00	0.938	1.65	0.015	0.07	0.804
Solyc12g049510	Erect panical 2	33.99	456	-0.02	-0.03	1.74	0.012	-1.07	0.412	1.86	0.002	-0.15	0.594
Solyc12g099650	Photosystem II 5 kDa protein, chloroplastic	35.2	670	-0.02	-0.03	1.52	0.083	-1.04	0.663	1.58	0.008	0.04	0.887
Solyc01g108580	Gibberellin receptor GID1L2	24	680	-0.02	-0.01	1.19	0.307	-1.15	0.096	1.38	0.014	-0.27	0.33
Solyc05g053880	cDNA FLJ42396 fis clone ASTRO2001107	35.2	107	0.021	0.03	-1.63	0.049	1.03	0.808	-1.69	0.025	-0.18	0.521
Solyc00g144470	Serine/threonine-protein phosphatase 7 long form homolog	29.4	432	0.021	0.053	-1.98	0.042	1.02	0.967	-2.02	0.019	-0.13	0.644
Solyc04g077750	Unknown Protein	35.2	505	0.021	-0.07	2.29	0.001	1.60	0.035	1.43	0.680	0.64	0.01
Solyc09g090620	CC-NBS-LRR class disease resistance protein	35.2	174	0.021	-0.03	1.44	0.175	1.69	0.006	-1.18	0.516	0.70	0.004
Solyc03g059190	(+)-neomenthol dehydrogenase	26.22	181	0.021	-0.04	1.78	0.063	1.72	0.004	1.03	0.902	0.79	0
Solyc11g012470	Heterogeneous nuclear ribonucleoprotein A3	27.4	597	0.021	-0.01	-1.01	0.941	1.26	0.022	-1.27	0.144	0.55	0.034
Solyc03g025560	Undecaprenyl pyrophosphate synthase	16.1.5	481	0.021	0.014	-1.29	0.056	1.12	0.196	-1.44	0.025	0.30	0.277
Solyc04g077290	YEATS domain-containing protein	27.3.51	617	0.021	0.005	-1.11	0.390	1.19	0.027	-1.32	0.066	0.39	0.151
Solyc10g086470	Chromosome 05 contig 1 DNA sequence	35.2	244	-0.02	0.024	-1.43	0.241	-1.59	0.013	1.11	0.633	-0.51	0.052
Solyc05g013530	Octicosapeptide/Phox/Bem1p domain-containing protein	29.4	509	0.021	-0.03	1.29	0.113	1.54	0.000	-1.19	0.100	0.69	0.004
Solyc05g021370	Unknown Protein	35.2	146	0.021	-0.1	3.47	0.150	2.85	0.027	1.22	0.460	0.27	0.33
Solyc01g017860	Unknown Protein	35.2	170	0.02	0.01	-1.49	0.024	1.21	0.073	-1.80	0.008	0.13	0.644
Solyc10g007950	Alpha alpha-trehalose-phosphate synthase	3.2.1	479	0.02	0.009	-1.30	0.045	1.15	0.167	-1.49	0.040	0.11	0.696
Solyc03g112420	Pentatricopeptide repeat-containing protein	29.4	281	0.02	-0	1.01	0.948	1.29	0.010	-1.28	0.095	0.43	0.11
Solyc01g104950	Alpha-L-arabinofuranosidase/beta-D-xylosidase	10.6.2	748	0.02	-0.02	1.13	0.242	1.35	0.004	-1.19	0.263	0.56	0.03
Solyc00g036740	Unknown Protein	35.2	85	-0.02	-0.02	1.36	0.106	-1.19	0.186	1.61	0.031	-0.08	0.777
Solyc02g093350	Homology to unknown gene	35.2	415	-0.02	0.01	1.03	0.913	-1.28	0.009	1.32	0.015	-0.35	0.201
Solyc02g083590	Dehydroquinate synthase	13.1.6.1.2	688	-0.02	-0.01	1.36	0.104	-1.11	0.253	1.50	0.013	0.02	0.944
Solyc12g056800	Oxidoreductase family protein	35.2	726	-0.02	0.011	-1.02	0.996	-1.28	0.006	1.25	0.109	-0.37	0.175
Solyc09g061320	Solute carrier family 35 member F4	35.2	692	-0.02	-0	1.10	0.452	-1.31	0.021	1.44	0.054	-0.38	0.162
Solyc05g045670	Glucose-6-phosphate/phosphate translocator 2	34.8	343	0.02	0.159	-14.65	0.024	-2.08	0.363	-7.05	0.095	-0.14	0.619
Solyc01g088170	Aldehyde oxidase	17.1.1	696	0.02	-0.01	1.20	0.412	1.36	0.015	-1.14	0.165	0.77	0.001
Solyc11g008620	Phosphoglycolate phosphatase	1.2.1	651	-0.02	-0.02	1.37	0.081	-1.12	0.281	1.54	0.019	-0.19	0.498
Solyc06g065230	N-acetyltransferase 5	27.3.70	387	0.02	0.004	-1.13	0.301	1.24	0.037	-1.40	0.056	0.32	0.245
Solyc00g136260	Ring H2 finger protein	29.5.11.4.2	714	0.02	-0.02	1.22	0.309	1.44	0.007	-1.18	0.231	0.87	0

Supplementary Table S1. continued

Solyc11g008680	Fatty acid desaturase, type 2	11.1.15	185	-0.02	-0.03	1.48	0.080	-1.15	0.248	1.71	0.014	-0.24	0.389
Solyc09g055700	Myosin-like protein	35.2	517	0.02	0.012	-1.37	0.013	1.13	0.062	-1.55	0.013	0.10	0.723
Solyc05g015730	MADS-box transcription factor 1	27.3.24	353	0.02	0.022	-1.35	0.116	1.11	0.370	-1.50	0.003	0.30	0.277
Solyc00g133710	Unknown Protein	35.2	265	-0.02	-0.03	1.76	0.001	-1.05	0.770	1.84	0.008	-0.06	0.832
Solyc06g033930	Polyprotein	35.2	37	-0.02	0.061	-1.85	0.110	-1.71	0.029	-1.08	0.674	-0.47	0.077
Solyc01g091540	Growth-regulating factor 12	27.3.50	450	-0.02	0.027	-1.29	0.281	-1.46	0.022	1.13	0.518	-0.66	0.007
Solyc07g025400	Unknown Protein	35.2	444	0.02	-0.01	1.18	0.310	1.36	0.016	-1.15	0.349	0.77	0.001
Solyc08g081900	Translation initiation factor IF-2	29.2.3	519	0.019	0.024	-1.44	0.010	1.07	0.384	-1.54	0.015	-0.02	0.944
Solyc03g031430	Urease accessory protein UreF	12.4	263	-0.02	0.02	-1.29	0.206	-1.45	0.018	1.12	0.596	-0.45	0.092
Solyc03g114020	Genomic DNA chromosome 5 P1 clone MQB2	35.2	653	0.019	-0.01	-1.02	0.800	1.27	0.013	-1.30	0.037	0.57	0.027
Solyc09g074360	RNA binding protein	27.4	603	0.019	0.013	-1.31	0.074	1.12	0.072	-1.46	0.013	0.09	0.75
Solyc11g020720	MRNA clone RAFL21-57-I05	35.2	235	0.019	-0.01	-1.04	0.697	1.32	0.012	-1.37	0.024	0.61	0.016
Solyc06g071920	Glyceraldehyde-3-phosphate dehydrogenase	4.1.8	698	-0.02	-0.01	1.12	0.474	-1.25	0.029	1.41	0.054	-0.41	0.129
Solyc04g078290	Cytochrome P450	26.10	159	-0.02	-0.02	1.39	0.040	-1.15	0.071	1.60	0.008	-0.23	0.41
Solyc08g015730	F-box family protein	29.5.11.4.3.2	35	0.019	0.048	-2.62	0.018	1.03	0.666	-2.70	0.032	-0.08	0.777
Solyc08g005540	Amino acid permease-like protein	34.3	319	0.019	0.021	-1.46	0.033	1.11	0.305	-1.63	0.007	0.06	0.832
Solyc01g010650	UDP-galactose transporter 3	34.11	540	-0.02	0.001	1.14	0.267	-1.18	0.025	1.34	0.061	-0.25	0.369
Solyc03g051810	Homogentisate phytyltransferase	16.1.3	557	0.018	-0.02	1.16	0.311	1.38	0.001	-1.19	0.103	0.52	0.047
Solyc00g041210	Tetratricopeptide TPR_2 repeat protein	35.2	135	-0.02	-0.02	1.51	0.007	-1.07	0.619	1.62	0.030	-0.06	0.832
Solyc04g080720	Transferase family protein	16.2	326	0.018	0.079	-2.62	0.026	-1.12	0.567	-2.35	0.012	-0.28	0.312
Solyc07g018180	Receptor like kinase, RLK	30.2.11	121	0.018	0.047	-2.09	0.033	-1.04	0.669	-2.01	0.005	-0.29	0.294
Solyc02g092810	RNA-binding protein MEX3C	35.2	74	-0.02	0.017	-1.07	0.708	-1.30	0.009	1.21	0.174	-0.64	0.01
Solyc01g080010	Xylanase inhibitor	29.5.4	129	0.018	-0.06	2.31	0.004	1.62	0.008	1.43	0.182	0.45	0.092
Solyc11g006540	FAD-dependent pyridine nucleotide-disulphide oxidoreductase	35.2	175	-0.02	0.005	1.06	0.769	-1.25	0.033	1.33	0.042	-0.61	0.016
Solyc12g056350	Unknown Protein	35.2	446	0.018	0.03	-1.68	0.027	1.04	0.799	-1.74	0.013	-0.01	0.972
Solyc00g007060	Serine/threonine kinase-like protein ABC1063	29.4.1.57	392	-0.02	-0	1.11	0.483	-1.21	0.048	1.34	0.030	-0.51	0.052
Solyc07g065860	Receptor like kinase, RLK	30.2.11	268	0.018	-0.05	1.79	0.065	1.66	0.011	1.08	0.819	0.71	0.003
Solyc02g080900	Genomic DNA chromosome 5 TAC clone K6A12	35.2	589	-0.02	-0.02	1.46	0.041	-1.17	0.116	1.70	0.007	-0.06	0.832
Solyc02g068090	30S ribosomal protein S21, chloroplastic	29.2.1.1.3.1.21	376	0.018	-0.03	1.25	0.110	1.45	0.002	-1.16	0.280	0.75	0.001
Solyc01g081640	Legume lectin beta domain	35.2	402	0.018	0.013	-1.28	0.103	1.20	0.081	-1.54	0.014	0.36	0.187
Solyc06g050340	Unknown Protein	35.2	315	0.018	0.136	-6.22	0.004	-2.39	0.084	-2.61	0.089	-0.30	0.277
Solyc01g110550	Ribosome-binding factor A	27.1	352	0.018	0.012	-1.28	0.024	1.14	0.151	-1.47	0.033	0.25	0.369
Solyc07g025250	Pentatricopeptide repeat-containing protein	17.8.1	354	0.018	0.016	-1.36	0.023	1.08	0.247	-1.46	0.016	0.05	0.86
Solyc08g078950	Nitrate transporter	34.13	276	0.018	-0	-1.05	0.660	1.26	0.026	-1.33	0.046	0.31	0.261
Solyc03g120850	chaperonin	29.6	709	0.018	-0.03	1.38	0.115	1.33	0.008	1.04	0.662	0.44	0.101
Solyc05g042040	UBX domain-containing protein	29.5	410	0.017	0.004	-1.16	0.146	1.18	0.046	-1.37	0.052	0.22	0.431
Solyc06g007250	Leucine-rich repeat family protein	20.1	636	0.017	0.006	-1.17	0.090	1.19	0.047	-1.39	0.039	0.39	0.151
Solyc07g043160	UDP-glucosyltransferase	26.2	259	0.017	-0.02	1.26	0.090	1.34	0.012	-1.07	0.644	0.63	0.012
Solyc01g006690	Unknown Protein	35.2	306	0.017	0.002	-1.12	0.471	1.27	0.016	-1.43	0.096	0.40	0.14
Solyc01g108300	Myb family transcription factor	27.3.20	539	0.017	-0.06	1.92	0.015	1.59	0.000	1.21	0.287	0.77	0.001
Solyc03g120190	Plant-specific domain TIGR01568 family protein	35.2	23	-0.02	-0.01	1.33	0.127	-1.09	0.272	1.45	0.009	-0.10	0.723

Supplementary Table S1. continued

Solyc11g021360	Unknown Protein	35.2	737	0.017	0.035	-1.66	0.043	1.05	0.811	-1.74	0.010	0.15	0.594
Solyc05g054680	Pto-like, Serine/threonine kinase protein, resistance protein	29.4.1.57	223	0.017	0.01	-1.28	0.065	1.11	0.232	-1.43	0.016	0.26	0.349
Solyc02g062950	G-box binding factor	27.3.35	668	0.017	0.007	-1.17	0.283	1.20	0.074	-1.40	0.011	0.39	0.151
Solyc02g088430	ATP-dependent DNA helicase RecQ	28.99	110	0.017	0.037	-1.95	0.010	1.00	0.991	-1.95	0.008	-0.26	0.349
Solyc02g089060	Hydrolase alpha/beta fold family protein	26.8	208	-0.02	0.05	-1.69	0.165	-1.47	0.008	-1.15	0.772	-0.51	0.052
Solyc01g107170	Zinc finger protein	27.3.11	196	-0.02	-0.02	1.28	0.010	-1.14	0.201	1.46	0.049	-0.18	0.521
Solyc12g098850	Soluble diacylglycerol acyltransferase	35.2	341	0.017	0.07	-3.16	0.019	-1.10	0.824	-2.88	0.033	-0.23	0.41
Solyc10g008450	F-box family protein	29.5.11.4.3.2	279	0.017	0.012	-1.26	0.038	1.08	0.318	-1.36	0.038	0.08	0.777
Solyc06g009290	Lipid A export ATP-binding/permease protein msbA	34.16	57	-0.02	-0.06	2.78	0.023	1.22	0.456	2.28	0.003	0.26	0.349
Solyc11g012540	F-box family protein	29.5.11.4.3.2	213	0.017	0.038	-1.65	0.022	-1.02	0.917	-1.62	0.032	-0.08	0.777
Solyc01g079310	Unknown Protein	35.2	395	-0.02	-0.02	1.43	0.039	-1.16	0.100	1.66	0.002	-0.23	0.41
Solyc02g083520	BZIP transcription factor	35.2	6	0.017	-0	-1.05	0.767	1.29	0.009	-1.36	0.087	0.53	0.042
Solyc12g049320	GRAS family transcription factor	27.3.21	271	-0.02	0.077	-2.01	0.030	-1.86	0.034	-1.08	0.676	-0.50	0.058
Solyc11g066620	Aspartyl protease family protein	29.5.4	629	-0.02	0.026	-1.29	0.042	-1.36	0.012	1.06	0.814	-0.55	0.034
Solyc05g051970	cDNA clone J033025P19 full insert sequence	35.2	240	-0.02	0.035	-1.47	0.037	-1.46	0.018	-1.01	0.909	-0.60	0.018
Solyc11g064950	BZIP transcription factor	27.3.35	433	-0.02	0.033	-1.44	0.082	-1.48	0.009	1.03	0.890	-0.60	0.018
Solyc02g068990	Unknown Protein	35.2	283	0.016	0.01	-1.26	0.013	1.17	0.030	-1.48	0.017	0.18	0.521
Solyc07g008030	Glycosyl transferase family 17 protein	26.8	404	-0.02	0.018	-1.22	0.098	-1.31	0.011	1.08	0.656	-0.55	0.034
Solyc12g077660	Set protein	35.2	486	0.016	-0.09	4.61	0.005	1.74	0.087	2.65	0.202	0.66	0.007
Solyc09g007850	RNA-binding protein	27.4	742	0.016	-0.02	1.25	0.150	1.31	0.009	-1.05	0.666	0.66	0.007
Solyc00g055950	Response regulator receiver Transcriptional regulatory protein	35.2	66	-0.02	0.03	-1.59	0.028	-1.36	0.056	-1.17	0.395	-0.49	0.064
Solyc01g022740	Kelch-like protein 21	29.5.11.4.3.2	251	0.016	0.019	-1.40	0.055	1.07	0.513	-1.49	0.031	-0.17	0.545
Solyc08g007300	Cyclin-D-binding Myb-like transcription factor 1	27.3.25	228	0.016	0.023	-1.47	0.013	1.01	0.851	-1.49	0.024	-0.22	0.431
Solyc05g005180	Naphthoate synthase	18.5.2.5	520	0.016	-0.05	1.70	0.040	1.50	0.001	1.13	0.350	0.74	0.002
Solyc06g050330	Unknown Protein	35.2	500	-0.02	0.213	-11.51	0.007	-4.24	0.015	-2.72	0.072	-0.36	0.187
Solyc04g007700	Unknown Protein	35.2	570	-0.02	0.014	-1.09	0.318	-1.27	0.004	1.16	0.154	-0.68	0.005
Solyc10g074980	Vacuolar sorting receptor	29.3.4.3	711	-0.02	-0.02	1.40	0.049	-1.07	0.434	1.50	0.012	0.06	0.832
Solyc02g078590	Alpha-1 4-galactosyltransferase	26.2	464	0.016	-0.03	1.23	0.079	1.32	0.010	-1.08	0.554	0.54	0.038
Solyc02g091910	EPIDERMAL PATTERNING FACTOR-like protein 2	35.2	41	0.016	0.047	-2.16	0.001	-1.15	0.230	-1.88	0.004	-0.22	0.431
Solyc02g093790	CXE carboxylesterase	24	327	0.016	-0.01	1.11	0.276	1.31	0.009	-1.18	0.253	0.60	0.018
Solyc00g034000	Permease of the major facilitator superfamily	35.2	183	-0.02	0.046	-1.65	0.050	-1.50	0.015	-1.10	0.603	-0.49	0.064
Solyc07g024000	Dehydrogenase/ reductase 3	26.22	467	0.015	0.034	-1.57	0.039	1.05	0.698	-1.65	0.017	-0.09	0.75
Solyc02g064600	Unknown Protein	35.2	357	0.015	0.016	-1.29	0.016	1.09	0.267	-1.40	0.044	0.21	0.453
Solyc01g109930	Inositol monophosphatase family protein	3.4.5	465	-0.02	-0.02	1.44	0.031	-1.08	0.345	1.56	0.009	0.03	0.915
Solyc03g006590	Unknown Protein	35.2	647	-0.02	0.027	-1.35	0.118	-1.42	0.011	1.05	0.802	-0.71	0.003
Solyc05g053960	Amino acid transporter	35.2	124	-0.02	0.104	-3.74	0.009	-2.35	0.043	-1.59	0.290	-0.45	0.092
Solyc06g076790	Unknown Protein	35.2	573	-0.02	-0.04	1.75	0.040	1.06	0.737	1.65	0.024	0.29	0.294
Solyc03g005060	Os12g0236050 protein	28.1	478	-0.02	0.018	-1.18	0.152	-1.29	0.020	1.10	0.582	-0.69	0.004
Solyc11g012410	Inositol monophosphatase 3	3.4.5	528	-0.02	-0.02	1.46	0.041	-1.06	0.578	1.55	0.023	-0.18	0.521

Supplementary Table S1. continued

Solyc09g063070	Senescence-associated protein-like	33.99	101	0.015	0.016	-1.36	0.044	1.13	0.212	-1.54	0.011	0.11	0.696
Solyc08g077950	Unknown Protein	35.2	292	0.015	-0	-1.18	0.175	1.22	0.035	-1.44	0.028	0.32	0.245
Solyc04g009550	FGFR1 oncogene partner	31.1	167	-0.01	0.025	-1.24	0.267	-1.35	0.018	1.09	0.520	-0.71	0.003
Solyc03g082890	Pentapeptide repeat protein	35.2	362	0.014	-0.02	1.16	0.291	1.29	0.018	-1.11	0.417	0.64	0.01
Solyc05g050840	Unknown Protein	35.2	331	0.014	0.031	-1.63	0.019	-1.08	0.388	-1.51	0.027	-0.32	0.245
Solyc06g034290	Glycerol-3-phosphate transporter	34.98	483	0.014	0.026	-1.56	0.024	1.01	0.892	-1.58	0.028	-0.12	0.67
Solyc03g005670	Cc-nbs-lrr, resistance protein	20.1	29	-0.01	0.015	-1.11	0.431	-1.34	0.008	1.21	0.173	-0.51	0.052
Solyc10g054870	Triosephosphate isomerase	1.3.5	156	-0.01	0.058	-2.18	0.024	-1.84	0.040	-1.18	0.483	-0.51	0.052
Solyc03g110920	Receptor expression-enhancing protein 5	17.1.3	246	-0.01	0.021	-1.30	0.007	-1.31	0.007	1.01	0.957	-0.63	0.012
Solyc01g079150	AE family transporter anion exchange	34.18	675	-0.01	0.019	-1.27	0.215	-1.32	0.015	1.04	0.732	-0.47	0.077
Solyc08g013770	F-box family protein	35.2	280	0.013	0.148	-6.64	0.000	-3.85	0.060	-1.73	0.247	-0.40	0.14
Solyc03g026020	Heat stress transcription factor	20.2.1	418	0.013	0.025	-1.56	0.029	1.08	0.321	-1.68	0.011	-0.03	0.915
Solyc02g093270	Caffeoyl-CoA O-methyltransferase	16.2.1.6	90	-0.01	-0.05	1.98	0.032	1.23	0.235	1.61	0.042	0.23	0.41
Solyc06g050170	Potassium transporter	34.15	684	0.013	0.011	-1.28	0.052	1.12	0.092	-1.44	0.028	0.16	0.569
Solyc08g005800	Pectinacetylesterase like protein	10.8.2	730	0.013	-0.05	1.87	0.003	1.42	0.009	1.32	0.255	0.66	0.007
Solyc01g091770	Ring H2 finger protein	29.5.11.4.2	28	0.013	-0.02	1.09	0.467	1.29	0.016	-1.19	0.229	0.65	0.009
Solyc10g039280	Dual specificity protein phosphatase family protein	35.2	482	-0.01	-0.03	1.59	0.003	1.00	0.944	1.58	0.006	0.20	0.475
Solyc10g018850	Dienelactone hydrolase family protein	26.1	494	0.013	0.025	-1.41	0.075	1.03	0.809	-1.46	0.014	-0.05	0.86
Solyc09g089490	Proteinase inhibitor I	35.2	20	0.013	0.044	-2.39	0.111	1.07	0.647	-2.56	0.045	-0.08	0.777
Solyc09g007370	Ribonuclease P protein subunit p29	27.1.19	22	0.013	-0.04	1.68	0.087	1.49	0.026	1.13	0.570	0.62	0.014
Solyc07g042540	Unknown Protein	35.2	168	0.012	0.045	-2.18	0.001	-1.21	0.340	-1.81	0.039	-0.34	0.215
Solyc11g020590	Peptide chain release factor-like protein	29.2.5	359	0.012	0.018	-1.49	0.031	1.08	0.460	-1.61	0.047	-0.06	0.832
Solyc06g005290	UDP-galactose transporter 5	34.11	451	0.012	0.018	-1.36	0.009	1.06	0.447	-1.44	0.061	-0.06	0.832
Solyc06g007720	Unknown Protein	35.2	25	0.012	0.032	-1.58	0.007	-1.05	0.530	-1.51	0.002	-0.24	0.389
Solyc01g066880	Copper chaperone	15.2	65	0.012	-0.01	1.03	0.863	1.25	0.020	-1.21	0.073	0.71	0.003
Solyc08g041800	Unknown Protein	35.2	382	0.012	0.028	-1.52	0.007	-1.05	0.733	-1.45	0.043	-0.15	0.594
Solyc10g055800	Chitinase	20.1	206	0.012	-0.1	3.08	0.100	2.54	0.022	1.21	0.724	0.73	0.002
Solyc09g055710	Unknown Protein	35.2	461	0.012	0.036	-1.70	0.002	-1.08	0.542	-1.57	0.022	-0.11	0.696
Solyc07g008430	Unknown Protein	35.2	717	0.011	0.015	-1.25	0.026	1.09	0.209	-1.37	0.040	0.05	0.86
Solyc01g109430	PPPDE peptidase domain-containing protein 1	17.5.2	457	-0.01	0.027	-1.40	0.124	-1.38	0.013	-1.02	0.815	-0.62	0.014
Solyc02g093950	Ankyrin repeat family protein	31.1	344	0.011	0.033	-1.64	0.040	-1.05	0.527	-1.56	0.006	-0.40	0.14
Solyc10g086580	Ribulose-1 5-bisphosphate carboxylase/oxygenase activase 1	1.3.13	627	-0.01	-0.07	2.39	0.048	1.37	0.183	1.74	0.010	0.41	0.129
Solyc05g047610	DHHC zinc finger domain containing protein expressed	27.3.99	68	-0.01	-0.06	2.23	0.009	1.18	0.305	1.90	0.010	0.35	0.201
Solyc05g024260	RAG1-activating protein 1 homolog	33.99	388	0.011	0.054	-2.05	0.007	-1.08	0.784	-1.90	0.036	-0.23	0.41
Solyc06g062400	Chloroplast unusual positioning 1A	31.1	48	0.011	0.022	-1.59	0.059	-1.01	0.800	-1.56	0.019	-0.16	0.569
Solyc06g053980	Chlorophyllase 2	19.99	119	-0.01	-0.04	1.69	0.015	-1.03	0.985	1.73	0.046	0.03	0.915
Solyc04g081510	Os01g0873900 protein	35.2	599	-0.01	0.018	-1.08	0.493	-1.29	0.020	1.19	0.253	-0.44	0.101
Solyc02g088150	Pentatricopeptide repeat-containing protein	17.8.1	50	0.01	0.053	-2.17	0.014	-1.21	0.325	-1.80	0.066	-0.30	0.277
Solyc06g069780	E3 ubiquitin-protein ligase MARCH3	29.5.11.4.2	572	-0.01	-0.02	1.42	0.065	-1.03	0.633	1.47	0.005	-0.15	0.594

Supplementary Table S1. continued

Solyc06g005050	F-box/FBD/LRR-repeat protein At1g13570	29.5.11.4.3.2	662	0.01	0.026	-1.46	0.005	-1.05	0.684	-1.39	0.072	-0.12	0.67
Solyc03g117960	Tyrosyl-DNA phosphodiesterase family protein	27.3.48	298	-0.01	0.045	-1.66	0.011	-1.40	0.011	-1.19	0.357	-0.60	0.018
Solyc06g007760	Ycf54 protein	35.2	575	-0.01	-0.04	1.61	0.042	1.12	0.382	1.44	0.033	0.21	0.453
Solyc03g121970	Unknown Protein	35.2	544	-0.01	0.026	-1.39	0.047	-1.28	0.002	-1.09	0.545	-0.66	0.007
Solyc09g025270	U-box domain-containing protein 3	29.5.11.4.2	355	0.01	0.017	-1.37	0.031	1.03	0.703	-1.41	0.031	-0.08	0.777
Solyc00g272810	N-acetyltransferase	26.24	27	0.01	-0.05	1.88	0.143	1.69	0.023	1.12	0.191	0.61	0.016
Solyc02g084290	Unknown Protein	35.2	460	0.01	0.036	-1.82	0.012	-1.12	0.365	-1.63	0.020	-0.29	0.294
Solyc01g080900	Cytochrome P450	26.10	17	-0.01	-0.03	1.77	0.014	1.13	0.320	1.56	0.065	0.45	0.092
Solyc10g055810	Endochitinase	20.1	586	-0.01	-0.17	6.59	0.024	1.84	0.149	3.58	0.150	0.53	0.042
Solyc08g075990	Os02g0129300 protein	35.2	226	0.009	0.019	-1.47	0.012	-1.01	0.947	-1.45	0.025	-0.26	0.349
Solyc05g007670	Ectonucleoside triphosphate diphosphohydrolase 1	23.2	462	-0.01	-0.02	1.37	0.026	-1.05	0.564	1.44	0.035	-0.04	0.887
Solyc11g007690	Pyruvate kinase	11.1.30	476	0.009	-0.01	1.09	0.583	1.28	0.012	-1.18	0.184	0.48	0.07
Solyc11g067270	Acyltransferase-like protein	16.2	243	0.008	-0.12	4.07	0.048	2.25	0.047	1.81	0.259	0.29	0.294
Solyc05g015500	Zinc finger Ran-binding domain-containing protein 2	27.3.99	639	0.008	0.039	-1.65	0.016	-1.08	0.440	-1.53	0.013	-0.29	0.294
Solyc09g091920	Glucan endo-1 3-beta-glucosidase 4	26.4	79	-0.01	-0.02	1.49	0.039	1.00	0.992	1.49	0.028	-0.01	0.972
Solyc04g064740	Os06g0207500 protein	10.8.1	285	-0.01	-0.12	5.40	0.009	1.37	0.252	3.96	0.116	0.21	0.453
Solyc03g121250	3&apos-5&apos exonuclease domain-containing protein-like	28.99	386	-0.01	0.034	-1.60	0.028	-1.31	0.025	-1.22	0.191	-0.66	0.007
Solyc12g005880	Beta-1 3-galactosyltransferase 6	29.7	139	0.008	0.053	-1.86	0.021	-1.19	0.256	-1.56	0.068	-0.25	0.369
Solyc03g119540	CONSTANS-like zinc finger protein	27.3.7	229	0.008	-0.09	3.52	0.006	1.61	0.061	2.19	0.084	0.43	0.11
Solyc04g072410	Trafficking protein particle complex subunit 6B	35.2	620	-0.01	-0.02	1.42	0.042	1.02	0.871	1.39	0.008	-0.01	0.972
Solyc11g066520	Serine carboxypeptidase family protein expressed	29.5.5	39	-0.01	-0.04	1.90	0.004	1.11	0.249	1.70	0.013	0.18	0.521
Solyc05g007650	Unknown protein	35.2	91	0.007	0.076	-3.60	0.022	-1.32	0.215	-2.72	0.054	-0.22	0.431
Solyc08g059700	Genomic DNA chromosome 3 P1 clone MUJ8	35.2	393	0.007	0.127	-4.38	0.013	-1.63	0.027	-2.70	0.001	-0.37	0.175
Solyc02g065040	Unknown Protein	35.2	179	-0.01	0.08	-2.78	0.016	-1.50	0.027	-1.85	0.131	-0.37	0.175
Solyc05g052470	Ferritin	15.2	299	0.007	-0.04	1.80	0.020	1.30	0.067	1.38	0.162	0.46	0.084
Solyc08g016500	Potassium channel	34.15	10	0.007	0.057	-2.51	0.048	-1.08	0.649	-2.32	0.043	-0.11	0.696
Solyc11g066750	AP-2 complex subunit alpha-1	31.4	659	-0.01	0.033	-1.57	0.064	-1.26	0.022	-1.25	0.222	-0.58	0.023
Solyc09g082390	Pentatricopeptide repeat-containing protein	33.99	216	0.006	0.027	-1.50	0.027	-1.10	0.117	-1.36	0.010	-0.31	0.261
Solyc02g069450	Photosystem I reaction center subunit III	1.1.2.2	272	0.006	-0.05	1.87	0.102	1.58	0.030	1.18	0.274	0.75	0.001
Solyc02g083690	Senescence-associated protein 12	33.99	56	0.006	0.045	-2.00	0.020	-1.27	0.175	-1.58	0.062	-0.41	0.129
Solyc02g086160	D-xylose transporter	34.2	541	0.006	-0.05	1.82	0.007	1.26	0.102	1.44	0.172	0.43	0.11
Solyc03g115270	Expansin	10.7	122	-0.01	0.03	-1.45	0.016	-1.35	0.025	-1.07	0.625	-0.54	0.038
Solyc01g099260	Chaperone protein dnaJ	35.2	80	-0.01	-0.05	1.80	0.038	1.25	0.136	1.44	0.044	0.43	0.11
Solyc02g088210	Xenotropic and polytropic retrovirus receptor	20.2	231	-0.01	0.057	-1.92	0.010	-1.48	0.010	-1.30	0.131	-0.62	0.014
Solyc00g050130	UDP-glucose glucosyltransferase	26.2	333	0.005	0.033	-1.53	0.019	-1.15	0.159	-1.33	0.052	-0.54	0.038
Solyc06g083680	Photosystem I reaction center subunit IV A	1.1.2.2	721	0.005	-0.04	1.49	0.077	1.24	0.037	1.21	0.216	0.34	0.215
Solyc02g022930	3-hydroxyisobutyrate dehydrogenase-like protein	7.1.3	103	-0.01	0.04	-1.80	0.013	-1.31	0.088	-1.38	0.114	-0.50	0.058
Solyc08g078300	Homeobox-leucine zipper protein	27.3.22	469	-0.01	0.055	-2.02	0.021	-1.36	0.038	-1.48	0.052	-0.60	0.018

Supplementary Table S1. continued

Solyc06g048590	Transmembrane protein	35.2	328	-0	-0.05	2.13	0.015	1.25	0.223	1.71	0.117	0.35	0.201
Solyc08g005600	Unknown Protein	35.2	340	0.004	-0.06	2.22	0.000	1.36	0.015	1.63	0.035	0.58	0.023
Solyc05g007300	Receptor expression-enhancing protein 5	17.1.3	198	0.004	0.054	-2.74	0.003	-1.23	0.297	-2.23	0.014	-0.31	0.261
Solyc01g105100	Endoplasmic reticulum-Golgi intermediate compartment protein 3	21.1	356	0.004	-0.02	1.44	0.019	1.15	0.124	1.25	0.180	0.35	0.201
Solyc12g005410	Bile acid sodium symporter family protein	34.14	324	-0	0.046	-1.81	0.038	-1.29	0.049	-1.40	0.033	-0.32	0.245
Solyc03g095810	Os03g0291800 protein	31.1	114	-0	0.054	-2.73	0.037	-1.29	0.184	-2.12	0.090	-0.21	0.453
Solyc04g071080	Unknown Protein	35.2	739	0.004	0.037	-1.79	0.011	-1.22	0.103	-1.47	0.040	-0.30	0.277
Solyc03g082510	Auxin-responsive family protein	17.2.3	257	0.003	0.024	-1.37	0.046	-1.13	0.131	-1.22	0.103	-0.48	0.07
Solyc06g084240	Ent-copalyl diphosphate synthase	16.1.5	33	0.002	0.06	-2.31	0.004	-1.37	0.128	-1.68	0.085	-0.36	0.187
Solyc04g040000	Unknown Protein	35.2	164	-0	0.031	-1.47	0.022	-1.34	0.024	-1.10	0.572	-0.51	0.052
Solyc00g007030	Glutathione S-transferase	26.9	7	0.002	-0.04	1.67	0.056	1.35	0.042	1.24	0.208	0.59	0.021
Solyc11g065820	Mate efflux family protein	34.99	19	0.002	0.065	-2.64	0.014	-1.38	0.183	-1.92	0.140	-0.29	0.294
Solyc05g005210	Baculoviral IAP repeat-containing 2	29.5.11.4.2	145	-0	0.027	-1.55	0.037	-1.16	0.089	-1.33	0.132	-0.29	0.294
Solyc05g010560	Unknown Protein	35.2	220	0.001	0.04	-1.63	0.026	-1.15	0.247	-1.42	0.064	-0.31	0.261
Solyc04g054310	Alanine-glyoxylate aminotransferase	13.1.1.3.11	498	-0	-0.06	2.02	0.024	1.24	0.079	1.63	0.061	0.41	0.129
Solyc06g005560	Expansin-1	10.7	545	-0	-0.04	1.79	0.022	1.25	0.088	1.43	0.032	0.50	0.058
Solyc12g009200	Chlorophyll a-b binding protein, chloroplastic	1.1.1.1	76	-0	-0.04	1.67	0.035	1.28	0.057	1.30	0.085	0.59	0.021
Solyc09g092230	Unknown Protein	35.2	75	0.001	0.028	-1.54	0.028	-1.23	0.009	-1.25	0.084	-0.54	0.038
Solyc01g057910	MYB transcription factor	27.3.25	105	0.001	0.029	-1.51	0.010	-1.18	0.097	-1.28	0.071	-0.48	0.07
Solyc06g082750	50S ribosomal protein L17	29.2.1.1.1.2.17	642	0	-0.03	1.52	0.016	1.14	0.045	1.33	0.045	0.51	0.052
Solyc03g078190	Unknown Protein	35.2	51	0	0.054	-3.53	0.002	-1.62	0.229	-2.18	0.107	-0.36	0.187
Solyc10g045100	Phosphoglycerate mutase	4.1.12	104	0	0.068	-3.04	0.002	-1.43	0.069	-2.12	0.015	-0.49	0.064
Solyc12g099790	Calcium-dependent protein kinase 17	29.4	4	0	0.067	-2.48	0.001	-1.51	0.027	-1.65	0.056	-0.56	0.03
Solyc05g049970	Mitogen-activated protein kinase 4	29.4	434	0	0.039	-1.91	0.009	-1.22	0.149	-1.57	0.039	-0.28	0.312
Solyc01g090330	Unknown Protein	35.2	131	0	0.089	-3.90	0.004	-1.53	0.015	-2.54	0.017	-0.34	0.215

Supplementary Table S2. *Auxin-related cis-acting regulatory elements*

The 1500bp 5'-upstream region of the tomato genes SlARF9, SlIAA2 and SlIAA14 were analysed by PlantCARE (Lescot et al., 2002) and PLACE (Higo et al., 1999). The position of the elements from the translation start site (TSS) is indicated.

promoter	element	sequence	+/- strand	position from TSS
SlARF9	AuxRE	TGTCNC	+	-1385
	AuxRE	TGTCTN	-	-773
	NTBBF1ARROLB	ACTTTA	+	-1108
	NTBBF1ARROLB	ACTTTA	+	-180
	NTBBF1ARROLB	ACTTTA	+	-173
SlIAA2	AuxRE	TGTCNC	+	-527
	AuxRE	TGTCTN	+	-846
	AuxRE	TGTCTN	+	-552
	AuxRE	TGTCTN	-	-513
	CATATGGMSAUR	CATATG	+	-819
	CATATGGMSAUR	CATATG	+	-218
	NTBBF1ARROLB	ACTTTA	+	-1054
	NTBBF1ARROLB	ACTTTA	+	-763
	NTBBF1ARROLB	ACTTTA	-	-1034
	NTBBF1ARROLB	ACTTTA	-	-1009
	NTBBF1ARROLB	ACTTTA	-	-963
	ASF1MOTIFCAMV	TGACG	+	-1000
	AuxRR-core	GGTCCAT	+	-682
	TGA-element	AACGAC	+	-1066
SlIAA14	AuxRE	TGTCNC	-	-675
	AuxRE	TGTCTN	-	-366
	NTBBF1ARROLB	ACTTTA	+	-1087

Supplementary Table S3. SlARF9 *expression during tomato fruit set*

Stage	Relative expression[a,b]
Anthesis	-2.300 (A)
3-4 mm	0.808 (C)
5-6 mm	-0.380 (BC)
7-8 mm	-0.697 (B)
13-14 mm	-1.560 (AB)

[a], 2log transformed relative expression data as presented in figures 3A and 3B were used for statistical analysis; [b], Capital characters between brackets indicate homogenous categories (Tukey) with differences at $P<0.05$.

Supplementary Table S4. *Leading edge subsets from the GSEA comparing the transcriptomes of the* SlARF9-*OE and* SlARF9-*RNAi lines*

List of genes that contributed to the enrichment score of the four gene sets (17.3.1, brassinosteroid synthesis and degradation; 17.3.1.2, brassinosteroid synthesis and degradation (sterols); 22.1, polyamine synthesis; 31.5, cell death). The FDR-value for each subset is indicated in parentheses. For each gene, the fold change in expression and correlation to *SlARF9* transcript levels are shown.

**MapMan Bin (FDR)**				**ID**	**ITAG 2.3 Hit description**	**OE vs RNAi**		**OE vs WT**		**RNAi vs WT**		**Pearson correlation to *SlARF9***	
17.3.1 (0.204)	17.3.1.2 (0.221)	22.1 (0.160)	31.5 (0.232)			**Fold change**	***P*-value**	**Fold change**	***P*-value**	**Fold change**	***P*-value**	**r**	***P*-value**
	■			solyc06g082980	3-beta-hydroxysteroid-Delta8 Delta7-isomerase	-1.10	0.073	1.00	0.946	1.11	0.244	-0.35	0.202
	■			solyc05g008680	24-sterol C-methyltransferase	-1.08	0.464	1.10	0.849	1.19	0.305	-0.45	0.092
	■			solyc04g077440	Squalene monooxygenase	-1.05	0.226	1.04	0.610	1.10	0.346	-0.05	0.856
	■			solyc09g009040	Delta14-sterol reductase	-1.05	0.600	-1.05	0.702	1.01	0.979	0.14	0.628
	■			solyc00g085070	Squalene monooxygenase	-1.05	0.394	1.08	0.588	1.13	0.064	-0.07	0.794
■				solyc11g006300	3-oxo-5-alpha-steroid 4-dehydrogenase family protein	-1.61	0.228	-1.97	0.090	-1.22	0.671	-0.27	0.325
■				solyc10g086500	3-oxo-5-alpha-steroid 4-dehydrogenase family protein expressed	-1.31	0.014	1.14	0.501	1.49	0.022	-0.35	0.205
■	■			solyc06g074090	Sterol reductase	-1.19	0.010	-1.07	0.647	1.11	0.334	-0.44	0.098
■	■			solyc02g030170	FAD linked oxidase domain protein	-1.16	0.160	1.01	0.979	1.18	0.191	-0.35	0.207
■	■			solyc02g069490	FAD linked oxidase domain protein	-1.21	0.024	1.02	0.861	1.24	0.082	-0.58	0.023
■	■			solyc02g063240	Sterol C-5 desaturase	-1.16	0.224	1.09	0.709	1.27	0.188	-0.47	0.076
■	■			solyc02g086180	Sterol C-5 desaturase	-1.19	0.032	-1.01	0.981	1.18	0.193	-0.45	0.092
■	■			solyc10g080150	24-sterol C-methyltransferase	-1.25	0.232	-1.07	0.690	1.17	0.564	-0.13	0.644
			■	solyc02g077780	Necrotic spotted lesions 1 (Fragment)	-1.18	0.302	1.08	0.638	1.27	0.332	-0.39	0.156
			■	solyc09g018030	Necrotic spotted lesions 1 (Fragment)	-1.18	0.140	-1.53	0.055	-1.30	0.088	-0.35	0.206
			■	solyc01g005220	Necrotic spotted lesions 1 (Fragment)	-1.53	0.161	1.40	0.166	2.14	0.126	-0.37	0.180
			■	solyc06g068850	Genomic DNA chromosome 5 TAC clone K22G18	-1.15	0.163	-1.22	0.266	-1.06	0.518	-0.41	0.125

Supplementary Table S4. continued

			■	solyc02g094130	Genomic DNA chromosome 5 P1 clone MJC20	-1.16	0.174	1.07	0.679	1.24	0.198	-0.51	0.051
			■	solyc01g103490	Necrotic spotted lesions 1 (Fragment)	-1.13	0.190	1.02	0.937	1.15	0.341	-0.34	0.221
			■	solyc10g076340	Kelch-like protein	-1.27	0.290	-1.24	0.412	1.02	0.968	-0.34	0.220
			■	solyc04g048950	Necrotic spotted lesions 1 (Fragment)	-1.16	0.088	-1.03	0.784	1.13	0.164	-0.37	0.179
			■	solyc02g071780	Necrotic spotted lesions 1 (Fragment)	-1.48	0.070	1.06	0.896	1.57	0.194	-0.65	0.009
			■	solyc10g085710	Necrotic spotted lesions 1 (Fragment)	-1.16	0.122	1.05	0.719	1.21	0.179	-0.40	0.137
			■	solyc09g098070	Necrotic spotted lesions 1 (Fragment)	-1.15	0.230	-1.08	0.317	1.06	0.800	-0.43	0.111
		■		solyc10g054440	Arginine decarboxylase	-1.23	0.244	1.12	0.575	1.37	0.237	-0.18	0.532
		■		solyc02g089610	S-adenosylmethionine decarboxylase proenzyme	-1.32	0.078	-1.35	0.503	-1.03	0.808	-0.34	0.221
		■		solyc01g110440	Arginine decarboxylase	-1.23	0.232	1.08	0.617	1.34	0.278	-0.17	0.539
		■		solyc05g005710	Spermidine synthase	-1.18	0.036	-1.02	0.911	1.16	0.200	-0.33	0.234
		■		solyc01g080380	S-adenosylmethionine decarboxylase proenzyme	-1.17	0.107	1.11	0.324	1.29	0.122	-0.32	0.252
		■		solyc03g007240	Spermidine synthase 1	-1.18	0.128	-1.10	0.738	1.07	0.579	-0.16	0.567
		■		solyc06g054460	S-adenosylmethionine decarboxylase proenzyme	-1.28	0.125	1.13	0.370	1.44	0.170	-0.32	0.246
